# Comparative efficacy and safety of transarterial treatment strategies for hepatocellular carcinoma: a systematic review and network meta-analysis of randomized controlled trials

**DOI:** 10.3389/fmed.2026.1881070

**Published:** 2026-08-24

**Authors:** Jiajun Chen, Ganfa Huang, Fengyi Mo, Zijun Liao, Chang Zhao

**Affiliations:** Department of Interventional Therapy, Guangxi Medical University Cancer Hospital, Nanning, Guangxi, China

**Keywords:** drug-eluting bead, hepatocellular carcinoma, network meta-analysis, randomized controlled trial, transarterial chemoembolization, transarterial radioembolization

## Abstract

**Background:**

Transarterial therapy is widely used for unresectable or intermediate-stage hepatocellular carcinoma (HCC), but the comparative efficacy and safety of available techniques remain uncertain. We compared the efficacy and safety of conventional TACE (cTACE), drug-eluting bead TACE (DEB-TACE), degradable starch microsphere TACE (DSM-TACE), balloon-occluded TACE (B-TACE), transarterial embolization (TAE), and transarterial radioembolization (TARE).

**Methods:**

We performed a PROSPERO-registered systematic review and network meta-analysis of randomized controlled trials. PubMed, Embase, Web of Science, CENTRAL, and CNKI were searched from inception to May 1, 2026. The primary efficacy outcome was objective response rate (ORR). Random-effects network meta-analysis was used. Risk of bias was assessed with RoB 2, and certainty of evidence with CINeMA.

**Results:**

Fifty-six randomized trials involving 5,083 patients were included; 48 trials (4,282 patients) contributed to the ORR network. B-TACE yielded an ORR risk ratio (RR) of 3.50 versus cTACE (95% CI 1.92–6.36, *p* < 0.001), but this estimate arose from three small trials, so is hypothesis-generating. DEB-TACE improved ORR versus cTACE (RR 1.36, 95% CI 1.27–1.47, *p* < 0.001). The TARE estimate was compatible with no effect (RR 1.23, 95% CI 0.98–1.53, *p* = 0.069). DEB-TACE was associated with less nausea or vomiting (cTACE vs. DEB-TACE: OR 1.58, 95% CI 1.19–2.11, *p* = 0.002), abdominal pain (OR 1.55, 95% CI 1.08–2.22, *p* = 0.017), and fever (OR 1.47, 95% CI 1.12–1.93, *p* = 0.006).

**Conclusion:**

Among currently available randomized evidence, DEB-TACE showed the most consistent overall benefit compared with cTACE, improving tumor response while reducing common post-embolization symptoms. The apparently large B-TACE ORR effect should be considered hypothesis-generating because it is based on sparse, clinically indirect evidence. Comparisons of DSM-TACE, B-TACE, TAE, and TARE with cTACE remain insufficient to establish a definitive overall advantage.

**Systematic review registration:**

PROSPERO registration: CRD420261392214.

## Introduction

Hepatocellular carcinoma (HCC) is the most common form of primary liver cancer, accounting for approximately 90% of cases, and remains a major cause of cancer-related death worldwide (1). GLOBOCAN 2022 estimated approximately 866,000 new cases and 758,000 deaths from liver cancer each year, making liver cancer the sixth most common cancer and the third leading cause of cancer death globally (2). This high mortality highlights the aggressive nature of HCC and the continuing need for more effective and better individualized treatment strategies.

Treatment selection for HCC is mainly guided by the Barcelona Clinic Liver Cancer (BCLC) staging system, together with tumor burden, liver function, performance status, and expected clinical benefit (3). Recent international and Chinese guidelines emphasize that treatment should not be determined by stage alone, but should be individualized through multidisciplinary assessment and adjusted according to changes in tumor behavior, liver function, and treatment response (1, 3–5). For very early and early-stage HCC, resection, transplantation, and local ablation remain the main potentially curative options. For advanced disease, systemic therapy has become central to care (1, 4, 6, 7). Intermediate-stage HCC, particularly BCLC stage B disease, remains more difficult to manage because patients vary widely in tumor extent, liver reserve, vascular anatomy, and tumor blood supply (3, 8).

Transarterial chemoembolization (TACE) has long been recommended as the standard treatment for intermediate-stage HCC (1, 3, 9, 10). However, this disease stage is highly heterogeneous and includes patients with large solitary tumors, limited intrahepatic nodules, multifocal bilobar disease, and varying degrees of liver impairment (8). These differences affect tolerance of embolization, local drug delivery, treatment-related ischemia, and liver injury. Therefore, grouping distinct transarterial treatments under broad terms such as “TACE” or “locoregional therapy” is increasingly insufficient for evidence-based treatment selection (1, 3, 8).

Classic conventional TACE (cTACE) is best understood as selective arterial delivery of a lipiodol-chemotherapy emulsion followed by particulate embolization, commonly with gelatin sponge, whereas bland transarterial embolization (TAE) omits the chemotherapy component. Historical nomenclature is inconsistent: lipiodol plus an embolizer without chemotherapy has sometimes been labeled TAE, while lipiodol plus chemotherapy plus an embolizer represents lipiodol-based TACE (11–14). This distinction is clinically important because chemotherapy agents, emulsion preparation, embolic materials, catheter selectivity, and retreatment schedules can modify both efficacy and toxicity.

Drug-eluting bead or microsphere TACE (DEB-TACE/DEM-TACE) was developed to provide more sustained local drug delivery and reduce systemic chemotherapy exposure. However, randomized studies and meta-analyses have shown inconsistent survival advantages over cTACE, with benefits more often observed for tumor response and safety among selected patients (15–17). Degradable starch microsphere TACE (DSM-TACE) provides temporary embolization and may be useful for repeated treatment or in patients with limited liver reserve, although robust randomized evidence remains limited (18, 19). Balloon-occluded TACE (B-TACE) uses temporary arterial balloon occlusion to improve drug and lipiodol deposition within the tumor. Early evidence suggests improved radiologic response, but the available remains limited and requires systematic evaluation (20, 21).

Other transarterial strategies differ further in mechanism. Yttrium-90 transarterial radioembolization (TARE) delivers internal radiation rather than relying mainly on arterial blockage, and may improve local control or progression-related outcomes in selected patients, including those with intermediate-stage disease or portal vein tumor thrombosis (22, 23). Transarterial embolization (TAE) induces tumor necrosis by blocking arterial blood supply without chemotherapy. Previous randomized evidence suggests that, in some patients, TAE may provide survival outcomes comparable to chemoembolization while avoiding chemotherapy-related toxicity (9, 24).

Previous network meta-analyses (NMAs) have addressed transarterial therapy for HCC, but they answer different clinical questions. Katsanos et al. synthesized 55 randomized trials involving 5,763 patients, searched through June 2017, across nine nodes that included cTACE, DEB-TACE, bland TAE, TARE, and several combinations with radiotherapy, ablation, or systemic treatment (25). That network substantially overlaps with the present review for established monotherapies but did not evaluate DSM-TACE or B-TACE. Later NMAs have mainly compared TACE-based combinations, interventional multimodality strategies, or TACE combined with molecularly targeted agents (26–29). Their breadth confirms that the comparative TACE literature is extensive, while leaving a narrower procedure-focused question unresolved: when the randomized difference between groups is the transarterial platform itself, how do the principal embolization, drug-delivery, and radioembolization strategies compare?

To address this evidence gap, we conducted a systematic review and network meta-analysis of randomized controlled trials comparing cTACE, DEB/DEM-TACE, DSM-TACE, B-TACE, TAE, and TARE for HCC. We assessed objective response, disease control, progression, and common post-embolization symptoms. By combining direct and indirect evidence, this study aimed to clarify the relative efficacy and safety of available transarterial strategies and provide a more practical evidence base for treatment selection in unresectable or intermediate-stage HCC (1, 3–5, 16, 20, 24).

## Methods

### Study design and reporting standards

This study was a systematic review and network meta-analysis of randomized controlled trials comparing the efficacy and safety of different transarterial treatment strategies for HCC. The protocol was registered in PROSPERO (CRD420261392214). Reporting followed the PRISMA 2020 statement and the PRISMA extension for network meta-analyses (30, 31). We restricted the network to randomized trials to prioritize internal validity for comparative efficacy, recognizing that this decision was likely to produce a sparser network and less informative data for uncommon or delayed harms. Because transarterial therapy remains a key treatment option for unresectable or intermediate-stage HCC in current EASL, AASLD, and BCLC frameworks, we focused on transarterial strategies that could be connected within an evidence network (1, 3, 4).

### Search strategy and study selection

We searched PubMed, Embase, Web of Science, the Cochrane Central Register of Controlled Trials, and the Chinese National Knowledge Infrastructure database (CNKI) from inception to May 1, 2026. Search terms covered hepatocellular carcinoma, transarterial therapy, and randomized controlled trials, including “hepatocellular carcinoma,” “HCC,” “transarterial chemoembolization,” “TACE,” “drug-eluting microsphere,” “drug-eluting bead,” “DEB-TACE,” “DEM-TACE,” “degradable starch microspheres,” “DSM-TACE,” “balloon-occluded TACE,” “B-TACE,” “radioembolization,” “TAE,” “TARE,” and “randomized controlled trial.” Equivalent Chinese and English terms were used for CNKI. We also screened Google Scholar, reference lists of eligible studies, and relevant reviews and guidelines. The complete search strategy is provided in Appendix 2. Two reviewers independently screened titles and abstracts, assessed full texts, and determined eligibility. Disagreements were resolved by a third reviewer.

### Eligibility criteria

Studies were eligible if they met the following criteria: patients had HCC diagnosed by imaging, pathology, or accepted clinical guideline criteria; the study was a randomized controlled trial; at least two transarterial strategies were compared; and at least one extractable efficacy or safety outcome was reported. Eligible interventions included cTACE, DEB-TACE, DSM-TACE, B-TACE, TAE, and TARE. Studies that included systemic therapy, ablation, surgery, or other non-transarterial treatments were included only when these co-interventions were balanced between randomized groups and the transarterial strategy was the only difference. Observational studies, non-randomized studies, single-arm studies, duplicate publications, studies without extractable data, and studies with mixed tumor populations in which HCC data could not be separated were excluded. Two early B-TACE trials enrolled hypovascular or mixed liver-tumor populations that were not fully separable; and they were retained in the primary network but flagged as clinically indirect, and a *post hoc* sensitivity analysis excluded them. cTACE versus TAE reports with incompatible response definitions or insufficient randomization information were discussed narratively and did not contribute to network estimates.

### Data extraction and outcomes

Two reviewers independently extracted data, and discrepancies were resolved by a third reviewer. Extracted information included first author, year of publication, country or region, study design, sample size, treatment regimen, age, sex, HBV infection, BCLC stage, Child-Pugh class, ECOG performance status, alpha-fetoprotein level (AFP), maximum tumor diameter, tumor number, follow-up duration, radiologic response, and adverse events.

The primary efficacy outcome was objective response rate (ORR). Secondary efficacy outcomes included disease control rate (DCR), complete response (CR), partial response (PR), stable disease (SD), and progressive disease (PD). Tumor response was extracted according to the criteria prespecified in each trial, including modified RECIST (mRECIST), RECIST, or equivalent imaging-based criteria (32). Safety outcomes focused on common post-embolization syndrome-related events, including nausea or vomiting, abdominal pain, and fever, because these were the adverse events reported with sufficient consistency across the network to permit quantitative synthesis.

### Risk of bias and certainty of evidence

Risk of bias was assessed using the Cochrane RoB 2 tool. The assessment covered five domains: the randomization process, deviations from intended interventions, missing outcome data, outcome measurement, and selective reporting. An overall risk-of-bias judgment was assigned for each trial (33). Certainty of evidence was assessed with the CINeMA (https://cinema.med.auth.gr/) framework across six domains: within-study bias, reporting bias, indirectness, imprecision, heterogeneity, and incoherence (34, 35).

### Statistical analysis

We performed a random-effects network meta-analysis. For ORR, DCR, CR, PR, SD, treatment effects were expressed as risk ratios (RRs) with 95% confidence intervals (CIs). For PD and safety outcomes, effects were expressed as odds ratios (ORs) with 95% CIs. Two-sided *p* values were reported for key contrasts. Network plots were used to show treatment nodes and direct comparisons (47). Node size was weighted by sample size, and line thickness was weighted by the number of direct comparisons (36). Treatment rankings were summarized using the surface under the cumulative ranking curve (SUCRA), it reflected the comprehensive ranking advantage of this intervention measure and probability of being the best treatment, and mean rank. Rankings were interpreted as supportive rather than definitive when evidence was sparse, estimates were imprecise, or certainty was low (37). All analyses and figures were generated using Stata version 19.

#### Sensitivity, subgroup, and meta-regression analyses

We assessed transitivity by comparing potential effect modifiers across studies, including publication period, BCLC stage, Child-Pugh class, tumor size, and follow-up (Appendix 3, Table S3.2), as well as age, sex, viral etiology, ECOG status, AFP, and tumor number when reported. Global inconsistency was assessed using the design-by-treatment interaction model, and local inconsistency was examined using node-splitting analysis. *p* < 0.05 was considered suggestive of potential inconsistency. Network heterogeneity was estimated using tau-squared.

Several sensitivity analyses were performed to test robustness. These included analyses restricted the ORR network to trials published from 2010 onward, English-language reports, and trials at overall low risk of bias; a *post hoc* analysis excluded the two clinically indirect hypovascular or mixed-tumor B-TACE studies, analyses restricted to trials with an overall low risk of bias, analyses excluding treatment nodes supported by two or fewer studies, analyses excluding studies with “some concerns” in key RoB 2 domains, and re-interpretation of the primary outcomes according to heterogeneity and inconsistency findings. Exploratory analyses included univariable meta-regression using study-level covariates and subgroup analysis according to the proportion of patients with Child-Pugh class A liver function. Direct DEB-TACE-versus-cTACE comparisons, chemotherapy class and microsphere class were examined as potential effect modifiers. Small-study effects and potential publication bias were assessed using comparison-adjusted funnel plots (36). All subgroup and meta-regression analyses were interpreted as exploratory because they rely on study-level rather than patient-level information and are vulnerable to ecological bias.

## Results

### Study selection and trial characteristics

**Figure 1:**
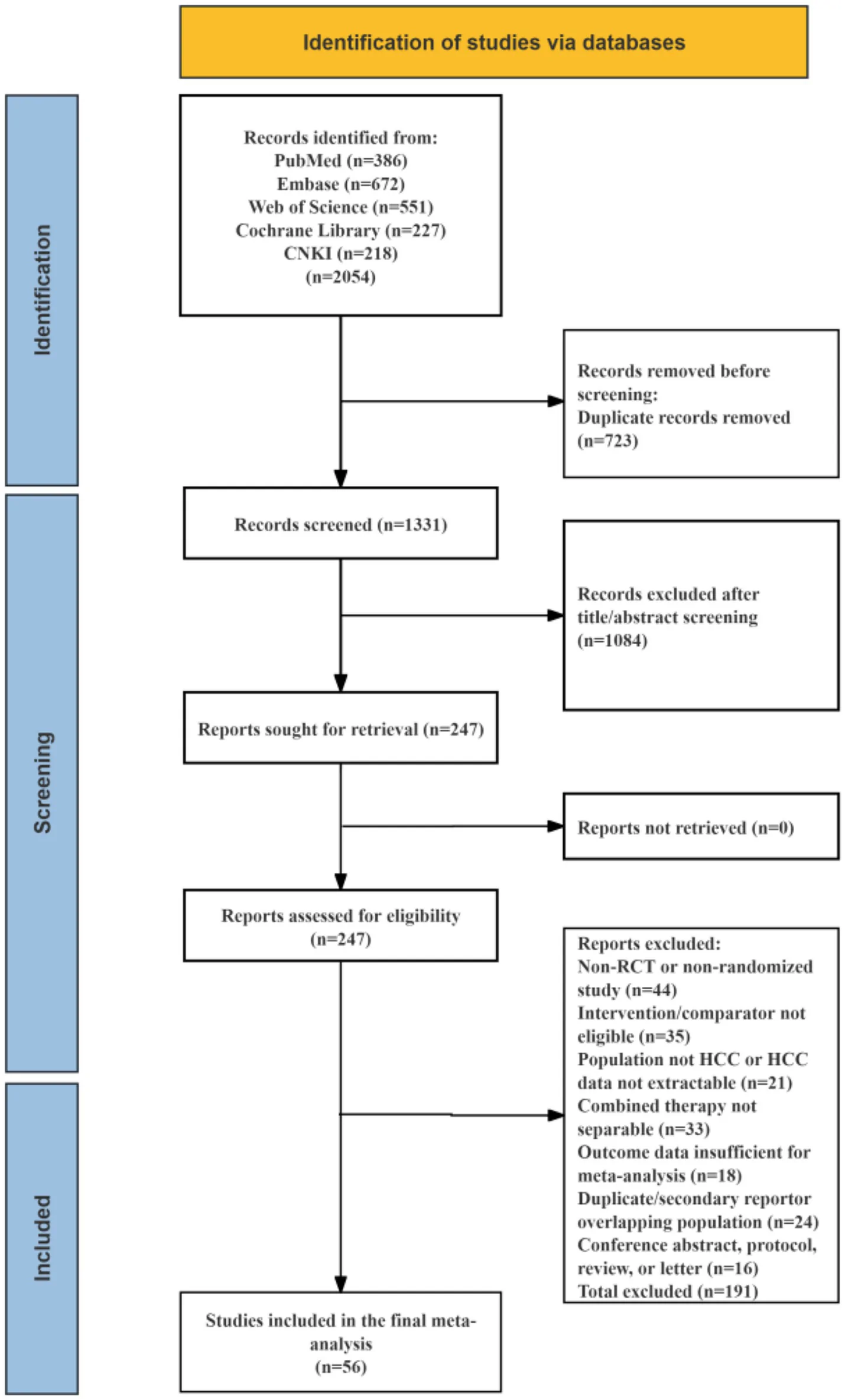
Study selection flow diagram. PRISMA flow diagram showing identification, screening, eligibility assessment, and inclusion of randomized controlled trials in the final network meta-analysis.

This systematic review and network meta-analysis was conducted according to the PRISMA-NMA framework (Appendix 1). Searches covered PubMed, Embase, Web of Science, CENTRAL, and CNKI (Appendix 2). A total of 56 randomized controlled trials involving 5,083 patients with HCC were included (Figure 1), with publication years ranging from 1992 to 2026. The included studies compared six transarterial treatment strategies: cTACE, DEB-TACE, DSM-TACE, B-TACE, TAE, and TARE. Most studies were conducted in China, with additional studies from Europe, North America, Japan, and other regions. Individual trial sample sizes ranged from 24 to 289 patients, with a median follow-up of approximately 12 months and a follow-up range of approximately 1 to 51 months (Appendix 3, Table S3.1). Prespecified extracted variables included study design, sample size, demographic characteristics, HBV status, BCLC stage, Child-Pugh class, ECOG score, AFP level, tumor size and number, follow-up duration, efficacy outcomes, and post-embolization syndrome-related adverse events (Appendix 4). However, trial populations and procedures were heterogeneous: arm-level BCLC stage, Child-Pugh class, tumor size, publication period, and follow-up are summarized quantitatively in Appendix 3, Table S3.2. Reported central tumor-size values were not confined to small HCC and ranged from 3.85 to 13.45 cm in cTACE arms, 4.10 to 13.16 cm in DEB-TACE arms, and 8.60 to 9.10 cm in B-TACE arms; tumor size was nevertheless unreported in many arms. These data preclude restricting the evidence base to tumors <3–4 cm but also prevent reliable tumor-size-specific treatment conclusions.

But evidence supporting the DSM-TACE and B-TACE nodes was sparse. DSM-TACE was represented by two randomized trials published in 2020 and 2021, comprising 121 randomized participants, of whom 63 received DSM-TACE; both trials were unavailable when the 2017 Katsanos review was conducted. B-TACE was represented by three randomized trials comprising 214 participants, of whom 104 received B-TACE. Overall, three post-2017 randomized trials comprising 181 participants newly informed the DSM-TACE and B-TACE nodes. Trial-level details are provided in Appendix 17, Table S17.1–2.

### Risk of bias, certainty of evidence, and consistency

Among the 56 randomized trials, 10 were judged to be at overall low risk of bias, whereas 46 had “some concerns”; no study was judged to be at overall high risk of bias. The main limitations were insufficient reporting of randomization and blinding procedures, together with uncertainty in outcome measurement and selective reporting for some outcomes (Appendix 5, Figures S5.1–S5.2). Global inconsistency tests showed no statistically significant inconsistency for the primary outcomes: *p* = 0.9513 for ORR. Most secondary outcomes likewise showed no clear evidence of inconsistency. The global test for SD approached the prespecified threshold (*p* = 0.0843), suggesting that this outcome should be interpreted with greater caution. Overall network heterogeneity was low, with τ^2^ values for ORR, DCR, CR, PR, PD, 0.0153, 0.0028, 0.0355, <0.001, 0.0429, respectively. Heterogeneity was relatively higher for abdominal pain (τ^2^ = 0.2819) (Appendix 6, Table S6.1). Node-splitting analyses did not identify significant local inconsistency, with all major local comparisons having *p* values greater than 0.05 (Appendix 6, Tables S6.2–S6.10). CINeMA assessment indicated low certainty of evidence for all network comparisons for ORR (Appendix 10, Tables S10.1, S10.9). A small number of comparisons for DCR, PD, nausea or vomiting, and fever reached moderate or high certainty; however, the overall evidence base remained limited by sparse nodes, imprecision, and risk-of-bias concerns in some studies (Appendix 10, Figures S10.1–S10.18, Tables S10.1–S10.9). Comparison-adjusted funnel plots showed no clear evidence of small-study effects or publication bias for ORR, DCR, CR, PR, SD, PD, nausea or vomiting, abdominal pain, fever (Appendix 11, Figures S11.1–S11.9).

### Objective response rate

**Figure 2:**
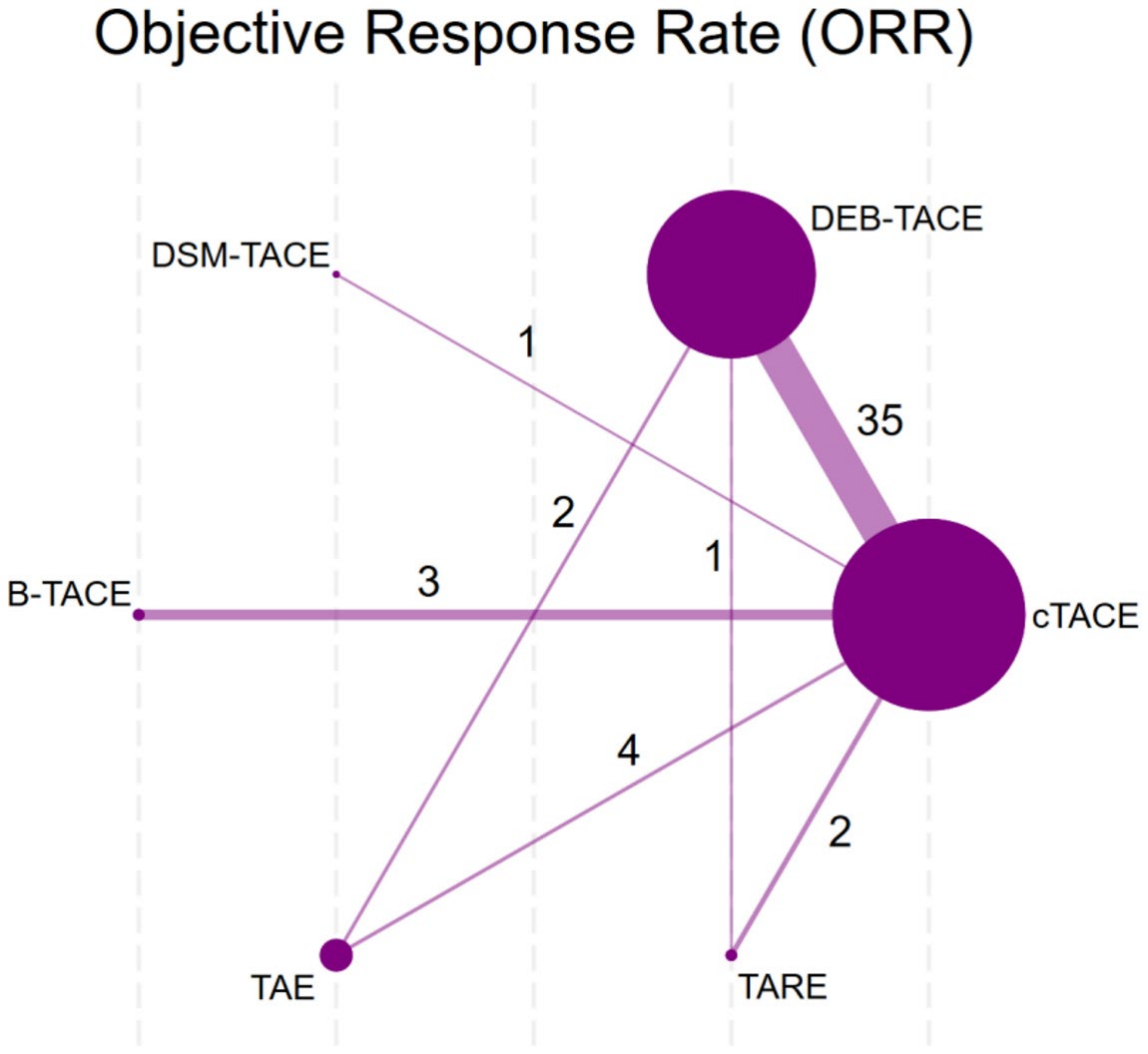
Network plot for objective response rate. Each node represents a transarterial treatment strategy, and node size is proportional to the number of participants. Line thickness is proportional to the number of direct comparisons between treatments.

**Figure 3:**
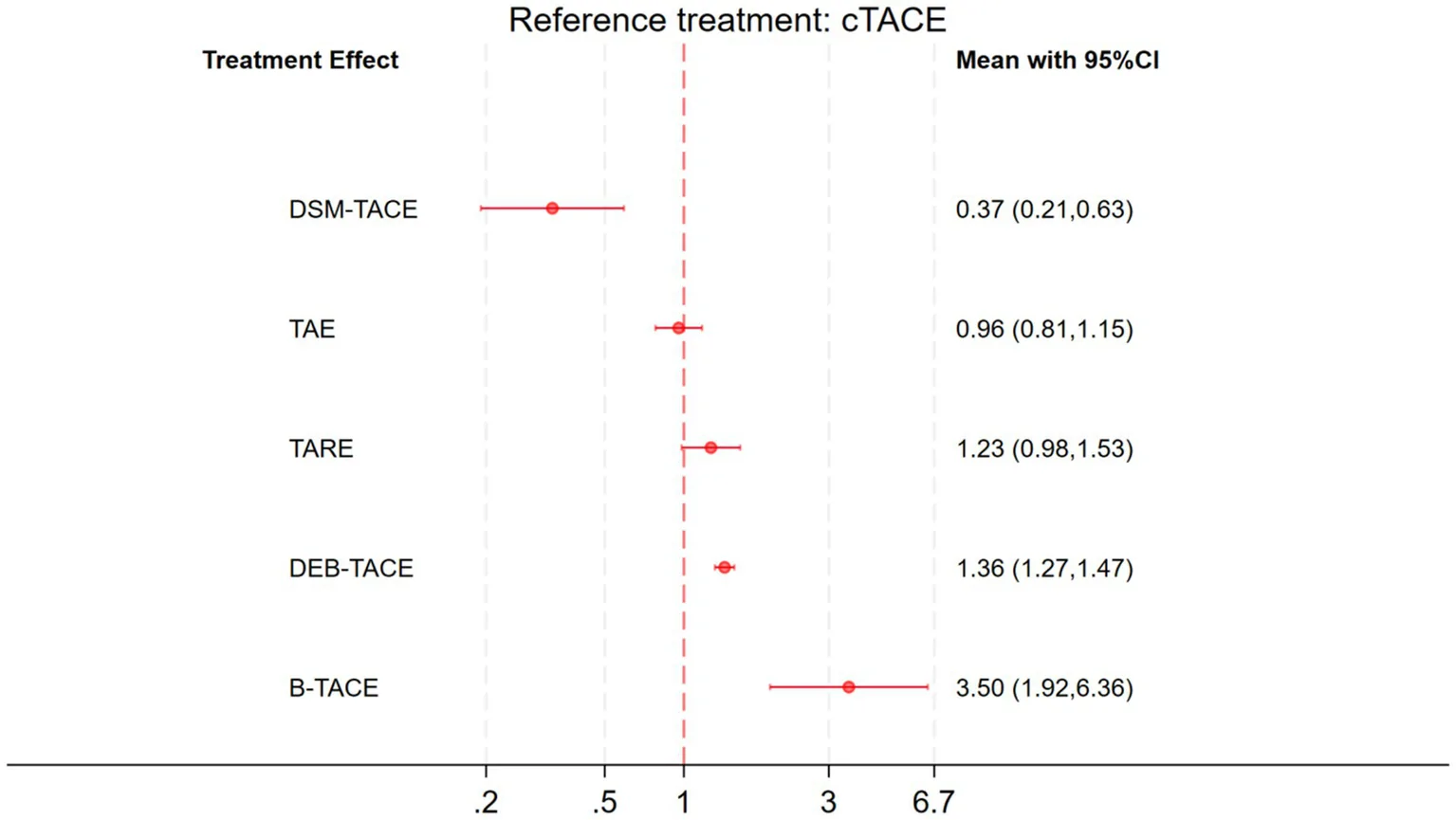
Forest plot of objective response rate compared with cTACE. Effect estimates are presented as risk ratios with 95% confidence intervals. cTACE was used as the reference treatment.

The ORR network included 48 studies, 96 treatment arms, and 4,282 patients, with all six treatment nodes represented (Figure 2). B-TACE ranked highest for ORR, with a SUCRA of 99.9% and a 99.8% probability of being best; but its primary estimate was based on three small trials, two involving hypovascular and mixed liver-tumor populations, and is therefore hypothesis-generating rather than confirmatory. DEB-TACE ranked second (SUCRA 76.5%), followed by TARE (SUCRA 61.9%) (Appendix 8, Figure S8.1, Table S8.1). Compared with cTACE, B-TACE significantly improved ORR (RR 3.50, 95% CI 1.92–6.36, *p* < 0.001), and DEB-TACE also showed a consistent advantage (RR 1.36, 95% CI 1.27–1.47, *p* < 0.001). The point estimate for TARE versus cTACE also favored improved ORR, but the CI crossed the line of no effect (RR 1.23, 95% CI 0.98–1.53, *p* = 0.069) (Figure 3).

### Secondary efficacy outcomes

In the DCR network, B-TACE and DEB-TACE ranked highest, with SUCRA values of 98.9 and 74.9%, respectively (Appendix 7, Figure S7.1; Appendix 8, Figure S8.2, Table S8.2). The DCR RR for cTACE versus DEB-TACE was 0.86 (95% CI 0.83–0.90, *p* < 0.001), and for cTACE versus B-TACE was 0.68 (95% CI 0.54–0.86, *p* = 0.001) (Appendix 9, Table S9.2). For CR, DSM-TACE and B-TACE had SUCRA values of 78.9 and 77.8%, respectively (Appendix 7, Figure S7.2; Appendix 8, Figure S8.3, Table S8.3), although evidence for DSM-TACE was sparse and should be interpreted cautiously. The CR RR for cTACE versus DEB-TACE was 0.68 (95% CI 0.59–0.79, *p* < 0.001), supporting a higher CR rate with DEB-TACE (Appendix 9, Table S9.3). For PR, B-TACE and DSM-TACE ranked highly, with SUCRA values of 89.2 and 84.7%, respectively (Appendix 7, Figure S7.3; Appendix 8, Figure S8.4, Table S8.4). The PR RR for cTACE versus DEB-TACE was 0.77 (95% CI 0.71–0.84, *p* < 0.001) (Appendix 9, Table S9.4). For SD, cTACE ranked highest, with a SUCRA of 91.6% (Appendix 7, Figure S7.4; Appendix 8, Figure S8.5, Table S8.5); however, this outcome more likely reflects an intermediate state in which tumors have neither responded nor progressed, and should not be interpreted in isolation as a treatment advantage. For PD, B-TACE, DSM-TACE, and DEB-TACE ranked favorably (Appendix 7, Figure S7.5; Appendix 8, Figure S8.6, Table S8.6). The PD OR for cTACE versus DEB-TACE was 2.65 (95% CI 2.10–3.33, *p* < 0.001), and for cTACE versus B-TACE was 4.52 (95% CI 2.03–10.09, *p* < 0.001) (Appendix 9, Table S9.6). Pairwise forest plots for DCR, CR, PR, SD, and PD were broadly consistent with these directional findings (Appendix 16, Figures S16.2–S16.6). However, the comparisons involved in B-TACE and DSM-TACE are based solely on limited randomized controlled evidence and should be regarded as exploratory conclusions.

### Safety outcomes

The nausea or vomiting network was mainly composed of cTACE, DEB-TACE, DSM-TACE, and TAE, with insufficient evidence for DSM-TACE and TAE (Appendix 7, Figure S7.6). SUCRA rankings for nausea or vomiting reflected the probability of event occurrence rather than clinical safety benefit; therefore, higher rankings should be interpreted as indicating a higher risk of adverse events (Appendix 8, Figure S8.7, Table S8.7). The OR for nausea or vomiting for cTACE versus DEB-TACE was 1.58 (95% CI 1.19–2.11, *p* = 0.002) (Appendix 9, Table S9.7). In the abdominal pain network, cTACE, DEB-TACE, and TAE contributed most of the evidence, whereas other treatment nodes were sparse (Appendix 7, Figure S7.7). Rankings for abdominal pain events showed higher SUCRA values for TARE, B-TACE, and DSM-TACE, but the precision of the evidence was limited (Appendix 8, Figure S8.8, Table S8.8). The OR for abdominal pain for cTACE versus DEB-TACE was 1.55 (95% CI 1.08–2.22, *p* = 0.017) (Appendix 9, Table S9.8). The fever network was also driven primarily by cTACE, DEB-TACE, and TAE (Appendix 7, Figure S7.8). DSM-TACE had the highest SUCRA for fever events, at 93.4% (Appendix 8, Figure S8.9, Table S8.9). The OR for fever for cTACE versus DEB-TACE was 1.47 (95% CI 1.12–1.93, *p* = 0.006) (Appendix 9, Table S9.9). Crude event summaries showed that the proportions of nausea or vomiting, abdominal pain, and fever were 281/1,301, 340/1,181, and 305/1,225 for DEB-TACE, respectively, compared with 399/1,337, 566/1,479, and 573/1,577 for cTACE (Appendix 12, Table S12.1). Pairwise forest plots for safety outcomes were generally consistent with the network estimates, although CIs were wide for many comparisons not involving cTACE or DEB-TACE (Appendix 16, Figures S16.7–S16.9).

### Sensitivity, regression, and subgroup analyses

When analyses were restricted to trials at overall low risk of bias, the direction of the main efficacy outcomes was generally preserved; however, DSM-TACE was not retained in the low-risk network, suggesting that its findings were largely driven by sparse evidence (Appendix 13, Table S13.1). After exclusion of sparse treatment nodes, the network structures for ORR, PR, PD were largely maintained, whereas the nausea or vomiting network was reduced mainly to comparisons between cTACE and DEB-TACE (Appendix 13, Table S13.2). Sensitivity analyses based on heterogeneity and inconsistency diagnostics suggested that ORR, DCR, CR, and PR were relatively robust. SD required cautious interpretation because the global *p* value approached the prespecified threshold, and abdominal pain was less robust because of greater network heterogeneity (Appendix 13, Table S13.3). After exclusion of studies with “some concerns” in key RoB 2 domains, major non-DSM treatment nodes remained connected, but evidence for safety outcomes was substantially reduced (Appendix 13, Table S13.4). Exploratory meta-regression showed that higher mean age was associated with lower ORR and DCR, with coefficients of −0.445 (95% CI -0.691 to −0.198, *p* < 0.001) for ORR and −0.487 (95% CI -0.753 to −0.220, *p* < 0.001) for DCR (Appendix 14, Table S14.1). The proportion of patients with Child-Pugh A liver function was associated with DCR, with a coefficient of −0.142 (95% CI -0.249 to −0.034, *p* = 0.010), and follow-up duration was also associated with DCR, with a coefficient of −0.175 (95% CI -0.278 to −0.073, *p* = 0.001) (Appendix 14, Table S14.1). In subgroup analyses stratified by liver functional reserve, the network in the lower Child-Pugh class A proportion subgroup was mainly composed of cTACE, DEB-TACE, and TAE, whereas the higher Child-Pugh A proportion subgroup retained a more complete set of treatment nodes, including DSM-TACE, B-TACE, and TARE (Appendix 15, Table S15.1). These subgroup findings suggest that liver functional reserve may influence network comparability and estimate precision, but because several nodes were sparse, the results should be interpreted as exploratory. The DEB-TACE ORR effect remained in post-2010 (RR 1.38, 95% CI 1.30–1.46), English-language (RR 1.34, 95% CI 1.15–1.57), and low-risk-of-bias analyses (RR 1.30, 95% CI 1.05–1.59). Excluding the two clinically indirect hypovascular or mixed-tumor B-TACE studies left one contemporary HCC trial and produced a larger but highly imprecise B-TACE estimate (RR 6.33, 95% CI 2.09–19.17), demonstrating fragility rather than confirming superiority (Appendix 13, Table S13.5). In direct DEB-TACE-versus-cTACE ORR comparisons, exploratory meta-regression detected no effect modification by doxorubicin-based versus other/unclear chemotherapy (ratio of RRs 0.96, 95% CI 0.84–1.09; *p* = 0.513) or CalliSpheres versus other/unspecified microspheres (ratio of RRs 1.02, 95% CI 0.88–1.18; *p* = 0.795). Incomplete class reporting, ecological bias, and limited power mean that these null interactions do not establish equivalence (Appendix 14, Table S14.2).

## Discussion

This systematic review and network meta-analysis included 56 randomized controlled trials and 5,083 patients with HCC across six transarterial strategies. DEB-TACE showed the most reproducible response and common symptom-tolerability advantages over cTACE. B-TACE produced an extreme ORR ranking, but the estimate rests on three small trials with substantial clinical indirectness and is best viewed as hypothesis-generating. DSM-TACE, TAE, and TARE estimates were generally imprecise or compatible with no effect for key comparisons.

The current analysis should be interpreted as complementary to, rather than a replacement for, the 2017 network meta-analysis by Katsanos et al. Their network primarily evaluated mortality across four embolization monotherapies and several combination strategies and did not establish superiority of cTACE, DEB-TACE, or TARE over bland TAE (25). Our network addresses a narrower procedural-platform question, incorporates randomized evidence through May 2026, and focuses predominantly on radiologic response and common post-embolization symptoms; it does not provide a hazard-ratio-based overall-survival update. Numerical effect estimates from the two networks are therefore not directly comparable. The addition of DSM-TACE and B-TACE materially expanded the ranking space: B-TACE ranked first for ORR (SUCRA 99.9%; RR vs. cTACE 3.50, 95% CI 1.92–6.36), whereas DSM-TACE ranked last for ORR (SUCRA 0%; RR vs. cTACE 0.37, 95% CI 0.21–0.63), despite favorable but unstable rankings for selected response components. These new nodes did not alter the principal clinical interpretation: DEB-TACE remained the only platform with consistent response and common symptom-tolerability advantages across a comparatively large evidence base, whereas DSM-TACE and B-TACE remained exploratory. The absence of DSM-TACE from low-risk sensitivity networks and the dependence of B-TACE on a single HCC-specific trial after exclusion of two clinically indirect studies further argue against inferring definitive superiority from treatment rankings.

HCC remains a major cause of cancer-related death worldwide, and many patients are not eligible for curative resection, ablation, or transplantation at diagnosis (2). Current BCLC, AASLD, and EASL guidelines recommend TACE as an important treatment for intermediate-stage or unresectable HCC in patients with preserved liver function, acceptable performance status, and mainly intrahepatic disease (1, 3, 4). The landmark randomized trial by Llovet et al. (9) established the role of transarterial embolization or chemoembolization by showing better outcomes than best supportive care in unresectable HCC. However, TACE is not a single standardized treatment. Outcomes may be influenced by the chemotherapy agent, embolic material, degree of catheter selectivity, retreatment strategy, imaging response criteria, and baseline liver function. The present study addresses this issue by comparing transarterial strategies within a randomized evidence framework.

The benefits of DEB-TACE are clinically plausible. Drug-eluting beads allow gradual local drug release and sustained tumor exposure while reducing systemic drug levels and non-target toxicity. This mechanism may explain the higher tumor response rates and lower risks of nausea or vomiting, abdominal pain, and fever observed with DEB-TACE compared with cTACE. Both the multicenter PRECISION V RCT conducted by Lammer et al. (15) and the Italian multicenter RCT reported by Golfieri et al. (38) demonstrated that DEB-TACE significantly improved the ORR and DCR in patients with HCC, with comparable or better safety profiles. The present network meta-analysis therefore supports DEB-TACE as a strategy with consistent response and tolerability advantages.

B-TACE had the highest ORR ranking, but this result requires especially cautious interpretation. The node comprised three small trials; two enrolled hypovascular or mixed liver-tumor populations rather than a typical hypervascular HCC population, and the 2026 Shan trial included predominantly advanced, large tumors (39). The sensitivity analysis excluding the two older indirect studies left a single trial and a very wide CI. Thus, the extreme SUCRA value reflects sparse evidence and network geometry as much as treatment performance. B-TACE should be considered a hypothesis-generating intensified delivery strategy that requires multicentre HCC-specific trials with standardized response, liver-function.

TARE represents a different transarterial approach. Unlike TACE, which relies on local chemotherapy and reduced tumor blood supply, TARE delivers internal radiation through yttrium-90 microspheres. Previous randomized studies have shown that TARE can delay progression or improve local control (23, 40). In this study, TARE generally showed favorable point estimates for ORR, but CIs crossed the null line. Current randomized evidence is therefore insufficient to confirm a general survival advantage over cTACE or DEB-TACE. In practice, the role of TARE is likely to depend on patient selection, tumor burden, portal vein involvement, center experience, dosimetry, and its integration with transplantation or systemic therapy. It should not be viewed simply as an interchangeable alternative to TACE.

The difference between TAE and TACE also remains uncertain. The randomized trial by Meyer et al. (41) found that TACE improved radiological response compared with TAE and was associated with more high-grade toxicity. Brown et al. (42) similarly found that adding doxorubicin to microsphere embolization did not necessarily improve survival. These findings align with our network, in which TAE did not rank favorably for the main efficacy outcomes. They also suggest that the value of transarterial therapy depends not only on whether chemotherapy is added, but also on the balance between drug delivery, embolization depth, tumor selectivity, and preservation of liver function.

Safety is central to treatment selection in HCC, particularly in patients with cirrhosis. In this study, DEB-TACE was associated with lower risks of nausea or vomiting, abdominal pain, and fever than cTACE. Although post-embolization syndrome is often manageable, it can prolong hospital stay, delay repeat treatment or systemic therapy, and contribute to liver decompensation in patients with limited liver reserve. The more favorable balance between efficacy and tolerability observed with DEB-TACE is therefore clinically relevant. However, our safety analysis mainly captured common post-embolization symptoms. It did not allow consistent comparisons of liver function deterioration, biliary injury, radioembolization-induced liver disease, grade ≥3 adverse events, or treatment-related death, because these were reported too sparsely and inconsistently for comparative synthesis. A complete safety ranking across all clinically important risks was therefore not possible.

The treatment landscape for unresectable or intermediate-stage HCC is changing rapidly. Phase 3 trials such as EMERALD-1 and LEAP-012 have shown that combining TACE with immune checkpoint inhibitors and anti-angiogenic therapy can improve progression-free survival, although at the cost of greater toxicity and treatment complexity (43, 44). These findings strengthen rather than reduce the relevance of this study. In the combination-treatment era, the choice of transarterial platform may affect tumor response, liver function preservation, tolerance of subsequent systemic therapy, and overall treatment sequencing. Future research should standardize TACE techniques, define TACE-unsuitable and TACE-refractory disease more clearly, optimize the timing of systemic therapy, and include dynamic assessment of liver function. To facilitate treatment sequencing, TACE refractoriness should be distinguished from TACE unsuitability. The 2014 JSH-LCSGJ criteria define TACE failure/refractoriness as two consecutive inadequate responses or intrahepatic progressions after adequately performed selective TACE, and also include persistent tumor-marker elevation, vascular invasion, or extrahepatic spread (45). In contrast, the 2019 proof-of-concept study by Kudo et al. supports consideration of upfront systemic therapy in selected patients with Child-Pugh A liver function and tumor burden beyond the up-to-seven criteria, a population often described clinically as TACE-unsuitable (46). Future studies should standardize TACE techniques, prospectively validate selection and switching criteria, optimize systemic-treatment timing, and incorporate dynamic assessment of liver function.

The broader TACE-related network meta-analysis literature addresses a different decision level. Yan et al., Le et al., Zhang et al., and Long et al. compared TACE-based combinations involving local ablation, radiotherapy, hepatic arterial infusion chemotherapy, or molecularly targeted agents (26–29). These networks inform whether and how to add a second modality, but they do not isolate the comparative contribution of the embolic platform. The present network therefore provides complementary information for selecting among procedural transarterial platforms before combination therapy is considered and for identifying where evidence is too sparse to justify platform-specific claims. This distinction is clinically relevant because the arterial platform may influence drug deposition, liver-function preservation, post-embolization symptoms, repeat-treatment feasibility, and subsequent treatment sequencing.

This study has several strengths. First, it included only randomized controlled trials, reducing selection bias compared with observational evidence. Second, it compared six transarterial strategies, and incorporated randomized evidence up to May 1, 2026. Third, the study followed the PRISMA-NMA framework and assessed risk of bias, inconsistency, heterogeneity, certainty of evidence, publication bias, sensitivity analyses, meta-regression, and subgroups analyses.

Several limitations should be considered. First, although all included studies were randomized trials, most had some risk-of-bias concerns, mainly because of incomplete reporting of randomization, blinding, and outcome reporting. Second, the included studies were published across more than three decades, during which staging, imaging criteria, catheter selectivity, chemotherapy, embolic materials, supportive care, and systemic therapy changed substantially. Third, many studies were conducted in China, where HBV-related HCC predominates, and caution is needed when applying the findings to HCV-related, alcohol-related, or metabolic dysfunction-associated steatotic liver disease-related HCC populations. Fourth, transitivity is uncertain because BCLC stage, Child-Pugh class, tumor size, follow-up, and treatment composition were incompletely and unevenly reported; central tumor-size values also extended well beyond 3–4 cm, precluding a small-HCC-only interpretation or reliable size-specific effects. Finally, several treatment nodes, especially DSM-TACE, B-TACE, and TARE, were supported by sparse evidence. SUCRA rankings may therefore be influenced by small samples and imprecise estimates. Clinical interpretation should prioritize effect sizes, CIs, and certainty of evidence rather than rankings alone.

## Conclusion

This systematic review and network meta-analysis of 56 randomized trials found that DEB-TACE improved radiologic response and reduced common post-embolization symptoms compared with cTACE. B-TACE produced a large ORR estimate, but the result is hypothesis-generating because the node is small and clinically indirect. Current randomized evidence is insufficient to establish a definitive overall advantage for DSM-TACE, B-TACE, TAE, or TARE over cTACE. Treatment should be individualized according to tumor burden and vascularity, BCLC stage, liver reserve, anatomy, technical expertise, and subsequent treatment options.

## Data Availability

The original contributions presented in the study are included in the article/Supplementary material, further inquiries can be directed to the corresponding author.
