# Supplementary material for: Comparative efficacy and safety of transarterial treatment strategies for hepatocellular carcinoma: a systematic review and network meta-analysis of randomized controlled trials
Source: Front Med (Lausanne). 2026 Aug 24;13:1881070. doi: 10.3389/fmed.2026.1881070 (PMC13547934; doi:10.3389/fmed.2026.1881070)
Supplement: Supplementary file 1 [file Data_Sheet_1.DOCX]

**Supplementary Appendix**

**Comparative efficacy and safety of transarterial treatment strategies for hepatocellular carcinoma: a systematic review and network meta-analysis of randomized controlled trials**

**Table of contents**

[Appendix 1: PRISMA NMA Checklist	2](#_Toc20531)

[Appendix 2: Search strategy	7](#_Toc24829)

[Appendix 3: Characteristics of included studies	12](#_Toc18970)

[Appendix 4: List of data extracted from the included randomized clinical trials	21](#_Toc23364)

[Appendix 5: Risk of bias of randomized clinical trials	22](#_Toc9591)

[Appendix 6: Evaluation of inconsistency and heterogeneity	24](#_Toc25654)

[Appendix 7: Network maps and forest plots of secondary outcomes	30](#_Toc19618)

[Appendix 8: SUCRA and cumulative probability plots	38](#_Toc28497)

[Appendix 9: League tables of summary estimates for transarterial treatment strategies in the treatment of hepatocellular carcinoma derived from network meta-analysis of 56 included studies	47](#_Toc21916)

[Appendix 10: CINeMA Assessment	56](#_Toc20774)

[Appendix 12: Summary of Adverse Events	99](#_Toc18172)

[Appendix 13: Sensitivity Analysis	102](#_Toc4365)

[Appendix 14: Regression Analysis	111](#_Toc16722)

[Appendix 15: Subgroup Analysis	112](#_Toc4490)

[Appendix 16: Pairwise comparison forest plots	114](#_Toc6268)

[Appendix 17: Comparison with previous TACE-related network meta-analyses and evidence supporting DSM-TACE and B-TACE	123](#_Toc3268)

**Appendix 1: PRISMA NMA Checklist**

| *Section/Topic* | *Item #* | *Checklist Item* | *Reported on Page #* |
| --- | --- | --- | --- |
| **TITLE** |  |  |  |
| Title | 1 | Identify the report as a systematic review incorporating a network meta-analysis (or related form of meta-analysis). | p. 1 |
|  |  |  |  |
| **ABSTRACT** |  |  |  |
| Structured summary | 2 | Provide a structured summary including, as applicable:  Background: main objectives  Methods: data sources; study eligibility criteria, participants, and interventions; study appraisal; and synthesis methods, such as network meta-analysis.  Results: number of studies and participants identified; summary estimates with corresponding confidence/credible intervals; treatment rankings may also be discussed. Authors may choose to summarize pairwise comparisons against a chosen treatment included in their analyses for brevity.  Discussion/Conclusions: limitations; conclusions and implications of findings.  Other: primary source of funding; systematic review registration number with registry name. | p. 1-2 |
|  |  |  |  |
| **INTRODUCTION** |  |  |  |
| Rationale | 3 | Describe the rationale for the review in the context of what is already known, including mention of why a network meta-analysis has been conducted. | p. 2-3 |
| Objectives | 4 | Provide an explicit statement of questions being addressed, with reference to participants, interventions, comparisons, outcomes, and study design (PICOS). | p. 3 |
|  |  |  |  |
| **METHODS** |  |  |  |
| Protocol and registration | 5 | Indicate whether a review protocol exists and if and where it can be accessed (e.g., Web address); and, if available, provide registration information, including registration number. | p. 4 |
| Eligibility criteria | 6 | Specify study characteristics (e.g., PICOS, length of follow-up) and report characteristics (e.g., years considered, language, publication status) used as criteria for eligibility, giving rationale. Clearly describe eligible treatments included in the treatment network, and note whether any have been clustered or merged into the same node (with justification). | p. 4 |
| Information sources | 7 | Describe all information sources (e.g., databases with dates of coverage, contact with study authors to identify additional studies) in the search and date last searched. | p. 4 |
| Search | 8 | Present full electronic search strategy for at least one database, including any limits used, such that it could be repeated. | p. 4;  Appendix 2 |
| Study selection | 9 | State the process for selecting studies (i.e., screening, eligibility, included in systematic review, and, if applicable, included in the meta-analysis). | p. 4, 6;  Fig. 1;  p. 17 |
| Data collection process | 10 | Describe method of data extraction from reports (e.g., piloted forms, independently, in duplicate) and any processes for obtaining and confirming data from investigators. | p. 4 |
| Data items | 11 | List and define all variables for which data were sought (e.g., PICOS, funding sources) and any assumptions and simplifications made. | p. 4-6; Appendix 3-4 |
| Geometry of the network | S1 | Describe methods used to explore the geometry of the treatment network under study and potential biases related to it. This should include how the evidence base has been graphically summarized for presentation, and what characteristics were compiled and used to describe the evidence base to readers. | p. 5;  Figures 2,4;  p. 17-18; Appendix 7 |
| Risk of bias within individual studies | 12 | Describe methods used for assessing risk of bias of individual studies (including specification of whether this was done at the study or outcome level), and how this information is to be used in any data synthesis. | p. 5 |
| Summary measures | 13 | State the principal summary measures (e.g., risk ratio, difference in means). Also describe the use of additional summary measures assessed, such as treatment rankings and surface under the cumulative ranking curve (SUCRA) values, as well as modified approaches used to present summary findings from meta-analyses. | p. 5 |
| Planned methods of analysis | 14 | Describe the methods of handling data and combining results of studies for each network meta-analysis. This should include, but not be limited to:  Handling of multi-arm trials;  Selection of variance structure;  Selection of prior distributions in Bayesian analyses; and  Assessment of model fit. | p. 5 |
| Assessment of Inconsistency | S2 | Describe the statistical methods used to evaluate the agreement of direct and indirect evidence in the treatment network(s) studied. Describe efforts taken to address its presence when found. | p. 5;  Appendix 6 |
| Risk of bias across studies | 15 | Specify any assessment of risk of bias that may affect the cumulative evidence (e.g., publication bias, selective reporting within studies). | p. 5;  Appendix 11 |
| Additional analyses | 16 | Describe methods of additional analyses if done, indicating which were pre-specified. This may include, but not be limited to, the following:  Sensitivity or subgroup analyses;  Meta-regression analyses;  Alternative formulations of the treatment network; and  Use of alternative prior distributions for Bayesian analyses (if applicable). | p. 5;  P. 8-9; Appendix 13-15 |
|  |  |  |  |
| **RESULTS** |  |  |  |
| Study selection | 17 | Give numbers of studies screened, assessed for eligibility, and included in the review, with reasons for exclusions at each stage, ideally with a flow diagram. | p. 6;  Fig. 1;  p. 17 |
| Presentation of network structure | S3 | Provide a network graph of the included studies to enable visualization of the geometry of the treatment network. | p. 7-8;  Figures 2,4;  p. 17-18; Appendix 7 |
| Summary of network geometry | S4 | Provide a brief overview of characteristics of the treatment network. This may include commentary on the abundance of trials and randomized patients for the different interventions and pairwise comparisons in the network, gaps of evidence in the treatment network, and potential biases reflected by the network structure. | p. 6-8; Appendix 7 |
| Study characteristics | 18 | For each study, present characteristics for which data were extracted (e.g., study size, PICOS, follow-up period) and provide the citations. | p. 6;  Appendix 3-4 |
| Risk of bias within studies | 19 | Present data on risk of bias of each study and, if available, any outcome level assessment. | p. 6;  Appendix 5 |
| Results of individual studies | 20 | For all outcomes considered (benefits or harms), present, for each study: 1) simple summary data for each intervention group, and 2) effect estimates and confidence intervals. Modified approaches may be needed to deal with information from larger networks. | p. 7-8; Appendix 9,12,16 |
| Synthesis of results | 21 | Present results of each meta-analysis done, including confidence/credible intervals. In larger networks, authors may focus on comparisons versus a particular comparator (e.g. placebo or standard care), with full findings presented in an appendix. League tables and forest plots may be considered to summarize pairwise comparisons. If additional summary measures were explored (such as treatment rankings), these should also be presented. | p. 6-8;  Figures 3,5;  p. 18-19; Appendix 8-9,16 |
| Exploration for inconsistency | S5 | Describe results from investigations of inconsistency. This may include such information as measures of model fit to compare consistency and inconsistency models, P values from statistical tests, or summary of inconsistency estimates from different parts of the treatment network. | p. 6;  Appendix 6 |
| Risk of bias across studies | 22 | Present results of any assessment of risk of bias across studies for the evidence base being studied. | p. 6;  Appendix 11 |
| Results of additional analyses | 23 | Give results of additional analyses, if done (e.g., sensitivity or subgroup analyses, meta-regression analyses, alternative network geometries studied, alternative choice of prior distributions for Bayesian analyses, and so forth). | p. 8-9; Appendix 13-15 |
|  |  |  |  |
| **DISCUSSION** |  |  |  |
| Summary of evidence | 24 | Summarize the main findings, including the strength of evidence for each main outcome; consider their relevance to key groups (e.g., healthcare providers, users, and policy-makers). | p. 9-11 |
| Limitations | 25 | Discuss limitations at study and outcome level (e.g., risk of bias), and at review level (e.g., incomplete retrieval of identified research, reporting bias). Comment on the validity of the assumptions, such as transitivity and consistency. Comment on any concerns regarding network geometry (e.g., avoidance of certain comparisons). | p. 11 |
| Conclusions | 26 | Provide a general interpretation of the results in the context of other evidence, and implications for future research. | p. 11 |
|  |  |  |  |
| **FUNDING** |  |  |  |
| Funding | 27 | Describe sources of funding for the systematic review and other support (e.g., supply of data); role of funders for the systematic review. This should also include information regarding whether funding has been received from manufacturers of treatments in the network and/or whether some of the authors are content experts with professional conflicts of interest that could affect use of treatments in the network. | p. 12 |

**Appendix 2: Search strategy**

Table S1. Search strategy of PubMed

| # | *Searches* |
| --- | --- |
| 1 | (((("Carcinoma, Hepatocellular"[MeSH Terms]) OR ("Liver Neoplasms"[MeSH Terms])) OR (hepatocellular carcinoma[Title/Abstract])) OR (HCC[Title/Abstract]) OR (hepatoma[Title/Abstract]) OR (liver cancer[Title/Abstract]) OR (liver tumor[Title/Abstract]) OR (liver tumour[Title/Abstract]) OR (hepatic cancer[Title/Abstract]) OR (hepatic tumor[Title/Abstract]) OR (hepatic tumour[Title/Abstract])) |
| 2 | (((("Chemoembolization, Therapeutic"[MeSH Terms]) OR ("Embolization, Therapeutic"[MeSH Terms])) OR (transarterial chemoembolization[Title/Abstract]) OR (transarterial chemo-embolization[Title/Abstract]) OR (transcatheter arterial chemoembolization[Title/Abstract]) OR (transcatheter arterial chemo-embolization[Title/Abstract]) OR (TACE[Title/Abstract]) OR (cTACE[Title/Abstract]) OR (c-TACE[Title/Abstract]) OR (conventional TACE[Title/Abstract]) OR (lipiodol TACE[Title/Abstract]) OR (drug-eluting bead TACE[Title/Abstract]) OR (drug eluting bead TACE[Title/Abstract]) OR (DEB-TACE[Title/Abstract]) OR (DEB TACE[Title/Abstract]) OR (drug-eluting microsphere TACE[Title/Abstract]) OR (drug eluting microsphere TACE[Title/Abstract]) OR (DEM-TACE[Title/Abstract]) OR (DEM TACE[Title/Abstract]) OR (degradable starch microsphere TACE[Title/Abstract]) OR (DSM-TACE[Title/Abstract]) OR (DSM TACE[Title/Abstract]) OR (balloon-occluded TACE[Title/Abstract]) OR (balloon occluded TACE[Title/Abstract]) OR (B-TACE[Title/Abstract]) OR (BTACE[Title/Abstract]) OR (transarterial embolization[Title/Abstract]) OR (transcatheter arterial embolization[Title/Abstract]) OR (TAE[Title/Abstract]) OR (bland embolization[Title/Abstract]) OR (radioembolization[Title/Abstract]) OR (transarterial radioembolization[Title/Abstract]) OR (TARE[Title/Abstract]) OR (yttrium-90[Title/Abstract]) OR (yttrium 90[Title/Abstract]) OR (Y-90[Title/Abstract]) OR (Y90[Title/Abstract]) OR (selective internal radiation therapy[Title/Abstract]) OR (SIRT[Title/Abstract])) |
| 3 | (((randomized controlled trial[Publication Type]) OR (controlled clinical trial[Publication Type])) OR (randomized[Title/Abstract]) OR (randomised[Title/Abstract]) OR (randomly[Title/Abstract]) OR (random allocation[MeSH Terms]) OR (clinical trial[Publication Type]) OR (trial[Title/Abstract]) OR (placebo[Title/Abstract])) |
| 4 | #1 AND #2 AND #3 |

Table S2. Search strategy of Embase

| # | *Searches* |
| --- | --- |
| 1 | ('hepatocellular carcinoma'/exp OR 'liver tumor'/exp OR 'liver cancer'/exp OR hepatocellular carcinoma:ti,ab,kw OR HCC:ti,ab,kw OR hepatoma:ti,ab,kw OR liver cancer:ti,ab,kw OR liver tumor:ti,ab,kw OR liver tumour:ti,ab,kw OR hepatic cancer:ti,ab,kw OR hepatic tumor:ti,ab,kw OR hepatic tumour:ti,ab,kw) |
| 2 | ('transarterial chemoembolization'/exp OR 'chemoembolization'/exp OR 'embolization'/exp OR 'radioembolization'/exp OR transarterial chemoembolization:ti,ab,kw OR transarterial chemo-embolization:ti,ab,kw OR transcatheter arterial chemoembolization:ti,ab,kw OR transcatheter arterial chemo-embolization:ti,ab,kw OR TACE:ti,ab,kw OR cTACE:ti,ab,kw OR c-TACE:ti,ab,kw OR conventional TACE:ti,ab,kw OR lipiodol TACE:ti,ab,kw OR drug-eluting bead TACE:ti,ab,kw OR drug eluting bead TACE:ti,ab,kw OR DEB-TACE:ti,ab,kw OR DEB TACE:ti,ab,kw OR drug-eluting microsphere TACE:ti,ab,kw OR DEM-TACE:ti,ab,kw OR DEM TACE:ti,ab,kw OR degradable starch microsphere TACE:ti,ab,kw OR DSM-TACE:ti,ab,kw OR DSM TACE:ti,ab,kw OR balloon-occluded TACE:ti,ab,kw OR balloon occluded TACE:ti,ab,kw OR B-TACE:ti,ab,kw OR BTACE:ti,ab,kw OR transarterial embolization:ti,ab,kw OR transcatheter arterial embolization:ti,ab,kw OR TAE:ti,ab,kw OR bland embolization:ti,ab,kw OR radioembolization:ti,ab,kw OR transarterial radioembolization:ti,ab,kw OR TARE:ti,ab,kw OR yttrium-90:ti,ab,kw OR yttrium 90:ti,ab,kw OR Y-90:ti,ab,kw OR Y90:ti,ab,kw OR selective internal radiation therapy:ti,ab,kw OR SIRT:ti,ab,kw) |
| 3 | ('randomized controlled trial'/exp OR 'controlled clinical trial'/exp OR random:ti,ab,kw OR randomized:ti,ab,kw OR randomised:ti,ab,kw OR randomly:ti,ab,kw OR placebo:ti,ab,kw OR trial:ti,ab,kw |
| 4 | #1 AND #2 AND #3 |

Table S3. Search strategy of Web of Science

| # | *Searches* |
| --- | --- |
| 1 | TS=("hepatocellular carcinoma" OR HCC OR hepatoma OR "liver cancer" OR "liver tumor" OR "liver tumour" OR "hepatic cancer" OR "hepatic tumor" OR "hepatic tumour") |
| 2 | TS=("transarterial chemoembolization" OR "transarterial chemo-embolization" OR "transcatheter arterial chemoembolization" OR "transcatheter arterial chemo-embolization" OR TACE OR cTACE OR "c-TACE" OR "conventional TACE" OR "lipiodol TACE" OR "drug-eluting bead TACE" OR "drug eluting bead TACE" OR "DEB-TACE" OR "DEB TACE" OR "drug-eluting microsphere TACE" OR "DEM-TACE" OR "DEM TACE" OR "degradable starch microsphere TACE" OR "DSM-TACE" OR "DSM TACE" OR "balloon-occluded TACE" OR "balloon occluded TACE" OR "B-TACE" OR BTACE OR "transarterial embolization" OR "transcatheter arterial embolization" OR TAE OR "bland embolization" OR radioembolization OR "transarterial radioembolization" OR TARE OR "yttrium-90" OR "yttrium 90" OR "Y-90" OR Y90 OR "selective internal radiation therapy" OR SIRT) |
| 3 | TS=(random* OR randomized OR randomised OR randomly OR placebo OR trial OR "controlled trial" OR "randomized controlled trial" OR "randomised controlled trial") |
| 4 | #1 AND #2 AND #3 |

Table S4. Search strategy of Cochrane Central Register of Controlled Trials

(CENTRAL)

| # | *Searches* |
| --- | --- |
| 1 | MeSH descriptor: [Carcinoma, Hepatocellular] explode all trees |
| 2 | MeSH descriptor: [Liver Neoplasms] explode all trees |
| 3 | ("hepatocellular carcinoma" OR HCC OR hepatoma OR "liver cancer" OR "liver tumor" OR "liver tumour" OR "hepatic cancer" OR "hepatic tumor" OR "hepatic tumour"):ti,ab,kw |
| 4 | #1 OR #2 OR #3 |
| 5 | MeSH descriptor: [Chemoembolization, Therapeutic] explode all trees |
| 6 | MeSH descriptor: [Embolization, Therapeutic] explode all trees |
| 7 | ("transarterial chemoembolization" OR "transarterial chemo-embolization" OR "transcatheter arterial chemoembolization" OR "transcatheter arterial chemo-embolization" OR TACE OR cTACE OR "c-TACE" OR "conventional TACE" OR "lipiodol TACE" OR "drug-eluting bead TACE" OR "drug eluting bead TACE" OR "DEB-TACE" OR "DEB TACE" OR "drug-eluting microsphere TACE" OR "DEM-TACE" OR "DEM TACE" OR "degradable starch microsphere TACE" OR "DSM-TACE" OR "DSM TACE" OR "balloon-occluded TACE" OR "balloon occluded TACE" OR "B-TACE" OR BTACE OR "transarterial embolization" OR "transcatheter arterial embolization" OR TAE OR "bland embolization" OR radioembolization OR "transarterial radioembolization" OR TARE OR "yttrium-90" OR "yttrium 90" OR "Y-90" OR Y90 OR "selective internal radiation therapy" OR SIRT):ti,ab,kw |
| 8 | #5 OR #6 OR #7 |
| 9 | (random* OR randomized OR randomised OR randomly OR placebo* OR trial OR "controlled trial" OR "randomized controlled trial" OR "randomised controlled trial"):ti,ab,kw |
| 10 | #4 AND #8 AND #9 |

Table S5. Search strategy of CNKI

| # | *Searches* |
| --- | --- |
| 1 | SU=(hepatocellular carcinoma OR liver cancer OR primary liver cancer OR hepatocellular liver cancer OR HCC OR hepatoma) |
| 2 | SU=(transarterial chemoembolization OR transcatheter arterial chemoembolization OR hepatic arterial chemoembolization OR TACE OR cTACE OR c-TACE OR conventional TACE OR traditional TACE OR lipiodol TACE OR drug-eluting microspheres OR drug-eluting beads OR drug-loaded microspheres OR drug-loaded embolic microspheres OR DEB-TACE OR DEM-TACE OR degradable starch microspheres OR DSM-TACE OR balloon-occluded TACE OR balloon-occlusion TACE OR B-TACE OR BTACE OR transarterial embolization OR transcatheter arterial embolization OR bland embolization OR TAE OR radioembolization OR transarterial radioembolization OR TARE OR yttrium-90 OR Y90 OR Y-90 OR selective internal radiation therapy OR SIRT) |
| 3 | SU=(randomized OR randomised OR random allocation OR random grouping OR randomized controlled trial OR controlled trial OR clinical trial OR RCT) |
| 4 | #1 AND #2 AND #3 |

**Appendix 3: Characteristics of included studies**

**Table S3.1: Baseline of characteristics of included studies**

| **First author (Year)** | **Country and region** | **Design** | **Intervention measures (Test/Control)** | **Patients, N (%)** | **Sex (male/female), N (%)** | **Age (mean ± SD), years** | **HBV, N (%)** | **BCLC (A/B/C), N (%)** | **Child-Pugh (A/B), N (%)** | **ECOG performance status (0/1/2), N (%)** | **AFP (ng/ml), N (%)** | **Maximum tumor diameter (cm)** | **Tumor number (Single/Multiple), N (%)** | **Follow-up (months)** |
| --- | --- | --- | --- | --- | --- | --- | --- | --- | --- | --- | --- | --- | --- | --- |
| van Malenstein (2011)^1^ | Belgium | RCT | DEB-TACE vs cTACE | 16 / 14 | 14 / 2  11 / 3 | 67.3 ± 9.8 / 56.6 ± 13.4 | 4 / 4 | 2/9/5  1/10/3 | 14 / 2  14 / 0 | 9/7/0  10/2/2 | <13.6/13.7–300/>300：  5/3/8  7/3/4 | NA | NA | 1.5 |
| Shi Z X (2023)^2^ | China | RCT | DEB-TACE vs cTACE | 45 / 45 | 40 / 5  39 / 6 | 58.9 ± 7.1 / 60.6 ± 6.5 | 31 / 27 | 17/28/0  14/31/0 | 33 / 12  29 / 16 | 17/28/0  13/32/0 | NA | NA | 39 / 6  34 / 11 | 12 |
| Shan L T (2026)^3^ | China | RCT | B-TACE vs cTACE | 30 / 30 | 24 / 6 25 / 5 | 61.0 ± 15.9 / 56.5 ± 13.3 | 13 / 13 | 1/7/22 0/15/15 | 28 / 2 25 / 5 | 18/11/1 20/10/0 | ≤200/>200： 10 / 21  20 / 9 | 9.1 (6.4, 13.3) / 8.8 (6.8, 12.0) | 22 / 8 14 / 16 | 6 |
| Jin B X (2024)^4^ | China | RCT | DEB-TACE vs cTACE | 72 / 72 | 48 / 24  49 / 23 | 56.3 ± 9.2 / 57.1 ± 8.7 | NA | NA | NA | NA | 612.4 ± 234.1 / 628.7 ± 241.3 | NA | NA | 12 |
| T.J.Vogl (2021)^5^ | Germany | RCT | DSM-TACE vs cTACE | 31 / 30 | 22 / 9 22 / 8 | 70.5 ± 9.5 / 72.0 ± 10.3 | 5 / 6 | 11/11/9 5/22/3 | 21 / 10 24 / 6 | NA | 18.2 (1.9–58,883) / 22.1 (1.5–5900) | 38.66 ± 48.30 / 49.54 ± 35.34 | 15 / 16 9 / 21 | NA |
| Johannes Lammer (2010)^6^ | Vienna | RCT | DEB-TACE vs cTACE | 93 / 108 | 79 / 14  95 / 13 | 67.3 ± 9.1 / 67.4 ± 8.8 | 16 / 18 | 24/69/0  29/79/0 | 77 / 16  89 / 19 | 74/19/0  80/28/0 | NA | NA | 39 / 54 40 / 68 | NA |
| R Golfieri (2014)^7^ | Italy | RCT | DEB-TACE vs cTACE | 89 / 88 | 66 / 22 69 / 17 | 68.9 ± 8.0 / 68.3 ± 8.0 | 62 / 68 | 41/26/22 41/23/24 | 75 / 14 77 / 11 | 64/25/0 67/21/0 | NA | NA | 45 / 44 39 / 49 | 24 |
| Aleksandar Gjoreski (2020)^8^ | North Macedonia | RCT | DSM-TACE vs cTACE | 32 / 28 | 21 / 11 20 / 8 | 68.81 ± 7.0 / 67.93 ± 7.4 | 15 / 18 | NA | 9 / 21 15 / 12 | 21/11/0 18/10/0 | ≤150/>150：  25 / 7 25 / 3 | 6.23 ± 2.6 / 5.82 ± 2.4 | 19 / 13 18 / 10 | 24 |
| Duan X H (2024)^9^ | China | RCT | DEB-TACE vs cTACE | 102 / 105 | 87 / 15 87 / 18 | 56.79 ± 10.56 / 56.70 ± 9.11 | 76 / 70 | 0/21/81 0/14/91 | 42 / 60 49 / 56 | 39/63/0 33/72/0 | ≤400/>400： 53 / 49 61 / 44 | 9.50 ± 3.67 / 9.03 ± 3.57 | 49 / 53 40 / 65 | 23 |
| Li K F (2023)^10^ | China | RCT | DEB-TACE vs cTACE | 52 / 52 | 27 / 25 31 / 21 | 56.1 ± 7.3 / 54.6 ± 6.9 | NA | NA | 41 / 11 38 / 14 | 32/20/0 28/24/0 | 560.3 ± 41.8 / 576.4 ± 49.2 | 7.20 ± 1.30 / 6.90 ± 1.10 | 5 / 47 10 / 42 | 12 |
| Shi J (2022)^11^ | China | RCT | DEB-TACE vs cTACE | 50 / 50 | 42 / 8 38 / 12 | 66.08 ± 12.16 / 61.62 ± 12.64 | NA | 0/37/13 0/35/15 | 30 / 20 28 / 22 | NA | 15.53 ± 2.58 / 15.76 ± 2.69 | 5.67 ± 1.90 / 5.60 ± 1.83 | NA | 36 |
| Chen P (2021)^12^ | China | RCT | DEB-TACE vs cTACE | 40 / 40 | 25 / 15 22 / 18 | 56.81 ± 6.90 / 55.74 ± 7.35 | NA | NA | 18 / 22 15 / 25 | NA | 188.62 ± 30.49 / 185.20 ± 32.07 | NA | 36 / 4 34 / 6 | 12 |
| Chen J X (2025)^13^ | China | RCT | DEB-TACE vs cTACE | 40 / 40 | 38 / 2 35 / 5 | 55.03 ± 12.84 / 52.88 ± 10.26 | NA | NA | 22 / 18 24 / 16 | NA | 175.73 ± 36.26 / 187.29 ± 40.82 | 4.92 ± 1.64 / 4.45 ± 1.22 | NA | 2 |
| Huang Z (2020)^14^ | China | RCT | DEB-TACE vs cTACE | 60 / 60 | 35 / 25 33 / 17 | 58.5 ± 2.3 / 59.1 ± 2.4 | NA | NA | NA | NA | NA | NA | NA | 1.5 |
| Zhang D Y (2021)^15^ | China | RCT | DEB-TACE vs cTACE | 36 / 36 | 24 / 12 23 / 13 | 57.26 ± 9.17 / 57.02 ± 9.04 | NA | NA | 27 / 9 26 / 10 | NA | NA | 6.26 ± 3.27 / 6.14 ± 3.22 | 9 / 27 10 / 26 | 6 |
| Zhang C (2024)^16^ | China | RCT | DEB-TACE vs cTACE | 46 / 46 | 31 / 15 33 / 13 | 52.46 ± 4.87 / 51.26 ± 5.24 | NA | NA | NA | NA | NA | 4.96 ± 0.82 / 5.03 ± 0.79 | NA | 2 |
| Chi Q (2018)^17^ | China | RCT | DEB-TACE vs cTACE | 50 / 50 | 35 / 15 40 / 10 | 57.5 ± 10.7 / 57.2 ± 10.6 | NA | NA | 35 /15 40 /10 | NA | 4138.79 ± 472.86 / 4365.79 ± 410.23 | 4.62 ± 0.80 / 4.54 ± 0.76 | 10 / 40 6 / 44 | 1 |
| Wang H P (2018)^18^ | China | RCT | DEB-TACE vs cTACE | 60 / 69 | 42 / 18  48 / 21 | 61.7 ± 9.8 / 59.4 ± 11.2 | 44 / 46 | NA | 56 / 4  62 / 6 | NA | 27.43 ± 171.08 / 100.44 ± 546.05 | 4.20 ± 2.80 / 5.00 ± 3.10 | NA | 3 |
| Wang Z (2019)^19^ | China | RCT | DEB-TACE vs cTACE | 45 / 45 | 21 / 24 23 / 22 | 66.17 ± 12.86 / 65.72 ± 11.69 | NA | NA | 28 / 17 26 / 19 | NA | NA | 5.43 ± 1.68 / 5.46 ± 1.74 | 35 / 10 33 / 12 | 24 |
| Zeng Q S (2024)^20^ | China | RCT | DEB-TACE vs cTACE | 48 / 48 | 28 / 20 27 / 21 | 57.36 ± 6.08 / 56.24 ± 5.15 | NA | NA | 19 / 29 21 / 27 | NA | 186.07 ± 31.29 / 184.56 ± 30.34 | NA | 41 / 7 43 / 5 | 3 |
| Zhang N (2022)^21^ | China | RCT | DEB-TACE vs cTACE | 30 / 30 | 20 / 10 19 / 11 | 50.98 ± 10.21 / 51.23 ± 9.34 | NA | NA | NA | NA | NA | 5.11 ± 0.99 / 4.89 ± 1.23 | NA | 2 |
| Chen Y (2017)^22^ | China | RCT | DEB-TACE vs cTACE | 46 / 46 | 29 / 17 26 / 20 | 50.35 ± 8.43 / 51.37 ± 9.01 | NA | 0/30/16 0/28/18 | 21 / 25 19 / 27 | NA | 1115.21 ± 384.90 / 1239.48 ± 405.26 | 5.31 ± 2.48 / 5.70 ± 2.16 | NA | 24 |
| Lu Y (2023)^23^ | China | RCT | DEB-TACE vs cTACE | 35 / 35 | 19 / 16 19 / 16 | 51.20 ± 2.23 / 51.02 ± 2.16 | NA | NA | 22 / 13 21 / 14 | NA | NA | NA | NA | 12 |
| Geng J Z(2022)^24^ | China | RCT | DEB-TACE vs cTACE | 29 / 29 | 18 / 11 19 / 10 | 63.16 ± 5.29 / 63.39 ± 5.14 | NA | 0/15/14 0/14/15 | 14 / 15 13 / 16 | NA | NA | NA | 15 / 14 13 / 16 | 1 |
| Li Y F(2020)^25^ | China | RCT | DEB-TACE vs cTACE | 38 / 38 | 29 / 9 25 / 13 | 51.9 ± 9.6 / 51.6 ± 10.1 | NA | NA | 17 / 21 12 / 26 | NA | NA | 4.70 ± 1.20 / 5.10 ± 1.20 | NA | 12 |
| Wang X L (2023)^26^ | China | RCT | DEB-TACE vs cTACE | 32 / 32 | 22 / 10 23 / 9 | 57.65 ± 4.52 / 56.87 ± 4.94 | NA | NA | 18 / 14 16 / 16 | NA | NA | 5.72 ± 1.15 / 5.58 ± 1.09 | NA | 2 |
| Zhang J (2022)^27^ | China | RCT | DEB-TACE vs cTACE | 46 / 46 | 27 / 19 26 / 20 | 67.53 ± 11.37 / 67.63 ± 11.24 | NA | NA | NA | NA | 468.57 ± 35.41 / 468.78 ± 35.78 | NA | NA | 1 |
| Zhao P (2022)^28^ | China | RCT | DEB-TACE vs cTACE | 40 / 40 | 22 / 18 24 / 16 | 60.21 ± 2.14 / 60.15 ± 2.08 | NA | NA | 23 / 17 25 / 15 | NA | NA | NA | NA | 3 |
| Xu R L (2023)^29^ | China | RCT | DEB-TACE vs cTACE | 53 / 53 | 29 / 24 32 / 21 | 54.37 ± 8.69 / 55.08 ± 8.85 | NA | 12/19/22 10/17/26 | 25/28 22/31 | NA | 728.65 ± 32.15 / 734.18 ± 34.86 | 5.49 ± 0.82 / 5.58 ± 0.86 | 18 / 35 16 / 37 | 12 |
| Zhang M X (2022)^30^ | China | RCT | DEB-TACE vs cTACE | 48 / 48 | 40 / 8 41 / 7 | 55.12 ± 2.52 / 55.33 ± 2.65 | NA | NA | NA | NA | NA | NA | NA | 6 |
| Zhou L J (2023)^31^ | China | RCT | DEB-TACE vs cTACE | 40 / 40 | 21 / 19 22 / 18 | 65.21 ± 7.03 / 65.00 ± 7.14 | NA | NA | 29 / 11 24 / 16 | NA | NA | NA | NA | 24 |
| Li X D (2025)^32^ | China | RCT | DEB-TACE vs cTACE | 30 / 30 | 18 / 12 17 / 13 | 56.12 ± 2.50 / 55.36 ± 3.00 | NA | NA | 18 / 12 19 / 11 | NA | 465.15 ± 10.26 / 462.82 ± 9.75 | 4.21 ± 1.94 / 4.45 ± 2.16 | NA | 3 |
| Dai S B (2022)^33^ | China | RCT | DEB-TACE vs cTACE | 67 / 67 | 41 / 26 40 / 27 | 58.51 ± 4.19 / 58.50 ± 4.15 | NA | NA | 61 / 6 59 / 8 | NA | NA | NA | 45 / 22 47 / 20 | 6 |
| Pang H C (2022)^34^ | China | RCT | DEB-TACE vs cTACE | 30 / 30 | 22 / 8 11 / 19 | 54.74 ± 4.57 / 54.27 ± 4.61 | NA | NA | 23 / 7 24 / 6 | NA | NA | 13.16 ± 4.71 / 13.45 ± 4.12 | NA | 6 |
| Li H B (2025)^35^ | China | RCT | DEB-TACE vs cTACE | 61 / 61 | 48 / 13 50 / 11 | 59.00 ± 8.10 / 58.00 ± 7.90 | 53 / 52 | 15/30/16 17/33/11 | 24 / 37 28 / 33 | NA | 1492.11 ± 229.97 / 1457.49 ± 229.08 | 5.44 ± 3.02 / 5.52 ± 2.88 | 21 / 40 19 / 42 | 3 |
| Peng C Y (2020)^36^ | China | RCT | DEB-TACE vs cTACE | 60 / 60 | 22 / 8 23 / 7 | 53.50 ± 13.50 / 52.87 ± 13.56 | NA | 0/48/12 0/45/15 | 34 / 26 36 / 24 | NA | NA | 5.42 ± 2.36 / 5.32 ± 1.81 | NA | 12 |
| Jiang F S (2021)^37^ | China | RCT | DEB-TACE vs cTACE | 30 / 30 | 22 / 8 21 / 9 | 57.32 ± 3.25 / 58.11 ± 3.32 | NA | 0/9/21 0/10/20 | 24 / 6 23 / 7 | NA | NA | 12.89 ± 2.08 / 12.73 ± 2.12 | NA | 18 |
| Zhao L (2020)^38^ | China | RCT | DEB-TACE vs cTACE | 30 / 30 | 20 / 10 19 / 11 | 49.5 ± 6.6 / 49.8 ± 6.4 | NA | NA | NA | NA | NA | 4.10 ± 1.80 / 4.30 ± 1.70 | 13 / 17 12 / 18 | 3 |
| Zhao W (2020)^39^ | China | RCT | DEB-TACE vs cTACE | 28 / 28 | 21 / 7 19 / 9 | 57.48 ± 3.56 / 57.69 ± 3.47 | NA | NA | NA | NA | NA | NA | NA | 12 |
| Josep M Llovet (2002)^40^ | Spain | RCT | TAE vs cTACE | 37 / 40 | 30 / 7 32 / 8 | 64.00 ± 3.70 / 63.00 ± 3.70 | 2 / 4 | 0/28/9 0/35/5 | 27 / 10 31 / 9 | 28/7/2 35/4/1 | ＜10/10–100/＞100： 15/9/13 15/18/7 | 5.20 ± 1.00 / 4.90 ± 1.30 | 9 / 27 13 / 26 | 51 |
| T Meyer (2013)^41^ | British | RCT | DEB-TACE vs TAE | 44 / 42 | 39 / 5 35 / 7 | 63.20 ± 8.80 / 62.60 ± 13.63 | 16 / 15 | 11/18/12 9/16/15 | 38 / 6 33 / 9 | 31/8/5 27/9/6 | ≤400/>400： 33 / 11 32 / 10 | 5.11 ± 2.59 / 6.33 ± 3.34 | 15 / 29 13 / 29 | 24 |
| Riad Salem (2017)^42^ | America | RCT | Y90（TARE） vs cTACE | 24 / 21 | 17 / 7 16 / 5 | 64.00 ± 5.93 / 62.00 ± 5.19 | 3 / 2 | 18/6/0 17/4/0 | 12 / 12 15 / 6 | NA | <200/≥200： 21 / 3 19 / 2 | NA | 13 / 11 11 / 10 | 17.2 |
| Frank T. Kolligs (2014)^43^ | Germany | RCT | Y90（TARE） vs cTACE | 13 / 15 | 11 / 2 13 / 2 | 65.80 ± 6.73 / 66.70 ± 9.01 | NA | 5/5/3 4/8/3 | 12 / 1 13 / 2 | 10/3/0 12/3/0 | 660.00 ± 2178.00 / 660.00 ± 2178.00 | NA | NA | 10.7 |
| Saburo Kawail (1992)^44^ | Japan | RCT | TAE vs cTACE | 141 / 148 | 118 / 21 125 / 22 | 62.00 ± 10.50 / 61.00 ± 11.00 | NA | NA | 102 / 25 107 / 33 | 77/36/3 71/38/10 | 2,435 / 3,177 | NA | NA | NA |
| Katerina Malagari (2010)^45^ | Greece | RCT | DEB-TACE vs TAE | 41 / 43 | NA | 70.70 ± 6.90 / 70.00 ± 7.90 | NA | NA | 23 / 18 26 / 17 | 26/15/0 28/15/0 | ＞400 | 8.35 ± 2.75 / 8.10 ± 2.80 | 12 / 29 15 / 28 | 12 |
| Karen T. Brown (2016)^46^ | America | RCT | DEB-TACE vs TAE | 50 / 51 | 41 / 9 37 / 14 | 65.52 ± 11.82 / 68.33 ± 9.72 | 7 / 8 | 12/23/15 10/22/19 | 45 / 5 41 / 10 | 43/7/0 44/7/0 | NA | 4.3 ± 3.1 / 4.7 ± 3.7 | 12 / 38 12 / 39 | 34 |
| Hironobu Ihira (2023)^47^ | Japan | RCT | TAE vs cTACE | 26 / 28 | 16 / 10 24 / 4 | 77.00 ± 8.00 / 71.00 ± 10.00 | 0 / 3 | NA | 24 / 4 22 / 6 | NA | NA | NA | NA | 3 |
| Elisabeth Dhondt (2022)^48^ | Belgium | RCT | DEB-TACE vs Y90（TARE） | 34 / 38 | 30 / 4 33 / 5 | 68.00 ± 11.50 / 67.00 ± 8.50 | 5 / 5 | 4 / 30 7 / 31 | 29 / 5 36 / 2 | 29 / 5 34 / 4 | <400/≥400： 28 / 5 33 / 4 | NA | 4 / 30 8 / 30 | 6 |
| Jean-Luc Raoul (1997)^49^ | France | RCT | I131（TARE） vs cTACE | 65 / 64 | 62 / 3  60 / 4 | 64.60 ± 7.00 / 65.70 ± 6.00 | 10 / 8 | NA | 53 / 11 44 / 19 | NA | 1992 ± 6510 / 1738 ± 5310 | NA | 33 / 32 31 / 33 | 48 |
| Yu C H (2014)^50^ | China | RCT | TAE vs cTACE | 45 / 45 | 35 / 10 37 / 8 | 65.00 ± 15.00 / 65.00 ± 15.00 | 39 / 28 | 5/39/1 12/33/0 | 36 / 9 37 / 8 | 28/16/0 31/12/2 | <200/≥200： 26 / 19 31 / 14 | NA | 21 / 24 22 / 23 | 48 |
| Rodolfo Sacco (2011)^51^ | Italy | RCT | DEB-TACE vs cTACE | 33 / 34 | 23 / 10 22 / 12 | 71.3 ± 7.2 / 68.7 ± 8.1 | 4 / 4 | 22/11/0 22/12/0 | 29 / 4 25 / 9 | NA | 662 ± 1679 / 67.5 ± 202 | 4.47 ± 2.68 / 3.85 ± 1.89 | 25 / 8 28 / 6 | 43.5 |
| Michael B. Pitton (2014)^52^ | Germany | RCT | DEB-TACE vs Y90（TARE） | 12 / 12 | 10 / 2 8 / 4 | 70.5 ± 9.0 / 71.8 ± 7.2 | 0 / 1 | 1/11/0 0/12/0 | 9 / 3 10 / 2 | NA | 164 ± 529 / 3308 ± 10204 | 6.08 ± 3.70 / 6.13 ± 3.64 | 5 / 7 4 / 8 | 36 |
| Yang Y B (2012)^53^ | China | RCT | B-TACE vs cTACE | 40 / 40 | 36 / 4 35 / 5 | 52.5 ± 9.9 / 51.1 ± 11.7 | NA | NA | 37 / 3 39 / 1 | NA | NA | 8.60 ± 4.10 / 9.20 ± 4.00 | NA | 12 |
| Cheng H Y (2004)^54^ | China | RCT | B-TACE vs cTACE | 34 / 40 | NA | NA | NA | NA | NA | NA | NA | NA | NA | 10 |
| Ma M (2019)^55^ | China | RCT | TAE vs cTACE | 32 / 32 | 18 / 14 17 / 15 | 57.62 ± 5.75 / 57.62 ± 5.71 | NA | NA | 10 / 11 10 / 10 | NA | NA | NA | NA | 12 |
| Feng T (2020)^56^ | China | RCT | TAE vs cTACE | 40 / 40 | 21 / 19 22 / 18 | 65.51 ± 2.26 / 65.67 ± 2.12 | NA | NA | 19 / 21 20 / 20 | NA | NA | NA | NA | NA |

**References**

1. van Malenstein H, Maleux G, Vandecaveye V, et al. A randomized phase II study of drug-eluting beads versus transarterial chemoembolization for unresectable hepatocellular carcinoma. Onkologie. 2011;34(7):368-376. doi:10.1159/000329602.
2. Shi Z, Wang D, Kang T, Yi R, Cui L, Jiang H. Comparison of CalliSpheres® microspheres drug-eluting beads and conventional transarterial chemoembolization in hepatocellular carcinoma patients: a randomized controlled trial. Radiol Oncol. 2023;57(1):70-79. Published 2023 Feb 17. doi:10.2478/raon-2023-0001.
3. Shan L, Fan Z, Yang G, et al. Efficacy of B-TACE Versus C-TACE and Potential Predictive Value of Intraoperative Balloon-Occluded Stump Pressure in HCC. J Clin Med. 2026;15(2):668. Published 2026 Jan 14. doi:10.3390/jcm15020668.
4. Jin B, Gu Y, Xi S, et al. Efficacy of CalliSpheres® drug-loaded microspheres combined with doxorubicin in hepatocellular carcinoma. Scand J Gastroenterol. 2024;59(9):1087-1092. doi:10.1080/00365521.2024.2390025.
5. Vogl TJ, Langenbach MC, Hammerstingl R, Albrecht MH, Chatterjee AR, Gruber-Rouh T. Evaluation of two different transarterial chemoembolization protocols using Lipiodol and degradable starch microspheres in therapy of hepatocellular carcinoma: a prospective trial. Hepatol Int. 2021;15(3):685-694. doi:10.1007/s12072-021-10193-8.
6. Lammer J, Malagari K, Vogl T, et al. Prospective randomized study of doxorubicin-eluting-bead embolization in the treatment of hepatocellular carcinoma: results of the PRECISION V study. Cardiovasc Intervent Radiol. 2010;33(1):41-52. doi:10.1007/s00270-009-9711-7.
7. Golfieri R, Giampalma E, Renzulli M, et al. Randomised controlled trial of doxorubicin-eluting beads vs conventional chemoembolisation for hepatocellular carcinoma. Br J Cancer. 2014;111(2):255-264. doi:10.1038/bjc.2014.199.
8. Gjoreski A, Jovanoska I, Risteski F, et al. Single-center randomized trial comparing conventional chemoembolization versus doxorubicin-loaded polyethylene glycol microspheres for early- and intermediate-stage hepatocellular carcinoma. Eur J Cancer Prev. 2021;30(3):258-266. doi:10.1097/CEJ.0000000000000623.
9. Duan X, Li H, Chen P, et al. Transcatheter arterial chemoembolization using CalliSpheres beads loaded with arsenic trioxide for unresectable large or huge hepatocellular carcinoma: a prospective study. Eur Radiol. 2024;34(2):1258-1267. doi:10.1007/s00330-023-10097-1.
10. Li KF, Zheng R, Hu ZM. Efficacy of CalliSpheres drug-eluting beads loaded with epirubicin in the treatment of unresectable primary liver cancer. J Interv Radiol. 2023;32(2):131-135. Chinese.
11. Shi J, Xie J, Wang HJ, et al. Clinical study of CalliSpheres drug-loaded microspheres in transcatheter arterial chemoembolization for liver cancer. Modern Digestion & Intervention. 2022;27(12):1566-1569. Chinese.
12. Chen P, Tian SJ, Zhang HK, et al. Efficacy of CalliSpheres drug-eluting microsphere embolization in patients with primary massive hepatocellular carcinoma and its effects on tumor markers and liver function. Journal of Bengbu Medical College. 2021;46(4):512-517. doi:10.13898/j.cnki.issn.1000-2200.2021.04.022 . Chinese.
13. Chen JX, Li XY. Comparison of DSA-guided drug-eluting bead transarterial chemoembolization and conventional transarterial chemoembolization in patients with unresectable liver cancer. Journal of Imaging Research and Medical Applications. 2025;9(16):179-182. doi:10.20267/j.issn.2096-3807.2025.16.056 . Chinese.
14. Huang Z, Su GF, Zhang LB. Clinical effect of repeated arterial chemoembolization with Embosphere microspheres in patients with intermediate-advanced liver cancer. Internal Medicine. 2020;15(2):170-172,185. doi:10.16121/j.cnki.cn45-1347/r.2020.02.14 . Chinese.
15. Zhang DY. Application effect of HepaSphere drug-eluting microspheres in transcatheter arterial chemoembolization for unresectable liver cancer. Clinical Medicine. 2021;41(8):66-68. doi:10.19528/j.issn.1003-3548.2021.08.026 . Chinese.
16. Zhang C, Dai YH, Zhao T, et al. Clinical efficacy and adverse reactions of pirarubicin-loaded microspheres combined with lobaplatin in interventional embolization therapy for primary liver cancer. Anti-tumor Pharmacy. 2024;14(4):463-467. Chinese.
17. Chi Q, Zhang LL, Liu B, et al. Efficacy of epirubicin combined with lipiodol or HepaSphere drug-eluting microspheres for TACE in patients with liver cancer. Journal of Modern Oncology. 2018;26(13):2090-2093. Chinese.
18. Wang HP, Zhang LH, Li LK. Clinical efficacy of doxorubicin-loaded microsphere transcatheter arterial chemoembolization in the treatment of liver cancer. China Pharmacist. 2018;21(4):654-656. Chinese.
19. Wang Z, Liu QY, Yang W, et al. Clinical value of domestic CalliSpheres drug-eluting bead transarterial chemoembolization for unresectable liver cancer. Chinese Hepatology. 2019;24(7):767-770. doi:10.14000/j.cnki.issn.1008-1704.2019.07.014 . Chinese.
20. Zeng QS, Wei CN. Effect of interventional embolization with polyvinyl alcohol drug-loaded microspheres on liver function and alpha-fetoprotein concentration in patients with primary liver cancer. The Journal of Medical Theory and Practice. 2024;37(1):64-66. doi:10.19381/j.issn.1001-7585.2024.01.023 . Chinese.
21. Zhang N. Clinical study of interventional embolization with polyvinyl alcohol drug-loaded microspheres in patients with primary liver cancer. Modern Medicine and Health Research Electronic Journal. 2022;6(4):14-17. Chinese.
22. Chen Y, Wei XY, Cao K, et al. Efficacy of drug-eluting microsphere transarterial chemoembolization in the treatment of intermediate-advanced primary liver cancer. Chinese Journal of Clinical Oncology and Rehabilitation. 2017;24(6):711-714. Chinese. doi:10.13455/j.cnki.cjcor.2017.06.21
23. Lu Y, Zhang YZ, Zhao Q, et al. Effect of drug-loaded microsphere arterial infusion combined with TACE in the treatment of intermediate-advanced primary liver cancer and its influence on liver function and survival rate. Chinese and Foreign Medical Research. 2023;21(32):128-132. Chinese. doi:10.14033/j.cnki.cfmr.2023.32.032
24. Geng JZ, Yang XB. Clinical efficacy and safety of drug-loaded microsphere hepatic arterial chemoembolization for large liver cancer. Clinical Research and Practice. 2022;7(21):67-70. Chinese. doi:10.19347/j.cnki.2096-1413.202221019
25. Li FY, Sun YM, Shi MB. Comparative value of drug-loaded microspheres and super-liquefied lipiodol in hepatic arterial embolization for liver cancer. Modern Digestion & Intervention. 2020;25(5):649-652. Chinese.
26. Wang XL, Xiao B, Bai XD, et al. Efficacy of pirarubicin-loaded microsphere chemoembolization in patients with hepatocellular carcinoma and its effects on serum VEGF, GPC3, and AFP-L3 levels. Heilongjiang Medicine and Pharmacy. 2023;46(3):48-50. Chinese.
27. Zhang J, Huang YD, Mao J. Application value of interventional embolization with drug-loaded microspheres for primary liver cancer. World Journal of Complex Medicine. 2022;8(12):150-153. Chinese.
28. Zhao P. Clinical efficacy and safety of drug-loaded microsphere transcatheter arterial chemoembolization in the treatment of liver cancer. Jilin Medical Journal. 2022;43(3):627-629. Chinese.
29. Xu RL, Shen SB, Yu LW, et al. Comparison of the efficacy and safety of drug-loaded microsphere transarterial chemoembolization and conventional hepatic arterial chemoembolization combined with sorafenib in the treatment of intermediate-advanced hepatocellular carcinoma. Journal of Clinical Surgery. 2023;31(4):383-386. Chinese.
30. Zhang MX, Shen JY, Jiang MY. Efficacy analysis of drug-loaded microspheres combined with TACE in patients with primary liver cancer. Heilongjiang Medicine and Pharmacy. 2022;45(1):168-169. Chinese.
31. Zhou LJ, Zhang HL, Yang ZZ, et al. Efficacy analysis of drug-loaded microspheres combined with TACE in patients with primary liver cancer. Harbin Medical Journal. 2023;43(5):92-94. Chinese.
32. Li XD. Short-term efficacy of drug-loaded microspheres combined with TACE in patients with primary liver cancer and its effects on serum AFP and CA724 levels. The Journal of Medical Theory and Practice. 2025;38(18):3119-3121. Chinese. doi:10.19381/j.issn.1001-7585.2025.18.016
33. Dai SB, Li HX, Wang XX, et al. Efficacy of drug-loaded microspheres combined with hepatic arterial chemoembolization in patients with primary liver cancer. International Journal of Laboratory Medicine. 2022;43(22):2759-2762. Chinese.
34. Pang HC, Fu J, Zeng LY. Clinical study of drug-loaded microsphere embolization versus conventional chemoembolization in the treatment of unresectable hepatocellular carcinoma. Modern Hospital. 2022;22(12):1966-1968. Chinese.
35. Li HB, Ding J, Li G, et al. Differences in efficacy and adverse reactions between drug-loaded microsphere embolization and conventional treatment for primary liver cancer. Medical Journal of the Chinese People’s Armed Police Forces. 2025;36(7):558-563. Chinese. doi:10.14010/j.cnki.wjyx.2025.07.001
36. Pu CY, Chen Q. Effects of drug-loaded microspheres used during TACE on liver function and immune function in patients with intermediate-advanced liver cancer. Chinese Hepatology. 2020;25(1):42-46. Chinese. doi:10.14000/j.cnki.issn.1008-1704.2020.01.016
37. Jiang FS, Wang M, Zheng W, et al. Comparative study of drug-loaded microspheres and lipiodol arterial chemoembolization in the treatment of massive liver cancer. Jiangxi Medical Journal. 2021;56(7):930-933,964. Chinese.
38. Zhao L, Wang TY, Liu JZ, et al. Value of drug-loaded microspheres in hepatic arterial chemoembolization for primary liver cancer. Journal of Medical Forum. 2020;41(12):133-136,140. Chinese.
39. Zhao W, Dai F, Feng GA. Efficacy of CalliSpheres drug-loaded microsphere transarterial chemoembolization in patients with intermediate-advanced liver cancer. Systems Medicine. 2020;5(24):109-111. Chinese. doi:10.19368/j.cnki.2096-1782.2020.24.109
40. Llovet JM, Real MI, Montaña X, et al. Arterial embolisation or chemoembolisation versus symptomatic treatment in patients with unresectable hepatocellular carcinoma: a randomised controlled trial. Lancet. 2002;359(9319):1734-1739. doi:10.1016/S0140-6736(02)08649-X.
41. Meyer T, Kirkwood A, Roughton M, et al. A randomised phase II/III trial of 3-weekly cisplatin-based sequential transarterial chemoembolisation vs embolisation alone for hepatocellular carcinoma. Br J Cancer. 2013;108(6):1252-1259. doi:10.1038/bjc.2013.85.
42. Salem R, Gordon AC, Mouli S, et al. Y90 Radioembolization Significantly Prolongs Time to Progression Compared With Chemoembolization in Patients With Hepatocellular Carcinoma. Gastroenterology. 2016;151(6):1155-1163.e2. doi:10.1053/j.gastro.2016.08.029.
43. Kolligs FT, Bilbao JI, Jakobs T, et al. Pilot randomized trial of selective internal radiation therapy vs. chemoembolization in unresectable hepatocellular carcinoma. Liver Int. 2015;35(6):1715-1721. doi:10.1111/liv.12750.
44. Kawai S, Okamura J, Ogawa M, et al. Prospective and randomized clinical trial for the treatment of hepatocellular carcinoma--a comparison of lipiodol-transcatheter arterial embolization with and without adriamycin (first cooperative study). The Cooperative Study Group for Liver Cancer Treatment of Japan. Cancer Chemother Pharmacol. 1992;31 Suppl:S1-S6. doi:10.1007/BF00687096.
45. Malagari K, Pomoni M, Kelekis A, et al. Prospective randomized comparison of chemoembolization with doxorubicin-eluting beads and bland embolization with BeadBlock for hepatocellular carcinoma. Cardiovasc Intervent Radiol. 2010;33(3):541-551. doi:10.1007/s00270-009-9750-0.
46. Brown KT, Do RK, Gonen M, et al. Randomized Trial of Hepatic Artery Embolization for Hepatocellular Carcinoma Using Doxorubicin-Eluting Microspheres Compared With Embolization With Microspheres Alone. J Clin Oncol. 2016;34(17):2046-2053. doi:10.1200/JCO.2015.64.0821.
47. Ihira H, Sonomura T, Makitani A, et al. Impact of anticancer drugs on the therapeutic efficacy and side effects of hepatic arterial embolization for hepatocellular carcinoma. JGH Open. 2023;7(12):884-888. Published 2023 Nov 10. doi:10.1002/jgh3.12997.
48. Dhondt E, Lambert B, Hermie L, et al. 90Y Radioembolization versus Drug-eluting Bead Chemoembolization for Unresectable Hepatocellular Carcinoma: Results from the TRACE Phase II Randomized Controlled Trial. Radiology. 2022;303(3):699-710. doi:10.1148/radiol.211806.
49. Raoul JL, Guyader D, Bretagne JF, et al. Prospective randomized trial of chemoembolization versus intra-arterial injection of 131I-labeled-iodized oil in the treatment of hepatocellular carcinoma. Hepatology. 1997;26(5):1156-1161. doi:10.1002/hep.510260511.
50. Yu SC, Hui JW, Hui EP, et al. Unresectable hepatocellular carcinoma: randomized controlled trial of transarterial ethanol ablation versus transcatheter arterial chemoembolization. Radiology. 2014;270(2):607-620. doi:10.1148/radiol.13130498.
51. Sacco R, Bargellini I, Bertini M, et al. Conventional versus doxorubicin-eluting bead transarterial chemoembolization for hepatocellular carcinoma. J Vasc Interv Radiol. 2011;22(11):1545-1552. doi:10.1016/j.jvir.2011.07.002.
52. Pitton MB, Kloeckner R, Ruckes C, et al. Randomized comparison of selective internal radiotherapy (SIRT) versus drug-eluting bead transarterial chemoembolization (DEB-TACE) for the treatment of hepatocellular carcinoma. Cardiovasc Intervent Radiol. 2015;38(2):352-360. doi:10.1007/s00270-014-1012-0.
53. Yang YB, Cheng HY, Zhang L, et al. Interim results of a single-blind randomized controlled trial of pressurized infusion with temporary occlusion of tumor arterial blood flow for hypovascular liver cancer. Chinese Journal of Radiology. 2012;(8):724-729. Chinese.
54. Cheng HY, Chen D, Xu AM. Preliminary study of arterial blood flow occlusion for the treatment of hypovascular malignant hepatic tumors. Chinese Journal of Interventional Imaging and Therapy. 2004;(1):60-64. Chinese. doi:10.13929/j.1672-8475.2004.01.016
55. He SW. Comparative analysis of transcatheter hepatic arterial embolization and hepatic arterial infusion chemoembolization in the treatment of primary liver cancer. Cardiovascular Disease Electronic Journal of Integrated Traditional Chinese and Western Medicine. 2018;6(35):71-72. Chinese. doi:10.16282/j.cnki.cn11-9336/r.2018.35.053
56. Feng T. Effect of hepatic arterial infusion chemoembolization for liver cancer and analysis of postoperative HIF-1α and VEGF indicators. Medical Diet and Health. 2020;18(19):57-58. Chinese.

**Table S3.2: Quantitative assessment of potential effect modifiers by treatment arm**

| **Node** | **Arms / participants** | **BCLC A/B/C** **(reported arms)** | **Child-Pugh A/B** **(reported arms)** | **Tumor-size central value, cm** **(reported arms)** | **Publication years** | **Follow-up, months** |
| --- | --- | --- | --- | --- | --- | --- |
| cTACE | 51 / 2,376 | 172/468/264 (19) | 1,306/647 (42) | 3.85-13.45 (26) | 1992-2026 | 1-51 |
| DEB-TACE | 42 / 1,931 | 157/404/249 (16) | 1,043/528 (34) | 4.10-13.16 (25) | 2009-2025 | 1-43.5 |
| DSM-TACE | 2 / 63 | 11/11/9 (1) | 30/31 (2) | 3.87-6.23 (2) | 2020-2021 | 24 |
| B-TACE | 3 / 104 | 1/7/22 (1) | 65/5 (2) | 8.60-9.10 (2) | 2004-2026 | 6-12 |
| TAE | 9 / 457 | 24/105/44 (4) | 318/116 (9) | 4.70-8.10 (4) | 1992-2023 | 3-51 |
| TARE | 5 / 152 | 23/23/3 (3) | 123/28 (5) | 6.13 (1) | 1997-2022 | 6-48 |

**Appendix 4: List of data extracted from the included randomized clinical trials**

| Data category | List of variables |
| --- | --- |
| Study | First author, year of publication, study type, total number of patients in each group |
| Patients | Sex, age, HBV number, BCLC (A/B/C), Child-Pugh (A/B), ECOG performance status (0/1/2), AFP (ng/ml), maximum tumor diameter (cm), tumor number (single/multiple), follow-up (months) |
| Interventions | intervention measures |
|  |  |
| Efficacy outcome | Objective response rate (ORR), disease control rate (DCR), complete response (CR), partial response (PR), stable disease (SD), progressive disease (PD) |
|  |  |
| Adverse events | Nausea/Vomiting, abdominal pain, fever |

Abbreviations:HBV, Hepatitis B Virus; BCLC, Barcelona Clinic Liver Cancer; Child-Pugh, Child-Pugh classification; ECOG performance status, Eastern Cooperative Oncology Group performance status; AFP, Alpha-fetoprotein.

**Appendix 5: Risk of bias of randomized clinical trials**

**Figure S5.1: Overall risk of bias presented as percentage of each risk of bias item across all included studies. Green = Low risk, Red = High risk, Yellow = Some concerns.**

**Figure S5.2: Study level risk of bias assessment using Cochrane risk of bias tool 2.0 for assessing risk of bias of randomized clinical trials.**

**Appendix 6: Evaluation of inconsistency and heterogeneity**

**Table S6.1: Global consistency and Heterogeneity score**

| **Clinical outcome** | **Chi square** | **P value** | **τ2 network** |
| --- | --- | --- | --- |
| Objective response rate (ORR) | 0.10 | 0.9513 | 0.0153 |
| Disease control rate (DCR) | 0.21 | 0.6459 | 0.0028 |
| Complete response (CR) | 0.01 | 0.9091 | 0.0355 |
| Partial response (PR) | <0.001 | 0.9481 | <0.001 |
| Stable disease (SD) | 2.94 | 0.0843 | 0.017 |
| Progressive disease (PD) | 4.16 | 0.1250 | 0.0429 |
| Nausea, vomiting | <0.001 | 0.9666 | 0.1245 |
| Abdominal pain | 1.96 | 0.1610 | 0.2819 |
| Fever | 0.20 | 0.6538 | 0.0713 |

**Table S6.2: Side-splitting of objective response rate (ORR), n (%). Inconsistency test in transarterial treatment strategies for hepatocellular carcinoma.**

| **Side** | **Direct** | | **Indirect** | | **Difference** | | |
| --- | --- | --- | --- | --- | --- | --- | --- |
|  | **Coef.** | **Std. Err.** | **Coef.** | **Std. Err.** | **Coef.** | **Std. Err.** | **P>z** |
| cTACE vs DEB-TACE | 0.3129628 | 0.0389828 | 0.2708765 | 0.1672661 | 0.0420863 | 0.1713145 | 0.806 |
| cTACE vs TAE | -0.0401108 | 0.1000861 | -0.0329257 | 0.2146635 | -0.0071851 | 0.2369189 | 0.976 |
| cTACE vs TARE | 0.1575208 | 0.1952456 | 0.2333801 | 0.1438098 | -0.0758593 | 0.2420204 | 0.754 |
| DEB-TACE vs TAE | -0.3436472 | 0.2111326 | -0.3508341 | 0.107369 | 0.0071869 | 0.2369189 | 0.976 |
| DEB-TACE vs TARE | -0.0796402 | 0.1385585 | -0.1555003 | 0.1984325 | 0.0758601 | 0.2420205 | 0.754 |

Abbreviations: Conventional Transarterial Chemoembolization (c-TACE); Drug-eluting bead Transarterial Chemoembolization (DEB-TACE); Transarterial Embolization (TAE); Transarterial Radioembolization (TARE)

**Table S6.3: Side-splitting of disease control rate (DCR), n (%). Inconsistency test in transarterial treatment strategies for hepatocellular carcinoma.**

| **Side** | **Direct** | | **Indirect** | | **Difference** | | |
| --- | --- | --- | --- | --- | --- | --- | --- |
|  | **Coef.** | **Std. Err.** | **Coef.** | **Std. Err.** | **Coef.** | **Std. Err.** | **P>z** |
| cTACE vs DEB-TACE | 0.1492922 | 0.0202696 | 0.097938 | 0.1098505 | 0.0513541 | 0.1117581 | 0.646 |
| cTACE vs TAE | -0.0756516 | 0.060467 | -0.0242978 | 0.0940438 | -0.0513538 | 0.1117583 | 0.646 |
| DEB-TACE vs TAE | -0.1735898 | 0.0927573 | -0.2249437 | 0.0651908 | 0.0513539 | 0.1117586 | 0.646 |

Abbreviations: Conventional Transarterial Chemoembolization (c-TACE); Drug-eluting bead Transarterial Chemoembolization (DEB-TACE); Transarterial Embolization (TAE)

**Table S6.4: Side-splitting of complete response (CR), n (%). Inconsistency test in transarterial treatment strategies for hepatocellular carcinoma.**

| Side | Direct | | Indirect | | Difference | | |
| --- | --- | --- | --- | --- | --- | --- | --- |
|  | Coef. | Std. Err. | Coef. | Std. Err. | Coef. | Std. Err. | P>z |
| cTACE vs DEB-TACE | 0.3847238 | 0.075492 | 0.434768 | 0.4285814 | -0.0500442 | 0.4382389 | 0.909 |
| cTACE vs TAE | 0.0582621 | 0.2490491 | 0.0082019 | 0.3566263 | 0.0500603 | 0.4382451 | 0.909 |
| DEB-TACE vs TAE | -0.3765164 | 0.3483469 | -0.3264639 | 0.2650653 | -0.0500525 | 0.4382442 | 0.909 |

Abbreviations: Conventional Transarterial Chemoembolization (c-TACE); Drug-eluting bead Transarterial Chemoembolization (DEB-TACE); Transarterial Embolization (TAE)

**Table S6.5: Side-splitting of partial response (PR), n (%). Inconsistency test in transarterial treatment strategies for hepatocellular carcinoma.**

| Side | Direct | | Indirect | | Difference | | |
| --- | --- | --- | --- | --- | --- | --- | --- |
|  | Coef. | Std. Err. | Coef. | Std. Err. | Coef. | Std. Err. | P>z |
| cTACE vs DEB-TACE | 0.2572706 | 0.0444622 | 0.2773751 | 0.3056089 | -0.0201045 | 0.3088263 | 0.948 |
| cTACE vs TAE | 0.0283424 | 0.0974132 | 0.0082299 | 0.2930649 | 0.0201124 | 0.3088306 | 0.948 |
| DEB-TACE vs TAE | -0.2490372 | 0.2896716 | -0.2289285 | 0.1070804 | -0.0201086 | 0.3088296 | 0.948 |

Abbreviations: Conventional Transarterial Chemoembolization (c-TACE); Drug-eluting bead Transarterial Chemoembolization (DEB-TACE); Transarterial Embolization (TAE)

**Table S6.6: Side-splitting of stable disease (SD), n (%). Inconsistency test in transarterial treatment strategies for hepatocellular carcinoma.**

| Side | Direct | | Indirect | | Difference | | |
| --- | --- | --- | --- | --- | --- | --- | --- |
|  | Coef. | Std. Err. | Coef. | Std. Err. | Coef. | Std. Err. | P>z |
| cTACE vs DEB-TACE | -0.3068565 | 0.0734881 | 0.6782863 | 0.5648566 | -0.9851429 | 0.5707046 | 0.841 |
| cTACE vs TAE | -0.1233656 | 0.1888148 | -1.108551 | 0.5388269 | 0.985185 | 0.5707224 | 0.841 |
| DEB-TACE vs TAE | -0.8016895 | 0.5324968 | 0.1834905 | 0.2022737 | -0.9851801 | 0.5707184 | 0.841 |

Abbreviations: Conventional Transarterial Chemoembolization (c-TACE); Drug-eluting bead Transarterial Chemoembolization (DEB-TACE); Transarterial Embolization (TAE)

**Table S6.7: Side-splitting of progressive disease (PD), n (%). Inconsistency test in transarterial treatment strategies for hepatocellular carcinoma.**

| Side | Direct | | Indirect | | Difference | | |
| --- | --- | --- | --- | --- | --- | --- | --- |
|  | Coef. | Std. Err. | Coef. | Std. Err. | Coef. | Std. Err. | P>z |
| cTACE vs DEB-TACE | -1.009103 | 0.1223374 | -0.191284 | 0.5859922 | -0.8178191 | 0.6022827 | 0.175 |
| cTACE vs TAE | 0.7713946 | 0.4174541 | 0.3553008 | 0.4921563 | 0.4160939 | 0.6457968 | 0.519 |
| cTACE vs TARE | 0.1420172 | 0.7988281 | -2.694025 | 1.234316 | 2.836042 | 1.470434 | 0.544 |
| DEB-TACE vs TAE | 1.345421 | 0.4771215 | 1.761548 | 0.4352125 | -0.4161276 | 0.6457926 | 0.519 |
| DEB-TACE vs TARE | -1.704717 | 1.228559 | 1.131284 | 0.8079345 | -2.836001 | 1.470398 | 0.544 |

Abbreviations: Conventional Transarterial Chemoembolization (c-TACE); Drug-eluting bead Transarterial Chemoembolization (DEB-TACE); Transarterial Embolization (TAE); Transarterial Radioembolization (TARE)

**Table S6.8: Side-splitting of nausea, vomiting, n (%). Inconsistency test in transarterial treatment strategies for hepatocellular carcinoma.**

| Side | Direct | | Indirect | | Difference | | |
| --- | --- | --- | --- | --- | --- | --- | --- |
|  | Coef. | Std. Err. | Coef. | Std. Err. | Coef. | Std. Err. | P>z |
| cTACE vs DEB-TACE | -0.4618999 | 0.1475349 | -0.4045481 | 1.358513 | -0.0573518 | 1.366501 | 0.967 |
| cTACE vs B-TACE | -1.738271 | 0.6421888 | -1.795546 | 1.206336 | 0.0572757 | 1.366621 | 0.967 |
| DEB-TACE vs B-TACE | -1.333679 | 1.197263 | -1.276362 | 0.6589154 | -0.0573164 | 1.366595 | 0.967 |

Abbreviations: Conventional Transarterial Chemoembolization (c-TACE); Drug-eluting bead Transarterial Chemoembolization (DEB-TACE); Balloon occluded TACE (B-TACE)

**Table S6.9: Side-splitting of abdominal pain, n (%). Inconsistency test in transarterial treatment strategies for hepatocellular carcinoma.**

| **Side** | **Direct** | | **Indirect** | | **Difference** | | |
| --- | --- | --- | --- | --- | --- | --- | --- |
|  | **Coef.** | **Std. Err.** | **Coef.** | **Std. Err.** | **Coef.** | **Std. Err.** | **P>z** |
| cTACE vs DEB-TACE | -0.4922908 | 0.1862533 | 0.7274764 | 0.8499352 | -1.219767 | 0.870243 | 0.161 |
| cTACE vs TAE | 0.3548439 | 0.4236851 | -0.8649578 | 0.7600032 | 1.219802 | 0.8702623 | 0.161 |
| DEB-TACE vs TAE | -0.3726737 | 0.7368281 | 0.847134 | 0.4630786 | -1.219808 | 0.8702618 | 0.161 |

Abbreviations: Conventional Transarterial Chemoembolization (c-TACE); Drug-eluting bead Transarterial Chemoembolization (DEB-TACE); Transarterial Embolization (TAE)

**Table S6.10: Side-splitting of fever, n (%). Inconsistency test in transarterial treatment strategies for hepatocellular carcinoma.**

| Side | Direct | | Indirect | | Difference | | |
| --- | --- | --- | --- | --- | --- | --- | --- |
|  | Coef. | Std. Err. | Coef. | Std. Err. | Coef. | Std. Err. | P>z |
| cTACE vs DEB-TACE | -0.3903889 | 0.1414084 | 0.2831961 | 1.494173 | -0.673585 | 1.501952 | 0.654 |
| cTACE vs TAE | 0.41001 | 0.3248932 | -0.263635 | 1.465427 | 0.673645 | 1.502113 | 0.654 |
| DEB-TACE vs TAE | 0.1267462 | 1.458553 | 0.8003994 | 0.3589753 | -0.6736532 | 1.502076 | 0.654 |

Abbreviations: Conventional Transarterial Chemoembolization (c-TACE); Drug-eluting bead Transarterial Chemoembolization (DEB-TACE); Transarterial Embolization (TAE)

**Appendix 7: Network maps and forest plots of secondary outcomes**

**Figure S7.1: Network map of the effect on disease control rate (DCR), and forest plot of network effect sizes for compared with cTACE. The size of the nodes was proportional to the number of participants included in the trial, and the thickness of lines between the interventions relates to the number of studies for that comparison.**

**Figure S7.2: Network map of the effect on complete response (CR), and forest plot of network effect sizes for compared with cTACE. The size of the nodes was proportional to the number of participants included in the trial, and the thickness of lines between the interventions relates to the number of studies for that comparison.**

**Figure S7.3: Network map of the effect on partial response (PR), and forest plot of network effect sizes for compared with cTACE. The size of the nodes was proportional to the number of participants included in the trial, and the thickness of lines between the interventions relates to the number of studies for that comparison.**

**Figure S7.4: Network map of the effect on stable disease (SD), and forest plot of network effect sizes for compared with cTACE. The size of the nodes was proportional to the number of participants included in the trial, and the thickness of lines between the interventions relates to the number of studies for that comparison.**

**Figure S7.5: Network map of the effect on progressive disease (PD), and forest plot of network effect sizes for compared with cTACE. The size of the nodes was proportional to the number of participants included in the trial, and the thickness of lines between the interventions relates to the number of studies for that comparison.**

**Figure S7.6: Network map of the effect on nausea, vomiting, and forest plot of network effect sizes for compared with cTACE. The size of the nodes was proportional to the number of participants included in the trial, and the thickness of lines between the interventions relates to the number of studies for that comparison.**

**Figure S7.7: Network map of the effect on abdominal pain, and forest plot of network effect sizes for compared with cTACE. The size of the nodes was proportional to the number of participants included in the trial, and the thickness of lines between the interventions relates to the number of studies for that comparison.**

**Figure S7.8: Network map of the effect on fever, and forest plot of network effect sizes for compared with cTACE. The size of the nodes was proportional to the number of participants included in the trial, and the thickness of lines between the interventions relates to the number of studies for that comparison.**

**Appendix 8: SUCRA and cumulative probability plots**

**Figure S8.1: Cumulative ranking curve plots of transarterial treatment strategies in the network meta-analysis of hepatocellular carcinoma. Higher surface under the curve reflects higher probability of association with successful objective response rate (ORR).**

**Table S8.1: SUCRA rankings of objective response rate (ORR) achievement for transarterial treatment strategies for hepatocellular carcinoma.**

| Treatm~t | SUCRA | PrBest | MeanRank |
| --- | --- | --- | --- |
| cTACE | 33.9 | 0 | 4.3 |
| DEB-TACE | 76.5 | 0.2 | 2.2 |
| DSM-TACE | 0 | 0 | 6 |
| B-TACE | 99.9 | 99.8 | 1 |
| TAE | 27.8 | 0 | 4.6 |
| TARE | 61.9 | 0.1 | 2.9 |

Abbreviations: c-TACE, conventional transarterial chemoembolization; DEB-TACE, drug-eluting bead transarterial chemoembolization; DSM-TACE, degradable starch microspheres transarterial chemoembolization; B-TACE, balloon occluded transarterial chemoembolization; TAE, transarterial embolization; TARE, transarterial radioembolization

**Figure S8.2: Cumulative ranking curve plots of transarterial treatment strategies in the network meta-analysis of hepatocellular carcinoma. Higher surface under the curve reflects higher probability of association with successful disease control rate (DCR).**

**Table S8.2: SUCRA rankings of disease control rate (DCR) achievement for transarterial treatment strategies for hepatocellular carcinoma.**

| Treatm~t | SUCRA | PrBest | MeanRank |
| --- | --- | --- | --- |
| cTACE | 34.6 | 0 | 4.3 |
| DEB-TACE | 74.9 | 1.8 | 2.3 |
| DSM-TACE | 57.3 | 2.3 | 3.1 |
| B-TACE | 98.9 | 95.8 | 1.1 |
| TAE | 11.8 | 0 | 5.4 |
| TARE | 22.5 | 0.1 | 4.9 |

Abbreviations: c-TACE, conventional transarterial chemoembolization; DEB-TACE, drug-eluting bead transarterial chemoembolization; DSM-TACE, degradable starch microspheres transarterial chemoembolization; B-TACE, balloon occluded transarterial chemoembolization; TAE, transarterial embolization; TARE, transarterial radioembolization

**Figure S8.3: Cumulative ranking curve plots of transarterial treatment strategies in the network meta-analysis of hepatocellular carcinoma. Higher surface under the curve reflects higher probability of association with successful complete response (CR).**

**Table S8.3: SUCRA rankings of complete response (CR) achievement for transarterial treatment strategies for hepatocellular carcinoma.**

| Treatm~t | SUCRA | PrBest | MeanRank |
| --- | --- | --- | --- |
| cTACE | 16.5 | 0.0 | 5.2 |
| DEB-TACE | 51.8 | 0.5 | 3.4 |
| DSM-TACE | 78.9 | 52.2 | 2.1 |
| B-TACE | 77.8 | 31.0 | 2.1 |
| TAE | 21.7 | 0.0 | 4.9 |
| TARE | 53.3 | 16.3 | 3.3 |

Abbreviations: c-TACE, conventional transarterial chemoembolization; DEB-TACE, drug-eluting bead transarterial chemoembolization; DSM-TACE, degradable starch microspheres transarterial chemoembolization; B-TACE, balloon occluded transarterial chemoembolization; TAE, transarterial embolization; TARE, transarterial radioembolization

**Figure S8.4: Cumulative ranking curve plots of transarterial treatment strategies in the network meta-analysis of hepatocellular carcinoma. Higher surface under the curve reflects higher probability of association with successful partial response (PR).**

**Table S8.4: SUCRA rankings of partial response (PR) achievement for transarterial treatment strategies for hepatocellular carcinoma.**

| Treatm~t | SUCRA | PrBest | MeanRank |
| --- | --- | --- | --- |
| cTACE | 11.3 | 0.0 | 5.4 |
| DEB-TACE | 58.9 | 0.0 | 3.1 |
| DSM-TACE | 84.7 | 53.5 | 1.8 |
| B-TACE | 89.2 | 46.5 | 1.5 |
| TAE | 18.5 | 0.0 | 5.1 |
| TARE | 37.3 | 0.0 | 4.1 |

Abbreviations: c-TACE, conventional transarterial chemoembolization; DEB-TACE, drug-eluting bead transarterial chemoembolization; DSM-TACE, degradable starch microspheres transarterial chemoembolization; B-TACE, balloon occluded transarterial chemoembolization; TAE, transarterial embolization; TARE, transarterial radioembolization

**Figure S8.5: Cumulative ranking curve plots of transarterial treatment strategies in the network meta-analysis of hepatocellular carcinoma. Higher surface under the curve reflects higher probability of association with successful stable disease (SD).**

**Table S8.5: SUCRA rankings of stable disease (SD) achievement for transarterial treatment strategies for hepatocellular carcinoma.**

| Treatm~t | SUCRA | PrBest | MeanRank |
| --- | --- | --- | --- |
| cTACE | 91.6 | 64.5 | 1.4 |
| DEB-TACE | 41.2 | 0.0 | 3.9 |
| DSM-TACE | 44.7 | 8.3 | 3.8 |
| B-TACE | 35.5 | 6.8 | 4.2 |
| TAE | 52.6 | 9.1 | 3.4 |
| TARE | 34.3 | 11.3 | 4.3 |

Abbreviations: c-TACE, conventional transarterial chemoembolization; DEB-TACE, drug-eluting bead transarterial chemoembolization; DSM-TACE, degradable starch microspheres transarterial chemoembolization; B-TACE, balloon occluded transarterial chemoembolization; TAE, transarterial embolization; TARE, transarterial radioembolization

**Figure S8.6: Cumulative ranking curve plots of transarterial treatment strategies in the network meta-analysis of hepatocellular carcinoma. Higher surface under the curve reflects higher probability of association with successful progressive disease (PD).**

**Table S8.6: SUCRA rankings of progressive disease (PD) achievement for transarterial treatment strategies for hepatocellular carcinoma.**

| Treatm~t | SUCRA | PrBest | MeanRank |
| --- | --- | --- | --- |
| cTACE | 25.4 | 0.0 | 4.7 |
| DEB-TACE | 63.2 | 2.9 | 2.8 |
| DSM-TACE | 69.0 | 39.5 | 2.6 |
| B-TACE | 86.7 | 48.9 | 1.7 |
| TAE | 2.8 | 0.0 | 5.9 |
| TARE | 52.9 | 8.6 | 3.4 |

Abbreviations: c-TACE, conventional transarterial chemoembolization; DEB-TACE, drug-eluting bead transarterial chemoembolization; DSM-TACE, degradable starch microspheres transarterial chemoembolization; B-TACE, balloon occluded transarterial chemoembolization; TAE, transarterial embolization; TARE, transarterial radioembolization

**Figure S8.7: Cumulative ranking curve plots of transarterial treatment strategies in the network meta-analysis of hepatocellular carcinoma. Higher surface under the curve reflects higher occurrence of association with nausea, vomiting.**

**Table S8.7: SUCRA rankings for occurrence of nausea, vomiting achievement for transarterial treatment strategies for hepatocellular carcinoma.**

| Treatm~t | SUCRA | PrBest | MeanRank |
| --- | --- | --- | --- |
| cTACE | 12.0 | 0.0 | 3.6 |
| DEB-TACE | 54.7 | 0.9 | 2.4 |
| DSM-TACE | 35.0 | 3.5 | 3.0 |
| TAE | 98.3 | 95.6 | 1.1 |

Abbreviations: c-TACE, conventional transarterial chemoembolization; DEB-TACE, drug-eluting bead transarterial chemoembolization; DSM-TACE, degradable starch microspheres transarterial chemoembolization; TAE, transarterial embolization

**Figure S8.8: Cumulative ranking curve plots of transarterial treatment strategies in the network meta-analysis of hepatocellular carcinoma. Higher surface under the curve reflects higher occurrence of association with abdominal pain.**

**Table S8.8: SUCRA rankings for occurrence of abdominal pain achievement for transarterial treatment strategies for hepatocellular carcinoma.**

| Treatm~t | SUCRA | PrBest | MeanRank |
| --- | --- | --- | --- |
| cTACE | 16.3 | 0.0 | 5.2 |
| DEB-TACE | 48.9 | 0.4 | 3.6 |
| DSM-TACE | 57.5 | 6.6 | 3.1 |
| B-TACE | 69.5 | 12.9 | 2.5 |
| TAE | 16.0 | 0.1 | 5.2 |
| TARE | 91.9 | 80.0 | 1.4 |

Abbreviations: c-TACE, conventional transarterial chemoembolization; DEB-TACE, drug-eluting bead transarterial chemoembolization; DSM-TACE, degradable starch microspheres transarterial chemoembolization; B-TACE, balloon occluded transarterial chemoembolization; TAE, transarterial embolization; TARE, transarterial radioembolization

**Figure S8.9: Cumulative ranking curve plots of transarterial treatment strategies in the network meta-analysis of hepatocellular carcinoma. Higher surface under the curve reflects higher occurrence of association with fever.**

**Table S8.9: SUCRA rankings for occurrence of fever achievement for transarterial treatment strategies for hepatocellular carcinoma.**

| Treatm~t | SUCRA | PrBest | MeanRank |
| --- | --- | --- | --- |
| cTACE | 29.4 | 0.0 | 4.5 |
| DEB-TACE | 55.5 | 0.1 | 3.2 |
| DSM-TACE | 93.4 | 71.8 | 1.3 |
| B-TACE | 77.1 | 22.5 | 2.1 |
| TAE | 10.9 | 0.0 | 5.5 |
| TARE | 33.7 | 5.6 | 4.3 |

Abbreviations: c-TACE, conventional transarterial chemoembolization; DEB-TACE, drug-eluting bead transarterial chemoembolization; DSM-TACE, degradable starch microspheres transarterial chemoembolization; B-TACE, balloon occluded transarterial chemoembolization; TAE, transarterial embolization; TARE, transarterial radioembolization

**Appendix 9: League tables of summary estimates for transarterial treatment strategies in the treatment of hepatocellular carcinoma derived from network meta-analysis of 56 included studies**

**Table S9.1: Objective response rate (ORR)**

This league table presents network estimates for objective response rate (ORR). Each cell reports the risk ratio (RR) and 95% confidence interval (CI) for the treatment in the row compared with the treatment in the column. For this beneficial outcome, an RR < 1 favours the column treatment and an RR > 1 favours the row treatment. For example, the RR for ORR with cTACE versus DEB-TACE is 0.73 (95% CI 0.68-0.79).

| cTACE |  |  |  |  |  |
| --- | --- | --- | --- | --- | --- |
| 0.73 (0.68,0.79) | DEB-TACE |  |  |  |  |
| 2.71 (1.57,4.67) | 3.69 (2.14,6.39) | DSM-TACE |  |  |  |
| 0.29 (0.16,0.52) | 0.39 (0.21,0.71) | 0.11 (0.05,0.24) | B-TACE |  |  |
| 1.04 (0.87,1.24) | 1.42 (1.18,1.71) | 0.38 (0.22,0.68) | 3.64 (1.95,6.78) | TAE |  |
| 0.81 (0.65,1.02) | 1.11 (0.89,1.38) | 0.30 (0.17,0.54) | 2.85 (1.51,5.39) | 0.78 (0.59,1.04) | TARE |

Abbreviations: c-TACE, conventional transarterial chemoembolization; DEB-TACE, drug-eluting bead transarterial chemoembolization; DSM-TACE, degradable starch microspheres transarterial chemoembolization; B-TACE, balloon occluded transarterial chemoembolization; TAE, transarterial embolization; TARE, transarterial radioembolization

**Table S9.2: Disease control rate (DCR)**

This league table presents network estimates for disease control rate (DCR). Each cell reports the risk ratio (RR) and 95% confidence interval (CI) for the treatment in the row compared with the treatment in the column. For this beneficial outcome, an RR < 1 favours the column treatment and an RR > 1 favours the row treatment. For example, the RR for DCR with cTACE versus DEB-TACE is 0.86 (95% CI 0.83-0.90).

| cTACE |  |  |  |  |  |
| --- | --- | --- | --- | --- | --- |
| 0.86 (0.83,0.90) | DEB-TACE |  |  |  |  |
| 0.92 (0.76,1.11) | 1.06 (0.88,1.29) | DSM-TACE |  |  |  |
| 0.68 (0.54,0.86) | 0.79 (0.63,1.00) | 0.74 (0.55,1.00) | B-TACE |  |  |
| 1.06 (0.96,1.17) | 1.23 (1.11,1.36) | 1.16 (0.94,1.43) | 1.56 (1.21,2.00) | TAE |  |
| 1.04 (0.88,1.21) | 1.20 (1.02,1.41) | 1.13 (0.88,1.44) | 1.52 (1.15,2.01) | 0.97 (0.81,1.17) | TARE |

Abbreviations: c-TACE, conventional transarterial chemoembolization; DEB-TACE, drug-eluting bead transarterial chemoembolization; DSM-TACE, degradable starch microspheres transarterial chemoembolization; B-TACE, balloon occluded transarterial chemoembolization; TAE, transarterial embolization; TARE, transarterial radioembolization

**Table S9.3: Complete response (CR)**

This league table presents network estimates for complete response (CR). Each cell reports the risk ratio (RR) and 95% confidence interval (CI) for the treatment in the row compared with the treatment in the column. For this beneficial outcome, an RR < 1 favours the column treatment and an RR > 1 favours the row treatment. For example, the RR for CR with cTACE versus DEB-TACE is 0.68 (95% CI 0.59-0.79).

| cTACE |  |  |  |  |  |
| --- | --- | --- | --- | --- | --- |
| 0.68 (0.59,0.79) | DEB-TACE |  |  |  |  |
| 0.15 (0.01,2.90) | 0.23 (0.01,4.28) | DSM-TACE |  |  |  |
| 0.23 (0.04,1.17) | 0.34 (0.07,1.74) | 1.49 (0.05,43.11) | B-TACE |  |  |
| 0.96 (0.65,1.42) | 1.41 (0.93,2.12) | 6.23 (0.32,121.02) | 4.18 (0.78,22.48) | TAE |  |
| 0.46 (0.04,5.37) | 0.68 (0.06,7.92) | 3.00 (0.07,138.39) | 2.01 (0.11,38.51) | 0.48 (0.04,5.79) | TARE |

Abbreviations: c-TACE, conventional transarterial chemoembolization; DEB-TACE, drug-eluting bead transarterial chemoembolization; DSM-TACE, degradable starch microspheres transarterial chemoembolization; B-TACE, balloon occluded transarterial chemoembolization; TAE, transarterial embolization; TARE, transarterial radioembolization

**Table S9.4: Partial response (PR)**

This league table presents network estimates for partial response (PR). Each cell reports the risk ratio (RR) and 95% confidence interval (CI) for the treatment in the row compared with the treatment in the column. For this beneficial outcome, an RR < 1 favours the column treatment and an RR > 1 favours the row treatment. For example, the RR for PR with cTACE versus DEB-TACE is 0.77 (95% CI 0.71-0.84).

| cTACE |  |  |  |  |  |
| --- | --- | --- | --- | --- | --- |
| 0.77 (0.71,0.84) | DEB-TACE |  |  |  |  |
| 0.31 (0.07,1.35) | 0.40 (0.09,1.75) | DSM-TACE |  |  |  |
| 0.33 (0.18,0.60) | 0.42 (0.23,0.78) | 1.06 (0.21,5.25) | B-TACE |  |  |
| 0.97 (0.81,1.17) | 1.26 (1.04,1.53) | 3.17 (0.71,14.03) | 2.98 (1.58,5.61) | TAE |  |
| 0.88 (0.70,1.10) | 1.14 (0.90,1.44) | 2.85 (0.64,12.71) | 2.69 (1.41,5.12) | 0.90 (0.68,1.20) | TARE |

Abbreviations: c-TACE, conventional transarterial chemoembolization; DEB-TACE, drug-eluting bead transarterial chemoembolization; DSM-TACE, degradable starch microspheres transarterial chemoembolization; B-TACE, balloon occluded transarterial chemoembolization; TAE, transarterial embolization; TARE, transarterial radioembolization

**Table S9.5: Stable disease (SD)**

This league table presents network estimates for stable disease (SD). Each cell reports the risk ratio (RR) and 95% confidence interval (CI) for the treatment in the row compared with the treatment in the column. An RR < 1 indicates a lower probability of stable disease with the row treatment, whereas an RR > 1 indicates a higher probability. For example, the RR for SD with cTACE versus DEB-TACE is 1.34 (95% CI 1.16-1.54).

| cTACE |  |  |  |  |  |
| --- | --- | --- | --- | --- | --- |
| 1.34 (1.16,1.54) | DEB-TACE |  |  |  |  |
| 1.33 (0.86,2.06) | 0.99 (0.63,1.57) | DSM-TACE |  |  |  |
| 1.42 (0.86,2.35) | 1.06 (0.63,1.78) | 1.07 (0.55,2.08) | B-TACE |  |  |
| 1.26 (0.87,1.82) | 0.94 (0.65,1.37) | 0.95 (0.54,1.68) | 0.89 (0.48,1.64) | TAE |  |
| 1.47 (0.77,2.83) | 1.10 (0.56,2.14) | 1.11 (0.51,2.43) | 1.04 (0.45,2.36) | 1.17 (0.55,2.46) | TARE |

Abbreviations: c-TACE, conventional transarterial chemoembolization; DEB-TACE, drug-eluting bead transarterial chemoembolization; DSM-TACE, degradable starch microspheres transarterial chemoembolization; B-TACE, balloon occluded transarterial chemoembolization; TAE, transarterial embolization; TARE, transarterial radioembolization

**Table S9.6: Progressive disease (PD)**

This league table presents network estimates for progressive disease (PD). Each cell reports the odds ratio (OR) and 95% confidence interval (CI) for the treatment in the row compared with the treatment in the column. Because PD is undesirable, an OR < 1 indicates lower odds with the row treatment and therefore favours the row treatment, whereas an OR > 1 indicates higher odds. For example, the OR for PD with cTACE versus DEB-TACE is 2.65 (95% CI 2.10-3.33).

| cTACE |  |  |  |  |  |
| --- | --- | --- | --- | --- | --- |
| 2.65 (2.10,3.33) | DEB-TACE |  |  |  |  |
| 3.52 (0.33,37.51) | 1.33 (0.12,14.33) | DSM-TACE |  |  |  |
| 4.52 (2.03,10.09) | 1.71 (0.74,3.93) | 1.28 (0.11,15.62) | B-TACE |  |  |
| 0.55 (0.30,1.02) | 0.21 (0.11,0.39) | 0.16 (0.01,1.80) | 0.12 (0.04,0.33) | TAE |  |
| 2.01 (0.54,7.46) | 0.76 (0.20,2.84) | 0.57 (0.04,8.52) | 0.44 (0.10,2.07) | 3.65 (0.86,15.51) | TARE |

Abbreviations: c-TACE, conventional transarterial chemoembolization; DEB-TACE, drug-eluting bead transarterial chemoembolization; DSM-TACE, degradable starch microspheres transarterial chemoembolization; B-TACE, balloon occluded transarterial chemoembolization; TAE, transarterial embolization; TARE, transarterial radioembolization

**Table S9.7: Nausea, vomiting**

This league table presents network estimates for nausea or vomiting events. Each cell reports the odds ratio (OR) and 95% confidence interval (CI) for the treatment in the row compared with the treatment in the column. Because nausea or vomiting is undesirable, an OR < 1 indicates lower odds with the row treatment and therefore favours the row treatment, whereas an OR > 1 indicates higher odds. For example, the OR for nausea or vomiting with cTACE versus DEB-TACE is 1.58 (95% CI 1.19-2.11).

| cTACE |  |  |  |
| --- | --- | --- | --- |
| 1.58 (1.19,2.11) | DEB-TACE |  |  |
| 1.27 (0.35,4.52) | 0.80 (0.22,2.95) | DSM-TACE |  |
| 5.76 (1.91,17.36) | 3.63 (1.18,11.19) | 4.55 (0.84,24.50) | TAE |

Abbreviations: c-TACE, conventional transarterial chemoembolization; DEB-TACE, drug-eluting bead transarterial chemoembolization; DSM-TACE, degradable starch microspheres transarterial chemoembolization; TAE, transarterial embolization

**Table S9.8: Abdominal pain**

This league table presents network estimates for abdominal pain events. Each cell reports the odds ratio (OR) and 95% confidence interval (CI) for the treatment in the row compared with the treatment in the column. Because abdominal pain is undesirable, an OR < 1 indicates lower odds with the row treatment and therefore favours the row treatment, whereas an OR > 1 indicates higher odds. For example, the OR for abdominal pain with cTACE versus DEB-TACE is 1.55 (95% CI 1.08-2.22).

| cTACE |  |  |  |  |  |
| --- | --- | --- | --- | --- | --- |
| 1.55 (1.08,2.22) | DEB-TACE |  |  |  |  |
| 2.22 (0.51,9.65) | 1.44 (0.32,6.51) | DSM-TACE |  |  |  |
| 3.15 (0.70,14.10) | 2.03 (0.44,9.51) | 1.42 (0.17,11.55) | B-TACE |  |  |
| 0.93 (0.45,1.95) | 0.60 (0.28,1.31) | 0.42 (0.08,2.17) | 0.30 (0.06,1.58) | TAE |  |
| 17.09 (0.80,366.80) | 11.04 (0.50,242.13) | 7.69 (0.26,230.35) | 5.43 (0.18,164.82) | 18.28 (0.78,427.96) | TARE |

Abbreviations: c-TACE, conventional transarterial chemoembolization; DEB-TACE, drug-eluting bead transarterial chemoembolization; DSM-TACE, degradable starch microspheres transarterial chemoembolization; B-TACE, balloon occluded transarterial chemoembolization; TAE, transarterial embolization; TARE, transarterial radioembolization

**Table S9.9: Fever**

This league table presents network estimates for fever events. Each cell reports the odds ratio (OR) and 95% confidence interval (CI) for the treatment in the row compared with the treatment in the column. Because fever is undesirable, an OR < 1 indicates lower odds with the row treatment and therefore favours the row treatment, whereas an OR > 1 indicates higher odds. For example, the OR for fever with cTACE versus DEB-TACE is 1.47 (95% CI 1.12-1.93).

| cTACE |  |  |  |  |  |
| --- | --- | --- | --- | --- | --- |
| 1.47 (1.12,1.93) | DEB-TACE |  |  |  |  |
| 6.72 (1.82,24.83) | 4.58 (1.21,17.41) | DSM-TACE |  |  |  |
| 3.35 (0.81,13.89) | 2.28 (0.53,9.73) | 0.50 (0.07,3.44) | B-TACE |  |  |
| 0.69 (0.37,1.26) | 0.47 (0.24,0.91) | 0.10 (0.02,0.43) | 0.21 (0.04,0.96) | TAE |  |
| 0.94 (0.09,9.70) | 0.64 (0.06,6.72) | 0.14 (0.01,2.03) | 0.28 (0.02,4.33) | 1.38 (0.12,15.30) | TARE |

Abbreviations: c-TACE, conventional transarterial chemoembolization; DEB-TACE, drug-eluting bead transarterial chemoembolization; DSM-TACE, degradable starch microspheres transarterial chemoembolization; B-TACE, balloon occluded transarterial chemoembolization; TAE, transarterial embolization; TARE, transarterial radioembolization

**Appendix 10: CINeMA Assessment**

We use the CINeMA framework to evidence certainty, assessing it for each network estimate based on the following criteria:

**A: Within study bias:** We classified the overall risk of bias for each study as low risk of bias, the risk of bias as moderate when none of the four assessed risk of bias items were rated as high risk, and the risk of bias as high when one or both items were rated as high risk. See Appendix 4 for the bias assessment. The risk of bias for a pairwise comparison of each transarterial treatment strategies is shown in figure S10.1-10.20.

**Figure S10.1: Risk of bias contribution by intervention group in objective response rate (ORR)**

**Figure S10.2: Overall risk of bias by treatment comparison in objective response rate (ORR)**

Abbreviations: c-TACE, conventional transarterial chemoembolization; DEB-TACE, drug-eluting bead transarterial chemoembolization; DSM-TACE, degradable starch microspheres transarterial chemoembolization; B-TACE, balloon occluded transarterial chemoembolization; TAE, transarterial embolization; TARE, transarterial radioembolization

**Figure S10.3: Risk of bias contribution by intervention group in disease control rate (DCR)**

**Figure S10.4: Overall risk of bias by treatment comparison in disease control rate (DCR)**

Abbreviations: c-TACE, conventional transarterial chemoembolization; DEB-TACE, drug-eluting bead transarterial chemoembolization; DSM-TACE, degradable starch microspheres transarterial chemoembolization; B-TACE, balloon occluded transarterial chemoembolization; TAE, transarterial embolization; TARE, transarterial radioembolization

**Figure S10.5: Risk of bias contribution by intervention group in complete response (CR)**

**Figure S10.6: Overall risk of bias by treatment comparison in complete response (CR)**

Abbreviations: c-TACE, conventional transarterial chemoembolization; DEB-TACE, drug-eluting bead transarterial chemoembolization; DSM-TACE, degradable starch microspheres transarterial chemoembolization; B-TACE, balloon occluded transarterial chemoembolization; TAE, transarterial embolization; TARE, transarterial radioembolization

**Figure S10.7: Risk of bias contribution by intervention group in partial response (PR)**

**Figure S10.8: Overall risk of bias by treatment comparison in partial response (PR)**

Abbreviations: c-TACE, conventional transarterial chemoembolization; DEB-TACE, drug-eluting bead transarterial chemoembolization; DSM-TACE, degradable starch microspheres transarterial chemoembolization; B-TACE, balloon occluded transarterial chemoembolization; TAE, transarterial embolization; TARE, transarterial radioembolization

**Figure S10.9: Risk of bias contribution by intervention group in stable disease (SD)**

**Figure S10.10: Overall risk of bias by treatment comparison in stable disease (SD)**

Abbreviations: c-TACE, conventional transarterial chemoembolization; DEB-TACE, drug-eluting bead transarterial chemoembolization; DSM-TACE, degradable starch microspheres transarterial chemoembolization; B-TACE, balloon occluded transarterial chemoembolization; TAE, transarterial embolization; TARE, transarterial radioembolization

**Figure S10.11: Risk of bias contribution by intervention group in progressive disease (PD)**

**Figure S10.12: Overall risk of bias by treatment comparison in progressive disease (PD)**

Abbreviations: c-TACE, conventional transarterial chemoembolization; DEB-TACE, drug-eluting bead transarterial chemoembolization; DSM-TACE, degradable starch microspheres transarterial chemoembolization; B-TACE, balloon occluded transarterial chemoembolization; TAE, transarterial embolization; TARE, transarterial radioembolization

**Figure S10.13: Risk of bias contribution by intervention group in nausea, vomiting**

**Figure S10.14: Overall risk of bias by treatment comparison in nausea, vomiting**

Abbreviations: c-TACE, conventional transarterial chemoembolization; DEB-TACE, drug-eluting bead transarterial chemoembolization; DSM-TACE, degradable starch microspheres transarterial chemoembolization; B-TACE, balloon occluded transarterial chemoembolization; TAE, transarterial embolization; TARE, transarterial radioembolization

**Figure S10.15: Risk of bias contribution by intervention group in abdominal pain**

**Figure S10.16: Overall risk of bias by treatment comparison in abdominal pain**

Abbreviations: c-TACE, conventional transarterial chemoembolization; DEB-TACE, drug-eluting bead transarterial chemoembolization; DSM-TACE, degradable starch microspheres transarterial chemoembolization; B-TACE, balloon occluded transarterial chemoembolization; TAE, transarterial embolization; TARE, transarterial radioembolization

**Figure S10.17: Risk of bias contribution by intervention group in fever**

**Figure S10.18: Overall risk of bias by treatment comparison in fever**

Abbreviations: c-TACE, conventional transarterial chemoembolization; DEB-TACE, drug-eluting bead transarterial chemoembolization; DSM-TACE, degradable starch microspheres transarterial chemoembolization; B-TACE, balloon occluded transarterial chemoembolization; TAE, transarterial embolization; TARE, transarterial radioembolization

**Imprecision:** We use the CINeMA website to grade the accuracy of each comparison.

**Heterogeneity:** We assessed the degree of worry by comparing clinical reasoning based on 95% confidence intervals (CIs) while applying the same clinical reasoning framework as for inaccuracy. In particular, we judged the consistency of our findings based on the confidence and prediction intervals associated with clinically important effect sizes. And we used the same thresholds of clinical significance as described above and followed the recommendations automatically provided by CINeMA (https://cinema.ispm.unibe.ch/).

**Inconsistency:** For inconsistency, we looked at the results for node splitting (Appendix 6) and we saw major problems when p<0.10, but otherwise no problems.

**Table S10.1: CINeMA Results of objective response rate (ORR)**

| Comparison | Within-study bias | Reporting bias | Indirectness | Imprecision | Heterogeneity | Incoherence | Confidence rating |
| --- | --- | --- | --- | --- | --- | --- | --- |
| B-TACE:cTACE | Some concerns | Low risk | No concerns | No concerns | No concerns | Major concerns | Low |
| DEB-TACE:TAE | Some concerns | Low risk | No concerns | No concerns | Major concerns | No concerns | Low |
| DEB-TACE:TARE | Some concerns | Low risk | No concerns | Major concerns | No concerns | No concerns | Low |
| DEB-TACE:cTACE | Some concerns | Low risk | No concerns | No concerns | Major concerns | No concerns | Low |
| DSM-TACE:cTACE | Some concerns | Low risk | No concerns | No concerns | No concerns | Major concerns | Low |
| TAE:cTACE | No concerns | Low risk | No concerns | Major concerns | No concerns | No concerns | Low |
| TARE:cTACE | Some concerns | Low risk | No concerns | Major concerns | No concerns | No concerns | Low |
| B-TACE:DEB-TACE | Some concerns | Low risk | No concerns | No concerns | No concerns | Major concerns | Low |
| B-TACE:DSM-TACE | Some concerns | Low risk | No concerns | No concerns | No concerns | Major concerns | Low |
| B-TACE:TAE | Some concerns | Low risk | No concerns | No concerns | No concerns | Major concerns | Low |
| B-TACE:TARE | Some concerns | Low risk | No concerns | No concerns | No concerns | Major concerns | Low |
| DEB-TACE:DSM-TACE | Some concerns | Low risk | No concerns | No concerns | No concerns | Major concerns | Low |
| DSM-TACE:TAE | Some concerns | Low risk | No concerns | No concerns | No concerns | Major concerns | Low |
| DSM-TACE:TARE | Some concerns | Low risk | No concerns | No concerns | No concerns | Major concerns | Low |
| TAE:TARE | No concerns | Low risk | No concerns | Major concerns | No concerns | Major concerns | Low |

Abbreviations: c-TACE, conventional transarterial chemoembolization; DEB-TACE, drug-eluting bead transarterial chemoembolization; DSM-TACE, degradable starch microspheres transarterial chemoembolization; B-TACE, balloon occluded transarterial chemoembolization; TAE, transarterial embolization; TARE, transarterial radioembolization

**Table S10.2: CINeMA Results of disease control rate (DCR)**

| Comparison | Within-study bias | Reporting bias | Indirectness | Imprecision | Heterogeneity | Incoherence | Confidence rating |
| --- | --- | --- | --- | --- | --- | --- | --- |
| B-TACE:cTACE | Some concerns | Low risk | No concerns | No concerns | No concerns | Major concerns | Low |
| DEB-TACE:TAE | No concerns | Low risk | No concerns | No concerns | No concerns | No concerns | High |
| DEB-TACE:cTACE | Some concerns | Low risk | No concerns | No concerns | No concerns | No concerns | Moderate |
| DSM-TACE:cTACE | Some concerns | Low risk | No concerns | Major concerns | No concerns | Major concerns | Low |
| TAE:cTACE | No concerns | Low risk | No concerns | Major concerns | No concerns | No concerns | Low |
| TARE:cTACE | No concerns | Low risk | No concerns | Major concerns | No concerns | Major concerns | Low |
| B-TACE:DEB-TACE | Some concerns | Low risk | No concerns | Major concerns | No concerns | Major concerns | Low |
| B-TACE:DSM-TACE | Some concerns | Low risk | No concerns | Major concerns | No concerns | Major concerns | Low |
| B-TACE:TAE | Some concerns | Low risk | No concerns | No concerns | No concerns | Major concerns | Low |
| B-TACE:TARE | No concerns | Low risk | No concerns | No concerns | No concerns | Major concerns | Low |
| DEB-TACE:DSM-TACE | Some concerns | Low risk | No concerns | Major concerns | No concerns | Major concerns | Low |
| DEB-TACE:TARE | No concerns | Low risk | No concerns | No concerns | Major concerns | Major concerns | Low |
| DSM-TACE:TAE | Some concerns | Low risk | No concerns | Major concerns | No concerns | Major concerns | Low |
| DSM-TACE:TARE | No concerns | Low risk | No concerns | Major concerns | No concerns | Major concerns | Low |
| TAE:TARE | No concerns | Low risk | No concerns | Major concerns | No concerns | Major concerns | Low |

Abbreviations: c-TACE, conventional transarterial chemoembolization; DEB-TACE, drug-eluting bead transarterial chemoembolization; DSM-TACE, degradable starch microspheres transarterial chemoembolization; B-TACE, balloon occluded transarterial chemoembolization; TAE, transarterial embolization; TARE, transarterial radioembolization

**Table S10.3: CINeMA Results of complete response (CR)**

| Comparison | Within-study bias | Reporting bias | Indirectness | Imprecision | Heterogeneity | Incoherence | Confidence rating |
| --- | --- | --- | --- | --- | --- | --- | --- |
| B-TACE:cTACE | No concerns | Low risk | No concerns | Major concerns | No concerns | No concerns | Low |
| DEB-TACE:TAE | No concerns | Low risk | No concerns | Major concerns | No concerns | No concerns | Low |
| DEB-TACE:cTACE | Some concerns | Low risk | No concerns | No concerns | No concerns | No concerns | Moderate |
| DSM-TACE:cTACE | Some concerns | Low risk | No concerns | Major concerns | No concerns | No concerns | Low |
| TAE:cTACE | No concerns | Low risk | No concerns | Major concerns | No concerns | No concerns | Low |
| TARE:cTACE | No concerns | Low risk | No concerns | Major concerns | No concerns | No concerns | Low |
| B-TACE:DEB-TACE | Some concerns | Low risk | No concerns | Major concerns | No concerns | No concerns | Low |
| B-TACE:DSM-TACE | Some concerns | Low risk | No concerns | Major concerns | No concerns | No concerns | Low |
| B-TACE:TAE | No concerns | Low risk | No concerns | Major concerns | No concerns | No concerns | Low |
| B-TACE:TARE | No concerns | Low risk | No concerns | Major concerns | No concerns | No concerns | Low |
| DEB-TACE:DSM-TACE | Some concerns | Low risk | No concerns | Major concerns | No concerns | No concerns | Low |
| DEB-TACE:TARE | No concerns | Low risk | No concerns | Major concerns | No concerns | No concerns | Low |
| DSM-TACE:TAE | Some concerns | Low risk | No concerns | Major concerns | No concerns | No concerns | Low |
| DSM-TACE:TARE | No concerns | Low risk | No concerns | Major concerns | No concerns | No concerns | Low |
| TAE:TARE | No concerns | Low risk | No concerns | Major concerns | No concerns | No concerns | Low |

Abbreviations: c-TACE, conventional transarterial chemoembolization; DEB-TACE, drug-eluting bead transarterial chemoembolization; DSM-TACE, degradable starch microspheres transarterial chemoembolization; B-TACE, balloon occluded transarterial chemoembolization; TAE, transarterial embolization; TARE, transarterial radioembolization

**Table S10.4: CINeMA Results of partial response (PR)**

| Comparison | Within-study bias | Reporting bias | Indirectness | Imprecision | Heterogeneity | Incoherence | Confidence rating |
| --- | --- | --- | --- | --- | --- | --- | --- |
| B-TACE:cTACE | Some concerns | Low risk | No concerns | No concerns | No concerns | No concerns | Moderate |
| DEB-TACE:TAE | Some concerns | Low risk | No concerns | No concerns | No concerns | No concerns | Moderate |
| DEB-TACE:cTACE | Some concerns | Low risk | No concerns | No concerns | No concerns | No concerns | Moderate |
| DSM-TACE:cTACE | Some concerns | Low risk | No concerns | Major concerns | No concerns | No concerns | Low |
| TAE:cTACE | No concerns | Low risk | No concerns | Major concerns | No concerns | No concerns | Low |
| TARE:cTACE | Some concerns | Low risk | No concerns | Major concerns | No concerns | No concerns | Low |
| B-TACE:DEB-TACE | Some concerns | Low risk | No concerns | No concerns | No concerns | No concerns | Moderate |
| B-TACE:DSM-TACE | Some concerns | Low risk | No concerns | Major concerns | No concerns | No concerns | Low |
| B-TACE:TAE | Some concerns | Low risk | No concerns | No concerns | No concerns | No concerns | Moderate |
| B-TACE:TARE | Some concerns | Low risk | No concerns | No concerns | No concerns | No concerns | Moderate |
| DEB-TACE:DSM-TACE | Some concerns | Low risk | No concerns | Major concerns | No concerns | No concerns | Low |
| DEB-TACE:TARE | Some concerns | Low risk | No concerns | Major concerns | No concerns | No concerns | Low |
| DSM-TACE:TAE | Some concerns | Low risk | No concerns | Major concerns | No concerns | No concerns | Low |
| DSM-TACE:TARE | Some concerns | Low risk | No concerns | Major concerns | No concerns | No concerns | Low |
| TAE:TARE | No concerns | Low risk | No concerns | Major concerns | No concerns | No concerns | Low |

Abbreviations: c-TACE, conventional transarterial chemoembolization; DEB-TACE, drug-eluting bead transarterial chemoembolization; DSM-TACE, degradable starch microspheres transarterial chemoembolization; B-TACE, balloon occluded transarterial chemoembolization; TAE, transarterial embolization; TARE, transarterial radioembolization

**Table S10.5: CINeMA Results of stable disease (SD)**

| Comparison | Within-study bias | Reporting bias | Indirectness | Imprecision | Heterogeneity | Incoherence | Confidence rating |
| --- | --- | --- | --- | --- | --- | --- | --- |
| B-TACE:cTACE | Some concerns | Low risk | No concerns | Major concerns | No concerns | No concerns | Low |
| DEB-TACE:TAE | Some concerns | Low risk | No concerns | Major concerns | No concerns | No concerns | Low |
| DEB-TACE:cTACE | Some concerns | Low risk | No concerns | No concerns | Major concerns | Major concerns | Low |
| DSM-TACE:cTACE | Some concerns | Low risk | No concerns | Major concerns | No concerns | No concerns | Low |
| TAE:cTACE | No concerns | Low risk | No concerns | Major concerns | No concerns | Major concerns | Low |
| TARE:cTACE | No concerns | Low risk | No concerns | Major concerns | No concerns | No concerns | Low |
| B-TACE:DEB-TACE | Some concerns | Low risk | No concerns | Major concerns | No concerns | No concerns | Low |
| B-TACE:DSM-TACE | Some concerns | Low risk | No concerns | Major concerns | No concerns | No concerns | Low |
| B-TACE:TAE | Some concerns | Low risk | No concerns | Major concerns | No concerns | No concerns | Low |
| B-TACE:TARE | No concerns | Low risk | No concerns | Major concerns | No concerns | No concerns | Low |
| DEB-TACE:DSM-TACE | Some concerns | Low risk | No concerns | Major concerns | No concerns | No concerns | Low |
| DEB-TACE:TARE | No concerns | Low risk | No concerns | Major concerns | No concerns | No concerns | Low |
| DSM-TACE:TAE | Some concerns | Low risk | No concerns | Major concerns | No concerns | No concerns | Low |
| DSM-TACE:TARE | No concerns | Low risk | No concerns | Major concerns | No concerns | No concerns | Low |
| TAE:TARE | No concerns | Low risk | No concerns | Major concerns | No concerns | No concerns | Low |

Abbreviations: c-TACE, conventional transarterial chemoembolization; DEB-TACE, drug-eluting bead transarterial chemoembolization; DSM-TACE, degradable starch microspheres transarterial chemoembolization; B-TACE, balloon occluded transarterial chemoembolization; TAE, transarterial embolization; TARE, transarterial radioembolization

**Table S10.6: CINeMA Results of progressive disease (PD)**

| Comparison | Within-study bias | Reporting bias | Indirectness | Imprecision | Heterogeneity | Incoherence | Confidence rating |
| --- | --- | --- | --- | --- | --- | --- | --- |
| B-TACE:cTACE | Some concerns | Low risk | No concerns | No concerns | No concerns | No concerns | Moderate |
| DEB-TACE:TAE | No concerns | Low risk | No concerns | No concerns | No concerns | No concerns | High |
| DEB-TACE:TARE | Some concerns | Low risk | No concerns | Major concerns | No concerns | No concerns | Low |
| DEB-TACE:cTACE | Some concerns | Low risk | No concerns | No concerns | No concerns | No concerns | Low |
| DSM-TACE:cTACE | Some concerns | Low risk | No concerns | Major concerns | No concerns | No concerns | Low |
| TAE:cTACE | No concerns | Low risk | No concerns | No concerns | No concerns | No concerns | High |
| TARE:cTACE | No concerns | Low risk | No concerns | Major concerns | No concerns | Major concerns | Low |
| B-TACE:DEB-TACE | Some concerns | Low risk | No concerns | Major concerns | No concerns | No concerns | Low |
| B-TACE:DSM-TACE | Some concerns | Low risk | No concerns | Major concerns | No concerns | No concerns | Low |
| B-TACE:TAE | No concerns | Low risk | No concerns | No concerns | No concerns | No concerns | High |
| B-TACE:TARE | No concerns | Low risk | No concerns | Major concerns | No concerns | No concerns | Low |
| DEB-TACE:DSM-TACE | Some concerns | Low risk | No concerns | Major concerns | No concerns | No concerns | Low |
| DSM-TACE:TAE | Some concerns | Low risk | No concerns | Major concerns | No concerns | No concerns | Low |
| DSM-TACE:TARE | Some concerns | Low risk | No concerns | Major concerns | No concerns | No concerns | Low |
| TAE:TARE | No concerns | Low risk | No concerns | Major concerns | No concerns | No concerns | Low |

Abbreviations: c-TACE, conventional transarterial chemoembolization; DEB-TACE, drug-eluting bead transarterial chemoembolization; DSM-TACE, degradable starch microspheres transarterial chemoembolization; B-TACE, balloon occluded transarterial chemoembolization; TAE, transarterial embolization; TARE, transarterial radioembolization

**Table S10.7: CINeMA Results of nausea, vomiting**

| Comparison | Within-study bias | Reporting bias | Indirectness | Imprecision | Heterogeneity | Incoherence | Confidence rating |
| --- | --- | --- | --- | --- | --- | --- | --- |
| DEB-TACE:TAE | No concerns | Low risk | No concerns | No concerns | No concerns | No concerns | High |
| DEB-TACE:cTACE | Some concerns | Low risk | No concerns | No concerns | Major concerns | No concerns | Low |
| DSM-TACE:cTACE | Some concerns | Low risk | No concerns | Major concerns | No concerns | No concerns | Low |
| TAE:cTACE | No concerns | Low risk | No concerns | No concerns | No concerns | No concerns | High |
| DEB-TACE:DSM-TACE | Some concerns | Low risk | No concerns | Major concerns | No concerns | No concerns | Low |
| DSM-TACE:TAE | Some concerns | Low risk | No concerns | Major concerns | No concerns | No concerns | Low |

Abbreviations: c-TACE, conventional transarterial chemoembolization; DEB-TACE, drug-eluting bead transarterial chemoembolization; DSM-TACE, degradable starch microspheres transarterial chemoembolization; B-TACE, balloon occluded transarterial chemoembolization; TAE, transarterial embolization; TARE, transarterial radioembolization

**Table S10.8: CINeMA Results of abdominal pain**

| Comparison | Within-study bias | Reporting bias | Indirectness | Imprecision | Heterogeneity | Incoherence | Confidence rating |
| --- | --- | --- | --- | --- | --- | --- | --- |
| B-TACE:cTACE | No concerns | Low risk | No concerns | Major concerns | No concerns | Major concerns | Low |
| DEB-TACE:TAE | No concerns | Low risk | No concerns | Major concerns | No concerns | No concerns | Low |
| DEB-TACE:cTACE | Some concerns | Low risk | No concerns | No concerns | Major concerns | No concerns | Low |
| DSM-TACE:cTACE | Some concerns | Low risk | No concerns | Major concerns | No concerns | Major concerns | Low |
| TAE:cTACE | No concerns | Low risk | No concerns | Major concerns | No concerns | No concerns | Low |
| TARE:cTACE | No concerns | Low risk | No concerns | Major concerns | No concerns | Major concerns | Low |
| B-TACE:DEB-TACE | No concerns | Low risk | No concerns | Major concerns | No concerns | Major concerns | Low |
| B-TACE:DSM-TACE | No concerns | Low risk | No concerns | Major concerns | No concerns | Major concerns | Low |
| B-TACE:TAE | No concerns | Low risk | No concerns | Major concerns | No concerns | Major concerns | Low |
| B-TACE:TARE | No concerns | Low risk | No concerns | Major concerns | No concerns | Major concerns | Low |
| DEB-TACE:DSM-TACE | Some concerns | Low risk | No concerns | Major concerns | No concerns | Major concerns | Low |
| DEB-TACE:TARE | No concerns | Low risk | No concerns | Major concerns | No concerns | Major concerns | Low |
| DSM-TACE:TAE | Some concerns | Low risk | No concerns | Major concerns | No concerns | Major concerns | Low |
| DSM-TACE:TARE | No concerns | Low risk | No concerns | Major concerns | No concerns | Major concerns | Low |
| TAE:TARE | No concerns | Low risk | No concerns | Major concerns | No concerns | Major concerns | Low |

Abbreviations: c-TACE, conventional transarterial chemoembolization; DEB-TACE, drug-eluting bead transarterial chemoembolization; DSM-TACE, degradable starch microspheres transarterial chemoembolization; B-TACE, balloon occluded transarterial chemoembolization; TAE, transarterial embolization; TARE, transarterial radioembolization

**Table S10.9: CINeMA Results of fever**

| Comparison | Within-study bias | Reporting bias | Indirectness | Imprecision | Heterogeneity | Incoherence | Confidence rating |
| --- | --- | --- | --- | --- | --- | --- | --- |
| B-TACE:cTACE | No concerns | Low risk | No concerns | Major concerns | No concerns | No concerns | Low |
| DEB-TACE:TAE | No concerns | Low risk | No concerns | No concerns | No concerns | No concerns | High |
| DEB-TACE:cTACE | Some concerns | Low risk | No concerns | No concerns | No concerns | No concerns | Moderate |
| DSM-TACE:cTACE | Some concerns | Low risk | No concerns | No concerns | No concerns | No concerns | Moderate |
| TAE:cTACE | No concerns | Low risk | No concerns | Major concerns | No concerns | No concerns | Low |
| TARE:cTACE | No concerns | Low risk | No concerns | Major concerns | No concerns | No concerns | Low |
| B-TACE:DEB-TACE | Some concerns | Low risk | No concerns | Major concerns | No concerns | No concerns | Low |
| B-TACE:DSM-TACE | Some concerns | Low risk | No concerns | Major concerns | No concerns | No concerns | Low |
| B-TACE:TAE | No concerns | Low risk | No concerns | No concerns | No concerns | No concerns | High |
| B-TACE:TARE | No concerns | Low risk | No concerns | Major concerns | No concerns | No concerns | Low |
| DEB-TACE:DSM-TACE | Some concerns | Low risk | No concerns | No concerns | No concerns | No concerns | Moderate |
| DEB-TACE:TARE | No concerns | Low risk | No concerns | Major concerns | No concerns | No concerns | Low |
| DSM-TACE:TAE | Some concerns | Low risk | No concerns | No concerns | No concerns | No concerns | Moderate |
| DSM-TACE:TARE | No concerns | Low risk | No concerns | Major concerns | No concerns | No concerns | Low |
| TAE:TARE | No concerns | Low risk | No concerns | Major concerns | No concerns | No concerns | Low |

Abbreviations: c-TACE, conventional transarterial chemoembolization; DEB-TACE, drug-eluting bead transarterial chemoembolization; DSM-TACE, degradable starch microspheres transarterial chemoembolization; B-TACE, balloon occluded transarterial chemoembolization; TAE, transarterial embolization; TARE, transarterial radioembolization

**Appendix 11: Funnel plots**

**Figure S11.1: Funnel plot of objective response rate (ORR)**

**Figure S11.2: Funnel plot of disease control rate (DCR)**

**Figure S11.3: Funnel plot of complete response (CR)**

**Figure S11.4: Funnel plot of partial response (PR)**

**Figure S11.5: Funnel plot of stable disease (SD)**

**Figure S11.6: Funnel plot of progressive disease (PD)**

**Figure S11.7: Funnel plot of nausea, vomiting**

**Figure S11.8: Funnel plot of abdominal pain**

**Figure S11.9: Funnel plot of fever**

**Appendix 12: Summary of Adverse Events**

**Table S12.1. Summary of postembolization syndrome reported in the included comparative studies**

| **First author (Year)** | **Intervention measures (Test/Control)** | **Nausea, Vomiting, N (%)** | | | | **Abdominal pain, N (%)** | | | | **Fever, N (%)** | | | |
| --- | --- | --- | --- | --- | --- | --- | --- | --- | --- | --- | --- | --- | --- |
|  |  | **Test** | **Total** | **Control** | **Total** | **Test** | **Total** | **Control** | **Total** | **Test** | **Total** | **Control** | **Total** |
| Hannah van Malenstein (2011) | DEB-TACE vs cTACE | 2 | 13 | 2 | 12 |  |  |  |  | 1 | 13 | 5 | 12 |
| Shi Z X (2023) | DEB-TACE vs cTACE | 10 | 45 | 12 | 45 | 42 | 45 | 43 | 45 | 23 | 45 | 13 | 45 |
| Shan L T (2026) | B-TACE vs cTACE |  |  |  |  | 9 | 29 | 17 | 29 | 3 | 29 | 9 | 29 |
| Jin B X (2024) | DEB-TACE vs cTACE |  |  |  |  |  |  |  |  |  |  |  |  |
| T.J.Vogl (2021) | DSM-TACE vs cTACE |  |  |  |  |  |  |  |  |  |  |  |  |
| Johannes Lammer (2009) | DEB-TACE vs cTACE |  |  |  |  |  |  |  |  |  |  |  |  |
| R Golfieri (2014) | DEB-TACE vs cTACE | 2 | 89 | 3 | 88 | 22 | 89 | 63 | 88 | 7 | 89 | 10 | 88 |
| Aleksandar Gjoreski (2020) | DSM-TACE vs cTACE | 20 | 32 | 19 | 28 | 12 | 32 | 16 | 28 | 13 | 32 | 23 | 28 |
| Duan X H (2024) | DEB-TACE vs cTACE | 24 | 102 | 46 | 105 | 49 | 102 | 55 | 105 | 48 | 102 | 62 | 105 |
| Li K F (2023) | DEB-TACE vs cTACE | 2 | 52 | 3 | 52 | 1 | 52 | 1 | 52 | 2 | 52 | 3 | 52 |
| Shi J (2022) | DEB-TACE vs cTACE | 14 | 50 | 16 | 50 | 33 | 50 | 37 | 50 | 16 | 50 | 23 | 50 |
| Chen P (2021) | DEB-TACE vs cTACE |  |  |  |  | 7 | 40 | 9 | 40 | 12 | 40 | 23 | 40 |
| Chen J X (2025) | DEB-TACE vs cTACE | 1 | 40 | 3 | 40 | 2 | 40 | 4 | 40 | 2 | 40 | 4 | 40 |
| Huang Z (2020) | DEB-TACE vs cTACE | 80 | 156 | 75 | 152 | 41 | 156 | 43 | 152 | 70 | 156 | 73 | 152 |
| Zhang D Y (2021) | DEB-TACE vs cTACE | 7 | 36 | 13 | 36 | 2 | 36 | 4 | 36 | 2 | 36 | 7 | 36 |
| Zhang C (2024) | DEB-TACE vs cTACE | 1 | 46 | 5 | 46 |  |  |  |  | 2 | 46 | 3 | 46 |
| Chi Q (2018) | DEB-TACE vs cTACE |  |  |  |  |  |  |  |  |  |  |  |  |
| Wang H P (2018) | DEB-TACE vs cTACE | 32 | 60 | 39 | 69 | 29 | 60 | 34 | 69 | 22 | 60 | 31 | 69 |
| Wang Z (2019) | DEB-TACE vs cTACE | 13 | 45 | 12 | 45 | 6 | 45 | 4 | 45 | 4 | 45 | 5 | 45 |
| Zeng Q S (2024) | DEB-TACE vs cTACE | 15 | 48 | 36 | 48 | 14 | 48 | 29 | 48 | 16 | 48 | 21 | 48 |
| Zhang N (2022) | DEB-TACE vs cTACE |  |  |  |  |  |  |  |  |  |  |  |  |
| Chen Y (2017) | DEB-TACE vs cTACE |  |  |  |  |  |  |  |  |  |  |  |  |
| Lu Y (2023) | DEB-TACE vs cTACE | 2 | 35 | 3 | 35 | 1 | 35 | 2 | 35 | 3 | 35 | 2 | 35 |
| Geng J Z(2022) | DEB-TACE vs cTACE | 1 | 29 | 1 | 29 | 1 | 29 | 1 | 29 | 1 | 29 | 2 | 29 |
| Li Y F(2020) | DEB-TACE vs cTACE |  |  |  |  |  |  |  |  |  |  |  |  |
| Wang X L (2023) | DEB-TACE vs cTACE |  |  |  |  |  |  |  |  |  |  |  |  |
| Zhang J (2022) | DEB-TACE vs cTACE | 0 | 46 | 3 | 46 | 0 | 46 | 2 | 46 |  |  |  |  |
| Zhao P (2022) | DEB-TACE vs cTACE | 7 | 40 | 11 | 40 |  |  |  |  |  |  |  |  |
| Xu R L (2023) | DEB-TACE vs cTACE |  |  |  |  |  |  |  |  |  |  |  |  |
| Zhang M X (2022) | DEB-TACE vs cTACE | 5 | 48 | 8 | 48 | 20 | 48 | 18 | 48 | 9 | 48 | 13 | 48 |
| Zhou L J (2023) | DEB-TACE vs cTACE |  |  |  |  |  |  |  |  |  |  |  |  |
| Li X D (2025) | DEB-TACE vs cTACE |  |  |  |  | 1 | 30 | 1 | 30 |  |  |  |  |
| Dai S B (2022) | DEB-TACE vs cTACE | 1 | 67 | 7 | 67 | 1 | 67 | 4 | 67 | 2 | 67 | 6 | 67 |
| Pang H C (2022) | DEB-TACE vs cTACE | 1 | 30 | 2 | 30 |  |  |  |  | 1 | 30 | 2 | 30 |
| Li H B (2025) | DEB-TACE vs cTACE | 10 | 61 | 8 | 61 |  |  |  |  | 3 | 61 | 1 | 61 |
| Peng C Y (2020) | DEB-TACE vs cTACE | 42 | 60 | 46 | 60 | 54 | 60 | 55 | 60 | 58 | 60 | 59 | 60 |
| Jiang F S (2021) | DEB-TACE vs cTACE | 2 | 30 | 1 | 30 | 1 | 30 | 2 | 30 | 0 | 30 | 1 | 30 |
| Zhao L (2020) | DEB-TACE vs cTACE | 3 | 30 | 4 | 30 | 1 | 30 | 2 | 30 |  |  |  |  |
| Zhao W (2020) | DEB-TACE vs cTACE |  |  |  |  |  |  |  |  |  |  |  |  |
| Josep M Llovet (2002) | TAE vs cTACE |  |  |  |  |  |  |  |  |  |  |  |  |
| T Meyer (2013) | DEB-TACE vs TAE | 4 | 43 | 1 | 38 | 12 | 43 | 8 | 38 | 1 | 43 | 1 | 38 |
| Riad Salem (2016) | Y90（TARE） vs cTACE |  |  |  |  |  |  |  |  |  |  |  |  |
| Frank T. Kolligs (2015) | Y90（TARE） vs cTACE |  |  |  |  |  |  |  |  | 0 | 13 | 1 | 15 |
| Saburo Kawail (1992) | TAE vs cTACE |  |  |  |  | 74 | 115 | 87 | 134 | 92 | 115 | 108 | 134 |
| Katerina Malagari (2010) | DEB-TACE vs TAE |  |  |  |  |  |  |  |  |  |  |  |  |
| Karen T. Brown (2016) | DEB-TACE vs TAE |  |  |  |  |  |  |  |  |  |  |  |  |
| Hironobu Ihira (2023) | TAE vs cTACE |  |  |  |  |  |  |  |  |  |  |  |  |
| Elisabeth Dhondt (2021) | DEB-TACE vs Y90（TARE） |  |  |  |  |  |  |  |  |  |  |  |  |
| JEAN-LUC RAOUL (1997) | I131（TARE） vs cTACE |  |  |  |  | 0 | 65 | 7 | 64 | 1 | 65 | 0 | 64 |
| Simon Chun Ho Yu (2014) | TAE vs cTACE | 6 | 45 | 21 | 45 | 33 | 45 | 25 | 45 | 33 | 45 | 22 | 45 |
| Rodolfo Sacco (2011) | DEB-TACE vs cTACE |  |  |  |  |  |  |  |  |  |  |  |  |
| Michael B. Pitton (2014) | DEB-TACE vs Y90（TARE） |  |  |  |  |  |  |  |  |  |  |  |  |
| Yang Y B (2012) | B-TACE vs cTACE |  |  |  |  |  |  |  |  |  |  |  |  |
| Cheng H Y (2004) | B-TACE vs cTACE |  |  |  |  |  |  |  |  | 34 | 34 | 40 | 40 |
| Ma M (2019) | TAE vs cTACE |  |  |  |  | 2 | 34 | 1 | 34 | 2 | 34 | 1 | 34 |
| Feng T (2020) | TAE vs cTACE |  |  |  |  |  |  |  |  |  |  |  |  |

Abbreviations: c-TACE, conventional transarterial chemoembolization; DEB-TACE, drug-eluting bead transarterial chemoembolization; DSM-TACE, degradable starch microspheres transarterial chemoembolization; B-TACE, balloon occluded transarterial chemoembolization; TAE, transarterial embolization; TARE, transarterial radioembolization

**Appendix 13: Sensitivity Analysis**

**Table S13.1:Sensitivity analysis restricted to trials at low overall risk of bias**

**Note:** Sensitivity analysis was restricted to randomized controlled trials judged as having low overall risk of bias using RoB 2.0. Among the 56 included trials, 10 were rated as low risk and 46 as having some concerns; no trial was rated as high risk of bias. Because DSM-TACE was not represented in the low-risk-only networks and several treatment nodes were sparsely connected, sensitivity analyses were interpreted together with global heterogeneity and node-splitting diagnostics rather than as a replacement for the primary network model.

| **Outcome** | **Main analysis: studies / arms / participants** | **Treatment nodes in main analysis** | **Low-risk-only sensitivity analysis: studies / arms** | **Treatment nodes retained in sensitivity analysis** | **Global P value** | **τ² network** | **Minimum node-splitting P value** |
| --- | --- | --- | --- | --- | --- | --- | --- |
| ORR | 48 / 96 / 4282 | cTACE, DEB-TACE, DSM-TACE, B-TACE, TAE, TARE | 10 / 20 | cTACE, DEB-TACE, B-TACE, TAE, TARE | 0.9513 | 0.0153 | 0.754 |
| DCR | 44 / 88 / 3999 | cTACE, DEB-TACE, DSM-TACE, B-TACE, TAE, TARE | 9 / 18 | cTACE, DEB-TACE, B-TACE, TAE, TARE | 0.6459 | 0.0028 | 0.646 |
| CR | 46 / 92 / 4156 | cTACE, DEB-TACE, DSM-TACE, B-TACE, TAE, TARE | 10 / 20 | cTACE, DEB-TACE, B-TACE, TAE, TARE | 0.9091 | 0.0355 | 0.909 |
| PR | 49 / 98 / 4376 | cTACE, DEB-TACE, DSM-TACE, B-TACE, TAE, TARE | 10 / 20 | cTACE, DEB-TACE, B-TACE, TAE, TARE | 0.9481 | <0.001 | 0.948 |
| SD | 45 / 90 / 4024 | cTACE, DEB-TACE, DSM-TACE, B-TACE, TAE, TARE | 9 / 18 | cTACE, DEB-TACE, B-TACE, TAE, TARE | 0.0843 | 0.0170 | 0.841 |
| PD | 46 / 92 / 4048 | cTACE, DEB-TACE, DSM-TACE, B-TACE, TAE, TARE | 9 / 18 | cTACE, DEB-TACE, B-TACE, TAE, TARE | 0.1250 | 0.0429 | 0.175 |
| Nausea, vomiting | 27 / 54 / 2753 | cTACE, DEB-TACE, DSM-TACE, TAE | 4 / 8 | cTACE, DEB-TACE, TAE | 0.9666 | 0.1245 | 0.967 |
| Abdominal pain | 28 / 56 / 3018 | cTACE, DEB-TACE, DSM-TACE, B-TACE, TAE, TARE | 7 / 14 | cTACE, DEB-TACE, B-TACE, TAE, TARE | 0.1610 | 0.2819 | 0.161 |
| Fever | 31 / 62 / 3207 | cTACE, DEB-TACE, DSM-TACE, B-TACE, TAE, TARE | 8 / 16 | cTACE, DEB-TACE, B-TACE, TAE, TARE | 0.6538 | 0.0713 | 0.654 |

Abbreviations: ORR, objective response rate; DCR, disease control rate; CR, complete response; PR, partial response; SD, stable disease; PD, progressive disease; cTACE, conventional transarterial chemoembolization; DEB-TACE, drug-eluting bead transarterial chemoembolization; DSM-TACE, degradable starch microspheres transarterial chemoembolization; B-TACE, balloon-occluded transarterial chemoembolization; TAE, transarterial embolization; TARE, transarterial radioembolization.

**Table S13.2: Sensitivity analysis excluding sparsely connected treatment nodes**

**Note:** Sparse treatment nodes were defined as non-cTACE interventions supported by two or fewer studies for a given outcome. This analysis was used to assess whether the primary conclusions were mainly driven by weakly connected treatment nodes.

| **Outcome** | **Main analysis: studies / arms / participants** | **Sparse treatment nodes excluded** | **Core-network sensitivity analysis: studies / arms / participants** | **Treatment nodes retained** |
| --- | --- | --- | --- | --- |
| ORR | 48 / 96 / 4282 | DSM-TACE | 48 / 95 / 4254 | cTACE, DEB-TACE, B-TACE, TAE, TARE |
| DCR | 44 / 88 / 3999 | DSM-TACE, TARE | 44 / 85 / 3933 | cTACE, DEB-TACE, B-TACE, TAE |
| CR | 46 / 92 / 4156 | DSM-TACE, TARE | 46 / 89 / 4090 | cTACE, DEB-TACE, B-TACE, TAE |
| PR | 49 / 98 / 4376 | DSM-TACE | 49 / 97 / 4348 | cTACE, DEB-TACE, B-TACE, TAE, TARE |
| SD | 45 / 90 / 4024 | DSM-TACE, TARE | 45 / 87 / 3958 | cTACE, DEB-TACE, B-TACE, TAE |
| PD | 46 / 92 / 4048 | DSM-TACE | 46 / 91 / 4020 | cTACE, DEB-TACE, B-TACE, TAE, TARE |
| Nausea, vomiting | 27 / 54 / 2753 | DSM-TACE, TAE | 27 / 51 / 2638 | cTACE, DEB-TACE |
| Abdominal pain | 28 / 56 / 3018 | DSM-TACE, B-TACE, TARE | 28 / 53 / 2892 | cTACE, DEB-TACE, TAE |
| Fever | 31 / 62 / 3207 | DSM-TACE, B-TACE, TARE | 31 / 57 / 3034 | cTACE, DEB-TACE, TAE |

Abbreviations: ORR, objective response rate; DCR, disease control rate; CR, complete response; PR, partial response; SD, stable disease; PD, progressive disease; cTACE, conventional transarterial chemoembolization; DEB-TACE, drug-eluting bead transarterial chemoembolization; DSM-TACE, degradable starch microspheres transarterial chemoembolization; B-TACE, balloon-occluded transarterial chemoembolization; TAE, transarterial embolization; TARE, transarterial radioembolization.

**Table S13.3: Sensitivity analysis based on heterogeneity and inconsistency diagnostics**

**Note:** A P value <0.10 was considered suggestive of potential inconsistency. The sensitivity interpretation was based on the combined assessment of global consistency, local node-splitting, and network heterogeneity.

| **Outcome** | **Global P value** | **τ² network** | **Minimum node-splitting P value** |
| --- | --- | --- | --- |
| ORR | 0.9513 | 0.0153 | 0.754 |
| DCR | 0.6459 | 0.0028 | 0.646 |
| CR | 0.9091 | 0.0355 | 0.909 |
| PR | 0.9481 | <0.001 | 0.948 |
| SD | 0.0843 | 0.0170 | 0.841 |
| PD | 0.1250 | 0.0429 | 0.175 |
| Nausea, vomiting | 0.9666 | 0.1245 | 0.967 |
| Abdominal pain | 0.1610 | 0.2819 | 0.161 |
| Fever | 0.6538 | 0.0713 | 0.654 |

Abbreviations: ORR, objective response rate; DCR, disease control rate; CR, complete response; PR, partial response; SD, stable disease; PD, progressive disease.

**Table S13.4: Sensitivity analysis excluding studies with “some concerns” in key RoB 2 domains**

**Note:** Key RoB 2 domains were defined as D2, D4, and D5, corresponding to bias due to deviations from intended interventions, bias in measurement of the outcome, and bias in selection of the reported result. Studies rated as having “some concerns” in any of these key domains were excluded. This analysis was exploratory because it substantially reduced the number of available trials and removed DSM-TACE from all sensitivity networks.

| **Outcome** | **Main analysis: studies / arms** | **Sensitivity analysis: studies / arms** | **Treatment nodes retained** |
| --- | --- | --- | --- |
| ORR | 48 / 96 | 12 / 24 | cTACE, DEB-TACE, B-TACE, TAE, TARE |
| DCR | 44 / 88 | 9 / 18 | cTACE, DEB-TACE, B-TACE, TAE, TARE |
| CR | 46 / 92 | 10 / 20 | cTACE, DEB-TACE, B-TACE, TAE, TARE |
| PR | 49 / 98 | 13 / 26 | cTACE, DEB-TACE, B-TACE, TAE, TARE |
| SD | 45 / 90 | 9 / 18 | cTACE, DEB-TACE, B-TACE, TAE, TARE |
| PD | 46 / 92 | 10 / 20 | cTACE, DEB-TACE, B-TACE, TAE, TARE |
| Nausea, vomiting | 27 / 54 | 4 / 8 | cTACE, DEB-TACE, TAE |
| Abdominal pain | 28 / 56 | 7 / 14 | cTACE, DEB-TACE, B-TACE, TAE, TARE |
| Fever | 31 / 62 | 8 / 16 | cTACE, DEB-TACE, B-TACE, TAE, TARE |

Abbreviations: ORR, objective response rate; DCR, disease control rate; CR, complete response; PR, partial response; SD, stable disease; PD, progressive disease; cTACE, conventional transarterial chemoembolization; DEB-TACE, drug-eluting bead transarterial chemoembolization; DSM-TACE, degradable starch microspheres transarterial chemoembolization; B-TACE, balloon-occluded transarterial chemoembolization; TAE, transarterial embolization; TARE, transarterial radioembolization.

**Table S13.5: Sensitivity analyses for objective response rate**

**Note:** RR >1 favours the listed active treatment for ORR. NE, not estimable. Sensitivity estimates were obtained with a common random-effects contrast framework and are exploratory. The exclusion analysis leaves one contemporary HCC-specific B-TACE trial; its large, imprecise estimate demonstrates fragility and should not be interpreted as confirmation of superiority.

| **Analysis** | **DEB-TACE vs cTACE RR**  **(95% CI)** | **DSM-TACE vs cTACE RR**  **(95% CI)** | **B-TACE vs cTACE RR**  **(95% CI)** | **TAE vs cTACE RR**  **(95% CI)** | **TARE vs cTACE RR**  **(95% CI)** |
| --- | --- | --- | --- | --- | --- |
| Primary network | 1.36 (1.27-1.47) | 0.37 (0.21-0.63) | 3.50 (1.92-6.36) | 0.96 (0.81-1.15) | 1.23 (0.98-1.53) |
| Published 2010 onward | 1.38 (1.30-1.46) | 0.36 (0.22-0.59) | 6.80 (2.60-17.80) | 0.95 (0.79-1.13) | 1.28 (1.10-1.48) |
| English-language reports | 1.34 (1.15-1.57) | 0.36 (0.21-0.61) | 6.33 (2.06-19.50) | 0.98 (0.83-1.17) | 1.22 (0.98-1.51) |
| Overall low risk of bias | 1.30 (1.05-1.59) | NA | 6.33 (2.04-19.62) | 1.04 (0.84-1.30) | 1.17 (0.81-1.69) |
| Exclude two indirect hypovascular/mixed-tumor B-TACE trials | 1.36 (1.29-1.43) | 0.36 (0.22-0.59) | 6.33 (2.09-19.17) | 0.96 (0.84-1.10) | 1.24 (1.12-1.37) |

**Appendix 14: Regression Analysis**

**Table S14.1: Meta-regression analysis of potential effect modifiers for main outcomes**

**Note:** Regression analyses were exploratory and based on study-level covariates. Each covariate was assessed in a separate univariable model adjusted for treatment comparison. The coefficients are expressed on the log odds ratio scale. Because several covariates had missing data and some treatment nodes were sparsely connected, these results should be interpreted as hypothesis-generating rather than confirmatory.

| **Covariate** | **ORR coefficient** | **95% CI** | **P value** | **DCR coefficient** | **95% CI** | **P value** |
| --- | --- | --- | --- | --- | --- | --- |
| Mean age | -0.445 | -0.691 to -0.198 | <0.001 | -0.487 | -0.753 to -0.220 | <0.001 |
| Male proportion | -0.007 | -0.135 to 0.121 | 0.916 | -0.075 | -0.218 to 0.068 | 0.304 |
| BCLC C proportion | 0.084 | -0.047 to 0.216 | 0.208 | 0.094 | -0.018 to 0.207 | 0.101 |
| Child-Pugh A proportion | -0.078 | -0.182 to 0.025 | 0.139 | -0.142 | -0.249 to -0.034 | 0.010 |
| Maximum tumor diameter | -0.006 | -0.135 to 0.123 | 0.924 | 0.035 | -0.093 to 0.162 | 0.595 |
| Follow-up | -0.033 | -0.122 to 0.057 | 0.471 | -0.175 | -0.278 to -0.073 | 0.001 |

Abbreviations: ORR, objective response rate; DCR, disease control rate; CI, confidence interval; BCLC, Barcelona Clinic Liver Cancer.

**Table S14.2: Exploratory within-node meta-regression for DEB-TACE versus cTACE objective response**

**Note:** The coefficient is exponentiated and reported as a ratio of RRs. These post hoc univariable analyses use study-level classifications and are limited by incomplete drug/microsphere reporting, ecological bias, residual confounding, and low power. A non-significant interaction does not demonstrate equivalence among products.

| **Study-level covariate** | **Direct comparisons** | **Class / reference comparisons** | **Ratio of RRs (95% CI)** | **P value** |
| --- | --- | --- | --- | --- |
| Doxorubicin-based vs other/unclear chemotherapy | 34 | 7 / 27 | 0.96 (0.84-1.09) | 0.513 |
| CalliSpheres vs other/unspecified microspheres | 34 | 8 / 26 | 1.02 (0.88-1.18) | 0.795 |

**Appendix 15: Subgroup Analysis**

**Table S15.1: Subgroup analysis according to liver function reserve**

**Note:** Liver function reserve was classified according to the study-level proportion of patients with Child-Pugh class A. Studies with Child-Pugh A proportion <70% were classified as the lower liver-function-reserve subgroup, whereas studies with Child-Pugh A proportion ≥70% were classified as the preserved liver-function-reserve subgroup. Studies without extractable Child-Pugh A/B data were excluded from this subgroup analysis. Because several treatment nodes were sparsely connected within subgroups, these analyses were considered exploratory.

| **Outcome** | **Child-Pugh A proportion subgroup** | **Studies** | **Arms** | **Participants** | **Events** | **Treatment nodes included** |
| --- | --- | --- | --- | --- | --- | --- |
| ORR | <70% | 19 | 38 | 1653 | 900 | cTACE, DEB-TACE, TAE |
| ORR | ≥70% | 20 | 40 | 1835 | 1086 | cTACE, DEB-TACE, DSM-TACE, B-TACE, TAE, TARE |
| DCR | <70% | 19 | 38 | 1653 | 1257 | cTACE, DEB-TACE, TAE |
| DCR | ≥70% | 16 | 32 | 1552 | 1219 | cTACE, DEB-TACE, DSM-TACE, B-TACE, TAE, TARE |
| CR | <70% | 19 | 38 | 1653 | 314 | cTACE, DEB-TACE, TAE |
| CR | ≥70% | 18 | 36 | 1709 | 439 | cTACE, DEB-TACE, DSM-TACE, B-TACE, TAE, TARE |
| PR | <70% | 20 | 40 | 1695 | 630 | cTACE, DEB-TACE, TAE, TARE |
| PR | ≥70% | 20 | 40 | 1887 | 546 | cTACE, DEB-TACE, DSM-TACE, B-TACE, TAE, TARE |
| SD | <70% | 19 | 38 | 1653 | 373 | cTACE, DEB-TACE, TAE |
| SD | ≥70% | 17 | 34 | 1577 | 397 | cTACE, DEB-TACE, DSM-TACE, B-TACE, TAE, TARE |
| PD | <70% | 19 | 38 | 1653 | 395 | cTACE, DEB-TACE, TAE |
| PD | ≥70% | 18 | 36 | 1601 | 343 | cTACE, DEB-TACE, DSM-TACE, B-TACE, TAE, TARE |
| Nausea/vomiting | <70% | 12 | 24 | 1173 | 372 | cTACE, DEB-TACE, DSM-TACE |
| Nausea/vomiting | ≥70% | 10 | 20 | 932 | 151 | cTACE, DEB-TACE, TAE |
| Abdominal pain | <70% | 13 | 26 | 1179 | 481 | cTACE, DEB-TACE, DSM-TACE, TAE |
| Abdominal pain | ≥70% | 11 | 22 | 1283 | 436 | cTACE, DEB-TACE, B-TACE, TAE, TARE |
| Fever | <70% | 13 | 26 | 1241 | 440 | cTACE, DEB-TACE, DSM-TACE, TAE |
| Fever | ≥70% | 14 | 28 | 1396 | 373 | cTACE, DEB-TACE, B-TACE, TAE, TARE |

Abbreviations: ORR, objective response rate; DCR, disease control rate; CR, complete response; PR, partial response; SD, stable disease; PD, progressive disease; cTACE, conventional transarterial chemoembolization; DEB-TACE, drug-eluting bead transarterial chemoembolization; DSM-TACE, degradable starch microspheres transarterial chemoembolization; B-TACE, balloon-occluded transarterial chemoembolization; TAE, transarterial embolization; TARE, transarterial radioembolization.

**Appendix 16: Pairwise comparison forest plots**

**Figure S16.1: Pairwise comparison forest plot of objective response rate (ORR)**

Pairwise relative effects among transarterial treatment strategies for hepatocellular carcinoma are shown as risk ratios (RR) with 95% confidence intervals (CIs). The dashed vertical line represents no difference between treatments (RR = 1). An RR greater than 1 indicates a higher objective response rate for the treatment listed first in each comparison, whereas an RR less than 1 indicates a higher objective response rate for the comparator.

Abbreviations: c-TACE, conventional transarterial chemoembolization; DEB-TACE, drug-eluting bead transarterial chemoembolization; DSM-TACE, degradable starch microspheres transarterial chemoembolization; B-TACE, balloon occluded transarterial chemoembolization; TAE, transarterial embolization; TARE, transarterial radioembolization

**Figure S16.2: Pairwise comparison forest plot of disease control rate (DCR)**

Pairwise relative effects among transarterial treatment strategies for hepatocellular carcinoma are shown as risk ratios (RR) with 95% confidence intervals (CIs). The dashed vertical line represents no difference between treatments (RR = 1). An RR greater than 1 indicates a higher disease control rate for the treatment listed first in each comparison, whereas an RR less than 1 indicates a higher disease control rate for the comparator.

Abbreviations: c-TACE, conventional transarterial chemoembolization; DEB-TACE, drug-eluting bead transarterial chemoembolization; DSM-TACE, degradable starch microspheres transarterial chemoembolization; B-TACE, balloon occluded transarterial chemoembolization; TAE, transarterial embolization; TARE, transarterial radioembolization

**Figure S16.3: Pairwise comparison forest plot of complete response (CR)**

Pairwise relative effects among transarterial treatment strategies for hepatocellular carcinoma are shown as risk ratios (RR) with 95% confidence intervals (CIs). The dashed vertical line represents no difference between treatments (RR = 1). An RR greater than 1 indicates a higher complete response for the treatment listed first in each comparison, whereas an RR less than 1 indicates a higher complete response for the comparator.

Abbreviations: c-TACE, conventional transarterial chemoembolization; DEB-TACE, drug-eluting bead transarterial chemoembolization; DSM-TACE, degradable starch microspheres transarterial chemoembolization; B-TACE, balloon occluded transarterial chemoembolization; TAE, transarterial embolization; TARE, transarterial radioembolization

**Figure S16.4: Pairwise comparison forest plot of partial response (PR)**

Pairwise relative effects among transarterial treatment strategies for hepatocellular carcinoma are shown as risk ratios (RR) with 95% confidence intervals (CIs). The dashed vertical line represents no difference between treatments (RR = 1). An RR greater than 1 indicates a higher partial response for the treatment listed first in each comparison, whereas an RR less than 1 indicates a higher partial response for the comparator.

Abbreviations: c-TACE, conventional transarterial chemoembolization; DEB-TACE, drug-eluting bead transarterial chemoembolization; DSM-TACE, degradable starch microspheres transarterial chemoembolization; B-TACE, balloon occluded transarterial chemoembolization; TAE, transarterial embolization; TARE, transarterial radioembolization

**Figure S16.5: Pairwise comparison forest plot of stable disease (SD)**

Pairwise relative effects among transarterial treatment strategies for hepatocellular carcinoma are shown as risk ratios (RR) with 95% confidence intervals (CIs). The dashed vertical line represents no difference between treatments (RR = 1). An RR greater than 1 indicates a higher stable disease for the treatment listed first in each comparison, whereas an RR less than 1 indicates a higher stable disease for the comparator.

Abbreviations: c-TACE, conventional transarterial chemoembolization; DEB-TACE, drug-eluting bead transarterial chemoembolization; DSM-TACE, degradable starch microspheres transarterial chemoembolization; B-TACE, balloon occluded transarterial chemoembolization; TAE, transarterial embolization; TARE, transarterial radioembolization

**Figure S16.6: Pairwise comparison forest plot of progressive disease (PD)**

Pairwise relative effects among transarterial treatment strategies for hepatocellular carcinoma are shown as odds ratios (OR) with 95% confidence intervals (CIs). The dashed vertical line represents no difference between treatments (OR = 1). An OR greater than 1 indicates a higher progressive disease for the treatment listed first in each comparison, whereas an OR less than 1 indicates a higher progressive disease for the comparator.

Abbreviations: c-TACE, conventional transarterial chemoembolization; DEB-TACE, drug-eluting bead transarterial chemoembolization; DSM-TACE, degradable starch microspheres transarterial chemoembolization; B-TACE, balloon occluded transarterial chemoembolization; TAE, transarterial embolization; TARE, transarterial radioembolization

**Figure S16.7: Pairwise comparison forest plot of nausea, vomiting**

Pairwise relative effects among transarterial treatment strategies for hepatocellular carcinoma are shown as odds ratios (OR) with 95% confidence intervals (CIs). The dashed vertical line represents no difference between treatments (OR = 1). An OR greater than 1 indicates a higher progressive disease for the treatment listed first in each comparison, whereas an OR less than 1 indicates a higher progressive disease for the comparator.

Abbreviations: c-TACE, conventional transarterial chemoembolization; DEB-TACE, drug-eluting bead transarterial chemoembolization; DSM-TACE, degradable starch microspheres transarterial chemoembolization; TAE, transarterial embolization

**Figure S16.8: Pairwise comparison forest plot of abdominal pain**

Pairwise relative effects among transarterial treatment strategies for hepatocellular carcinoma are shown as odds ratios (OR) with 95% confidence intervals (CIs). The dashed vertical line represents no difference between treatments (OR = 1). An OR greater than 1 indicates a higher progressive disease for the treatment listed first in each comparison, whereas an OR less than 1 indicates a higher progressive disease for the comparator.

Abbreviations: c-TACE, conventional transarterial chemoembolization; DEB-TACE, drug-eluting bead transarterial chemoembolization; DSM-TACE, degradable starch microspheres transarterial chemoembolization; B-TACE, balloon occluded transarterial chemoembolization; TAE, transarterial embolization; TARE, transarterial radioembolization

**Figure S16.9: Pairwise comparison forest plot of fever**

Pairwise relative effects among transarterial treatment strategies for hepatocellular carcinoma are shown as odds ratios (OR) with 95% confidence intervals (CIs). The dashed vertical line represents no difference between treatments (OR = 1). An OR greater than 1 indicates a higher progressive disease for the treatment listed first in each comparison, whereas an OR less than 1 indicates a higher progressive disease for the comparator.

Abbreviations: c-TACE, conventional transarterial chemoembolization; DEB-TACE, drug-eluting bead transarterial chemoembolization; DSM-TACE, degradable starch microspheres transarterial chemoembolization; B-TACE, balloon occluded transarterial chemoembolization; TAE, transarterial embolization; TARE, transarterial radioembolization

**Appendix 17: Comparison with previous TACE-related network meta-analyses and evidence supporting DSM-TACE and B-TACE**

**Table S17.1. Structured comparison of previous and current TACE-related network meta-analyses**

| **Review** | **Evidence base** | **Network question and treatment nodes** | **Main outcomes** | **Relationship to the current review** |
| --- | --- | --- | --- | --- |
| Katsanos et al. (2017) ^[25]^ | 55 RCTs; 5,763 participants | Eight-node network including control, TAE, cTACE, DEB-TACE, TARE, and TACE combined with local ablation, external radiotherapy, or systemic agents. | Mortality/survival and tumor response. | Substantial overlap for cTACE, DEB-TACE, TAE, and TARE. DSM-TACE and B-TACE were not evaluated as distinct nodes. The present review is not a direct OS update because its procedural scope and outcome estimands differ. |
| Yan et al. (2023) ^[26]^ | 67 RCTs; eight TACE-based strategies | TACE alone and seven combination protocols, including combinations with sorafenib, radiotherapy, radiofrequency ablation, percutaneous ethanol injection, and iodine-125 implantation. | Short-term survival, response, and complications. | Addresses treatment intensification by adding another modality to TACE rather than comparing the arterial platforms themselves. |
| Zhang et al. (2022) ^[27]^ | RCTs and cohort studies | TACE combined with sorafenib, lenvatinib, apatinib, and other molecularly targeted agents. | OS, PFS, response, and adverse events. | Compares targeted-agent combinations; does not isolate the effects of cTACE, DEB-TACE, DSM-TACE, B-TACE, TAE, or TARE as procedural platforms. |
| Long et al. (2023) ^[28]^ | 45 studies; 10,774 participants; eight strategies | Different molecularly targeted agents combined with TACE. | OS, PFS, ORR, DCR, and adverse events. | Informs selection of the systemic partner added to TACE; includes predominantly non-randomized evidence and answers a different clinical question. |
| Le et al. (2025) ^[29]^ | 40 RCTs | TACE, HAIC, and TACE combined with RFA, MWA, HIFU, PEI, or radiotherapy. | Response, 1- and 2-year survival, and adverse events. | Compares TACE/HAIC and local combination strategies, not distinct embolization or radioembolization platforms. |
| Current review | 56 RCTs; 5,083 participants; search through May 1, 2026 | Six procedural nodes: cTACE, DEB/DEM-TACE, DSM-TACE, B-TACE, TAE, and TARE. Non-transarterial co-interventions were excluded unless balanced between randomized groups. | ORR, DCR, CR, PR, SD, PD, and common post-embolization symptoms. | Updates randomized procedural-platform evidence; adds distinct DSM-TACE and B-TACE nodes; uses RoB 2, CINeMA, transitivity assessment, and sensitivity analyses. The incremental conclusion is limited by sparse DSM-TACE and B-TACE evidence. |

Abbreviations: B-TACE, balloon-occluded transarterial chemoembolization; cTACE, conventional transarterial chemoembolization; DCR, disease control rate; DEB-TACE, drug-eluting bead transarterial chemoembolization; DSM-TACE, degradable starch microsphere transarterial chemoembolization; HAIC, hepatic arterial infusion chemotherapy; HIFU, high-intensity focused ultrasound; MWA, microwave ablation; ORR, objective response rate; OS, overall survival; PEI, percutaneous ethanol injection; PFS, progression-free survival; RCT, randomized controlled trial; RFA, radiofrequency ablation; TAE, transarterial embolization; TARE, transarterial radioembolization.

**Table S17.2. Randomized evidence supporting the DSM-TACE and B-TACE nodes**

Node totals: DSM-TACE was supported by 2 RCTs with 121 randomized participants (63 allocated to DSM-TACE), both published after the Katsanos review. B-TACE was supported by 3 RCTs with 214 randomized participants (104 allocated to B-TACE); only the 2026 trial (60 participants) was newly published after 2017, whereas the two older trials (154 participants) were available chronologically but were not represented as a distinct B-TACE node. Combined, the two nodes comprised 5 RCTs and 335 randomized participants (167 allocated to DSM-TACE or B-TACE), of which 3 RCTs and 181 participants were published after 2017. The two older B-TACE trials were retained in the primary analysis as clinically indirect and excluded in a post hoc sensitivity analysis.

| **Node and study** | **Year** | **Comparison** | **Randomized N (active-node N)** | **Availability to Katsanos et al. (2017)** | **Interpretive relevance** |
| --- | --- | --- | --- | --- | --- |
| DSM-TACE: Gjoreski et al. | 2020 | DSM-TACE vs cTACE | 60 (32) | Unavailable; published after the 2017 search. | HCC-specific randomized evidence; contributes to a two-trial, sparsely connected node. |
| DSM-TACE: Vogl et al. | 2021 | DSM-TACE vs cTACE | 61 (31) | Unavailable; published after the 2017 search. | HCC-specific randomized evidence; contributes to a two-trial, sparsely connected node. |
| B-TACE: Shan et al. | 2026 | B-TACE vs cTACE | 60 (30) | Unavailable; published after the 2017 search. | Contemporary HCC-specific trial, but small and enriched for large/advanced tumors. |
| B-TACE: Yang et al. | 2012 | B-TACE vs cTACE | 80 (40) | Chronologically available, but B-TACE was not represented as a distinct node. | One of two older clinically indirect trials; the pair enrolled hypovascular or mixed liver-tumor populations. |
| B-TACE: Cheng et al. | 2004 | B-TACE vs cTACE | 74 (34) | Chronologically available, but B-TACE was not represented as a distinct node. | One of two older clinically indirect trials; the pair enrolled hypovascular or mixed liver-tumor populations. |
